# Screening of factors inducing alveolar type 1 epithelial cells using human pluripotent stem cells

**DOI:** 10.1016/j.stemcr.2024.02.009

**Published:** 2024-03-28

**Authors:** Yuko Ohnishi, Atsushi Masui, Takahiro Suezawa, Ryuta Mikawa, Toyohiro Hirai, Masatoshi Hagiwara, Shimpei Gotoh

**Affiliations:** 1 Department of Drug Discovery for Lung Diseases, Graduate School of Medicine, Kyoto University, Kyoto 606-8501, Japan; 2 Center for iPS Cell Research and Application (CiRA), Kyoto University, Kyoto 606-8507, Japan; 3 Department of Respiratory Medicine, Graduate School of Medicine, Kyoto University, Kyoto 606-8507, Japan; 4 Department of Anatomy and Developmental Biology, Graduate School of Medicine, Kyoto University, Kyoto 606-8501, Japan

**Keywords:** iPSC, alveolar epithelial differentiation, high-throughput screening, YAP/TAZ, AKT, lung regeneration, organoid

## Abstract

Alveolar type 2 (AT2) epithelial cells are tissue stem cells capable of differentiating into alveolar type 1 (AT1) cells for injury repair and maintenance of lung homeostasis. However, the factors involved in human AT2-to-AT1 cell differentiation are not fully understood. Here, we established *SFTPC*^*GFP*^ and *AGER*^*mCherry-HiBiT*^ dual-reporter induced pluripotent stem cells (iPSCs), which detected AT2-to-AT1 cell differentiation with high sensitivity and identified factors inducing AT1 cell differentiation from AT2 and their progenitor cells. We also established an “on-gel” alveolar epithelial spheroid culture suitable for medium-throughput screening. Among the 274 chemical compounds, several single compounds, including LATS-IN-1, converted AT1 cells from AT2 and their progenitor cells. Moreover, YAP/TAZ signaling activation and AKT signaling suppression synergistically recapitulated the induction of transcriptomic, morphological, and functionally mature AT1 cells. Our findings provide novel insights into human lung development and lung regenerative medicine.

## Highlights

• An *mCherry-HiBiT* reporter iPSC line was useful for the screening of AT1 cell inducers
• On-gel alveolar epithelial spheroid culture facilitated medium-throughput screening
• YAP/TAZ activation and AKT inhibition synergistically promoted AT1 differentiation

## Graphical abstract

**Figure:**
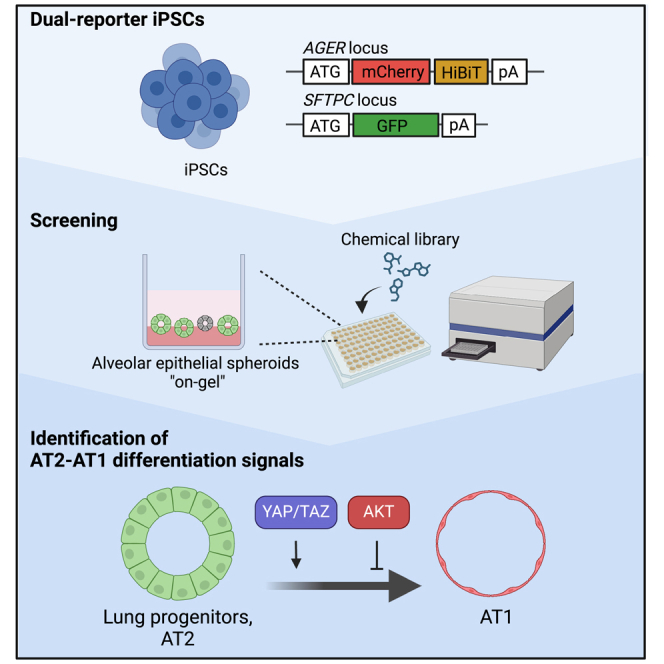

## Teaser

In the alveolar epithelium, AT2 cells maintain homeostasis and regeneration through self-renewal and differentiation into AT1 cells. A dual-reporter cell line and on-gel alveolar epithelial spheroid culture were established to quantitatively evaluate AT1 differentiation. Our findings showed that YAP/TAZ activation and AKT inhibition synergistically promoted AT1 cells, thereby providing novel insights into AT1 differentiation and contributing to lung regenerative medicine.

## Introduction

Lung alveoli are a highly functional part of the lung and consist of 300 million small pouch-like structures. The alveolar epithelium comprises alveolar type 2 (AT2) and alveolar type 1 (AT1) epithelial cells. AT2 cells are cuboidal in shape and secrete pulmonary surfactants, which maintain the alveolar structure by reducing alveolar surface tension. AT2 cells are tissue stem cells that maintain alveolar homeostasis and regeneration after injury via self-renewal and differentiation into AT1 cells (Barkauskas et al., 2013). AT1 cells are thin and flat, covering 95% of the alveolar surface, and are responsible for gas exchange. AT1 cells differentiate from bipotent alveolar epithelial progenitor cells in the fetal lung (Desai et al., 2014; Zepp et al., 2021). Abnormal alveolar epithelial cells with transitional profiles between AT2 and AT1 cells exist in the lungs, such as idiopathic pulmonary fibrosis, and in *in vivo* models such as bleomycin mice (Choi et al., 2020; Kobayashi et al., 2020; Strunz et al., 2020), suggesting the abnormalities of the differentiation process in the diseased lungs.

Signals that regulate the differentiation of AT2-to-AT1 cells have been explored. Among them, the activation of yes-associated protein and the transcriptional coactivator with PDZ-binding motif (YAP/TAZ) signaling is essential for AT1 cell differentiation (DiGiovanni et al., 2023; Gokey et al., 2021; LaCanna et al., 2019; Liu et al., 2016; Nguyen et al., 2021; Penkala et al., 2021; Warren et al., 2023). Other signals such as Notch, transforming growth factor β (TGF-β), Wnt, and p53 are also involved in AT2-to-AT1 cell differentiation (Finn et al., 2019; Frank et al., 2016; Kaiser et al., 2023; Kanagaki et al., 2021; Zhao et al., 2013). However, most reports are based on mouse studies and have yet to be validated enough using human alveolar epithelial cells. Historically, human AT2 cell lines for studying AT2-to-AT1 cell differentiation are lacking. Human primary AT2 cells are available for long-term culture in organoids; however, their limited supply has hampered an in-depth study of AT1 cells. Data on the human AT2-to-AT1 cell differentiation pathway remain limited. Human induced pluripotent stem cells (iPSCs) are useful for studying human alveolar epithelial cells (Gotoh et al., 2014; Yamamoto et al., 2017). A long-term culture of iPSC-derived AT2 (iAT2) cells was established, whereas iPSC-derived AT1 (iAT1) cells depended on the culture system. In Matrigel-embedded organoids, AT1 cell marker genes increased in coculture with human fetal lung fibroblasts (HFLFs), whereas they were almost negative under feeder-free conditions (Alysandratos et al., 2022; Kanagaki et al., 2021). iPSC-derived mesenchymal cells induced iAT2 and iAT1 cells from both carboxypeptidase M^+^ (CPM^+^) lung progenitor cells and iAT2 cells (Tamai et al., 2022), indicating that differentiation into iAT1 cells requires signaling from a coculture with mesenchymal cells. Based on these findings, we screened compounds that convert alveolar epithelial cell fate in a nonbiased setting without feeder mesenchymal cells. In this study, we established a dual-reporter iPSC line harboring *SFTPC*^*GFP*^ and *AGER*^*mCherry-HiBiT*^ to evaluate AT2-to-AT1 cell differentiation quantitatively with high sensitivity and established an “on-gel” culture of iAT2 cells suitable for medium-throughput screening. Combining dual-reporter iPSCs and on-gel culture, we screened 274 compounds to find those that increased HiBiT luminescence, leading to comprehensive signaling pathways. Among these, LATS-IN-1, an activator of YAP/TAZ signaling via inhibition of LATS1/2 kinases, and BAY1125976, an AKT inhibitor, synergistically promoted AT1 cell differentiation.

## Results

### Generation of a dual-reporter iPSC line with *SFTPC*^*GFP*^ and *AGER*^*mCherry-HiBiT*^

**Figure 1:**
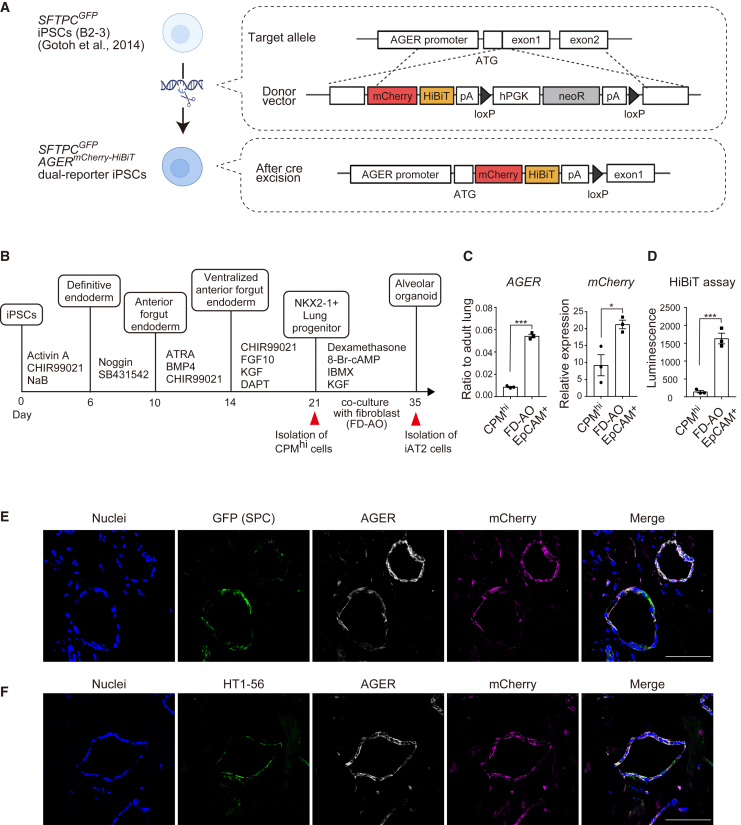
Generation of an *SFTPC*^*GFP*^*AGER*^*mCherry-HiBiT*^ dual-reporter iPSC line (A) Schematic illustration of the strategy used to build a dual-reporter iPSC line by gene editing. (B) Schematic diagram of the stepwise differentiation from iPSCs into iAT2 cells. (C) Comparison of mRNA expression levels in CPM^hi^ lung progenitor cells and MACS-isolated EpCAM^+^ cells from FD-AOs on day 35 (B). Data are shown as mean ± SEM (n = 3 from independent experiments). Unpaired 2-tailed Student’s t test; ^∗^p < 0.05; ^∗∗∗^p < 0.005. (D) Comparison of HiBiT luminescence between CPM^hi^ lung progenitor cells and MACS-isolated EpCAM^+^ cells from FD-AOs on day 35 (B). Data are shown as mean ± SEM (n = 3 from independent experiments). Unpaired 2-tailed Student’s t test; ^∗∗∗^p < 0.005. (E and F) Immunofluorescence imaging of FD-AOs. Scale bars: 100 μm.

To quantitatively detect AT1 cell differentiation in a highly sensitive manner, we generated a dual-reporter iPSC line by knocking in the *mCherry-HiBiT* gene at the locus of *AGER* in the *SFTPC*^*GFP*^ reporter iPSC line (B2-3 clone used for detecting AT2 cells) (Gotoh et al., 2014) using the CRISPR-Cas9 system (Figure 1A). *AGER*, expressed in AT1 cells, is a marker gene in human and mouse AT1 cells (Chung and Hogan, 2018; Dahlin et al., 2004; Guo et al., 2023). PCR confirmed that the reporter gene was knocked in into a single allele and that the drug resistance gene cassette was removed by introducing Cre recombinase (Figure S1A). The karyotype was normal (Figure S1B). The dual-reporter iPSCs were differentiated into CPM^hi^ lung progenitor cells, as previously described (Yamamoto et al., 2017), and the induction efficiency was similar to that of the parental cell line (Figures 1B and S1C). Alveolarized epithelial cells cocultured with HFLF showed increased *AGER* and *mCherry* gene expression and HiBiT luminescence (Figures 1C and 1D). Immunofluorescence staining showed that almost all of the AGER^+^ cells were identical to mCherry^+^ cells and that AGER^+^ cells expressed HT1-56, another AT1 cell marker (Figures 1E and 1F).

### On-gel culture of fibroblast-free alveolar epithelial spheroids for screening

**Figure 2:**
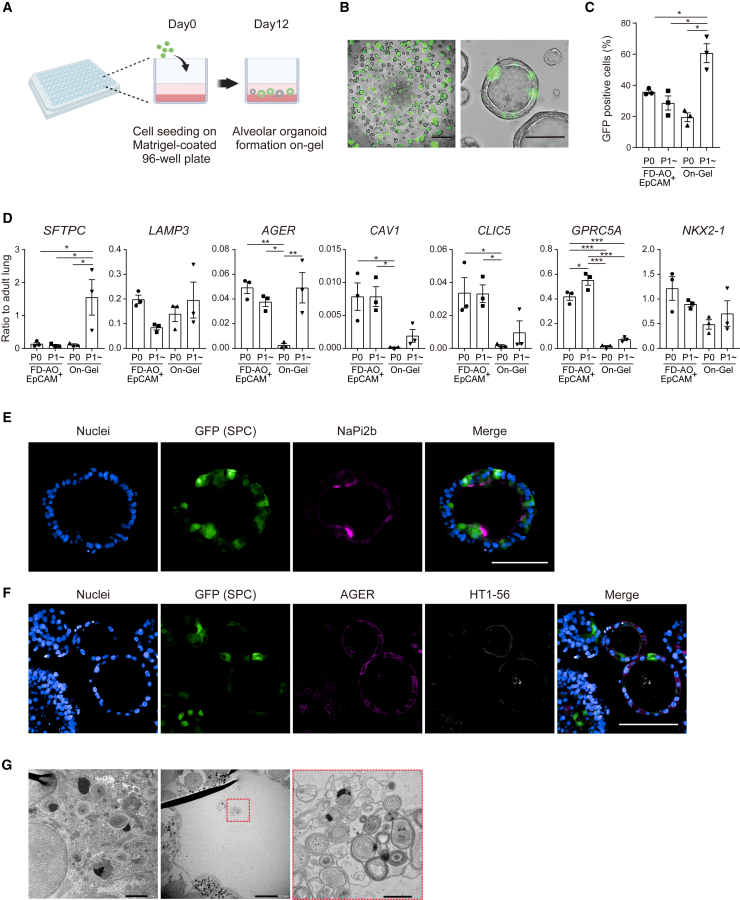
On-gel alveolar epithelial spheroid culture (A) Schematic representation of the on-gel culture. (B) Live-cell imaging of on-gel culture of alveolar spheroids derived from GFP^+^ iAT2 cells. Left scale bar, 500 μm; right scale bar, 100 μm. (C) GFP^+^ rate of epithelial cells in FD-AOs and on-gel alveolar spheroids. Data were obtained from passage 0 (P0) CPM^hi^ lung progenitor cells or P1-4 GFP^+^ iAT2 cell-derived cells. Data are shown as mean ± SEM (n = 3 from independent experiments). One-way ANOVA with Tukey’s multiple comparison test; ^∗^p < 0.05. (D) mRNA expression in the epithelial cells of FD-AOs and on-gel alveolar spheroids (P0 or P1-4). P1-4 on-gel alveolar spheroids were derived from P0-3 FD-AOs. Data are shown as mean ± SEM (n = 3 from independent experiments). One-way ANOVA with Tukey’s multiple comparison test; ^∗^p < 0.05; ^∗∗^p < 0.01; ^∗∗∗^p < 0.005. (E and F) Whole-mount immunofluorescent imaging of the on-gel alveolar spheroids on-gel derived from P1. Scale bars, 100 μm. (G) Transmission electron microscopy imaging of lamellar bodies and tubular myelin-like structures of on-gel iAT2 cells. Left scale bar, 2 μm; center scale bar, 10 μm; right scale bar, 1 μm.

Since our previously reported fibroblast-dependent alveolar organoids (FD-AOs) and fibroblast-free alveolar organoids (FF-AOs) were embedded in Matrigel, they were not ideal for screening due to the requirement for careful mixing of the cells in Matrigel (Yamamoto et al., 2017). In addition, air-liquid interface cultures need 520,000 cells/cm^2^ to apply these culture methods to screening (Hekman et al., 2020). We established a new alveolar epithelial cell culture method, on-gel culture, for compound screening. A total of 5 × 10^3^ of CPM^hi^ lung progenitor cells or 1 × 10^4^ of GFP^+^ iAT2 cells were seeded on thick-coated Matrigel in 96-well plates and cultured in DCIK+2i (CHIR99021 and SB431542) medium for 12 days, resulting in the formation of spheroids on Matrigel, exposing themselves to culture medium (Figures 2A and 2B). GFP fluorescence indicating the presence of iAT2 cells showed that the rate of GFP^+^ cells was significantly higher in iAT2 cell-derived on-gel spheroids than FD-AOs (Figures 2B and 2C). *SFTPC* expression was markedly higher in on-gel culture than FD-AOs up to the level in human adult lungs (Figure 2D). Another AT2 cell marker, *LAMP3*, and a lung epithelial cell marker, *NKX2-1*, did not differ significantly between the two culture methods. Although *AGER* expression in iAT2 cell-derived on-gel spheroids (passages 1-3) was equivalent between the FD-AOs, the expression levels were low compared to those in the adult lung. The expression of other AT1 cell markers, such as *CAV1*, *CLIC5*, and *GPRC5A* was lower in the on-gel culture than FD-AOs, indicating that iAT1 cells were barely present or immature in the on-gel culture (Figure 2D). Immunofluorescence staining confirmed the presence of iAT2 cells and AT1 marker-positive cells (Figures 2E and 2F). RNA sequencing (RNA-seq) analysis showed that on-gel spheroids tended to express the highest levels of AT2 and club cell marker genes among CPM^hi^ lung progenitor cells and epithelial cells from FD-AOs and FF-AOs (Figures S2A and S2B). FD-AOs expressed AT1 and airway epithelial cell marker genes more strongly than FF-AOs and on-gel spheroids. In contrast, FF-AOs expressed each lineage marker gene at a lower level compared with FD-AOs and on-gel spheroids. Venn diagrams of the differentially expressed genes (DEGs) extracted from each culture method showed that 370 genes were specifically upregulated in the on-gel culture, and Gene Ontology (GO) analysis showed that GOs related to lipid synthesis and metabolism, which are characteristics of AT2 cells, were enriched (Figures S2C and S2D). Furthermore, lamellar body structures, a characteristic of functional AT2 cells, were observed in the cytoplasm of on-gel-cultured iAT2 cells, and tubular myelin-like structures were observed on the luminal side of the spheroid (Figure 2G). In contrast, 895 genes were explicitly reduced in the on-gel culture, and GOs related to DNA replication and cell proliferation were enriched, indicating that on-gel culture is characterized by a lower proliferative potential of iAT2 cells compared to the other two culture methods (Figures S2E and S2F). These data demonstrate that on-gel culture is suitable for the culture of alveolar spheroids (which consist of epithelial cells alone) and it requires only a small number of cells, allowing the culture of iAT2 cells with easy handling.

### Compound screening for searching AT2-to-AT1 cell differentiation signal

**Figure 3:**
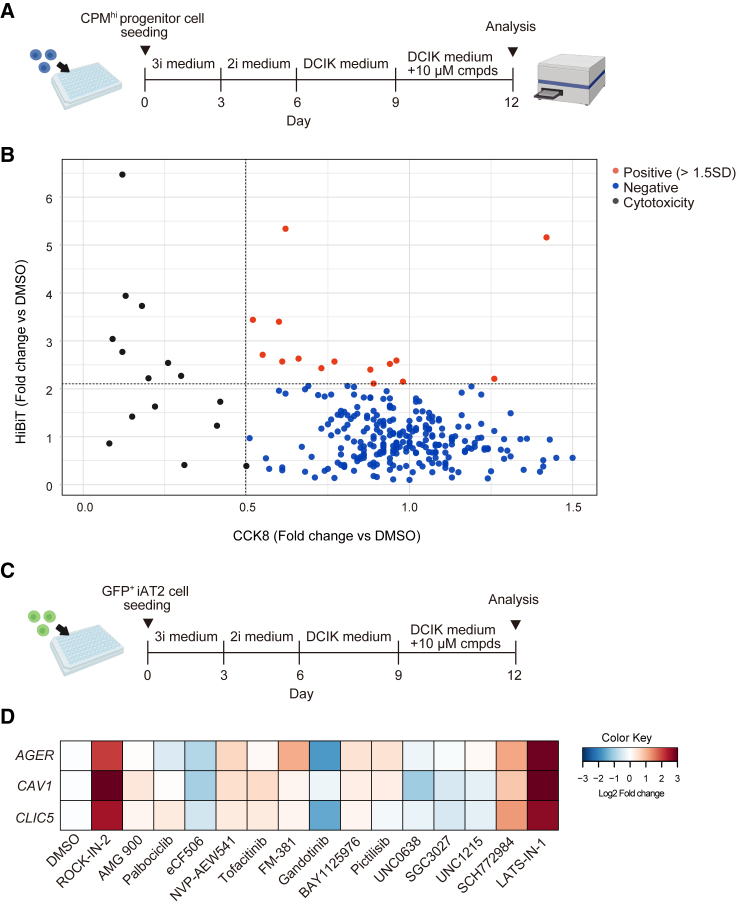
Compound screening using the *SFTPC*^*GFP*^*AGER*^*mCherry-HiBiT*^ dual-reporter iPSC-derived alveolar epithelial cells (A) Schematic illustration of the strategy used for compound screening using the dual-reporter iPSC-derived CPM^hi^ lung progenitor cells. (B) Compound screening using HiBiT luminescence and CCK8 absorbance as indicators. Compounds with CCK8 values <0.5 times the value of DMSO were excluded as cytotoxic, and 1.5 times the SD value of HiBiT/CCK8 of the remaining compounds was used as the hit criteria. (C) Schematic illustration of the strategy used for the validation of the hit compounds using dual-reporter iPSC-derived GFP^+^ iAT2 cells. (D) mRNA expression of on-gel GFP^+^ iAT2 cell-derived spheroids treated with 10 μM hit compounds. The heatmap was created with the value of log_2_ (fold change vs DMSO). n = 3 from independent experiments.

We screened for compounds that convert AT2-to-AT1 cells by combining dual-reporter iPSCs and on-gel culture method. Due to the readily available cell numbers, we used CPM^hi^ lung progenitor cells for screening (Figure 3A). The chemical library of 274 compounds (Table S1) was applied at 10 μM for 3 days, and the compounds were narrowed down using the HiBiT luminescence values corrected using the Cell Counting Kit 8 (CCK8) absorbance values as an indicator. As a result, 15 compounds exceeded the hit criteria, >1.5 SD, whereas 15 were judged to have high cytotoxicity based on the CCK8 values (Figure 3B). These hit compounds were validated for their effects on AT1 marker gene expression in GFP^+^ iAT2 cell-derived spheroids (Figures 3C and 3D). Among these compounds, LATS-IN-1 upregulated *AGER*, *CLIC5*, and *CAV1*. LATS-IN-1, an inhibitor of LATS1 and LATS2, activates YAP/TAZ signaling by inhibiting YAP/TAZ phosphorylation (Kastan et al., 2021). YAP/TAZ signaling is essential for AT1 cell differentiation, and recently, LATS-IN-1 reportedly promoted the differentiation of iPSC-derived AT2 cells into AT1 cells (Burgess et al., 2023). However, LATS-IN-1 clumped the spheroids and promoted cell proliferation, which did not induce cell thinning or quiescence, a hallmark of AT1 cells (Figures S3A–S3D). ROCK-IN-2, an inhibitor of rho-associated protein kinase 2 (ROCK2), elevated AT1 gene expression and clumped spheroids, similar to LATS-IN-1. Since a recent study reported that stretching increased the number of AT1 cells via the ROCK-YAP pathway in mice (Nguyen et al., 2021), we did not expect that ROCK-IN-2 promoted AT1 differentiation. To determine whether ROCK-IN-2 promotes AT1 differentiation via on-target effects, Y-27632 and Fasudil HCl, other ROCK inhibitors, were tested. However, these compounds did not upregulate AT1 gene expression (Figures S3E and S3F). Furthermore, ROCK-IN-2 upregulated the expression of genes downstream of YAP/TAZ signaling, *CCN2*, *ANKRD1*, and *CYR61*, suggesting ROCK-IN-2 as an off-target regulator of YAP/TAZ signaling. NVP-AEW541 upregulated AT1 marker gene expression but was excluded from further studies because it was considered cytotoxic in iAT2 cells based on CCK8 values and cell morphology.

### Combination of active YAP/TAZ signaling with AKT signal suppression promotes AT2-to-AT1 cell differentiation

**Figure 4:**
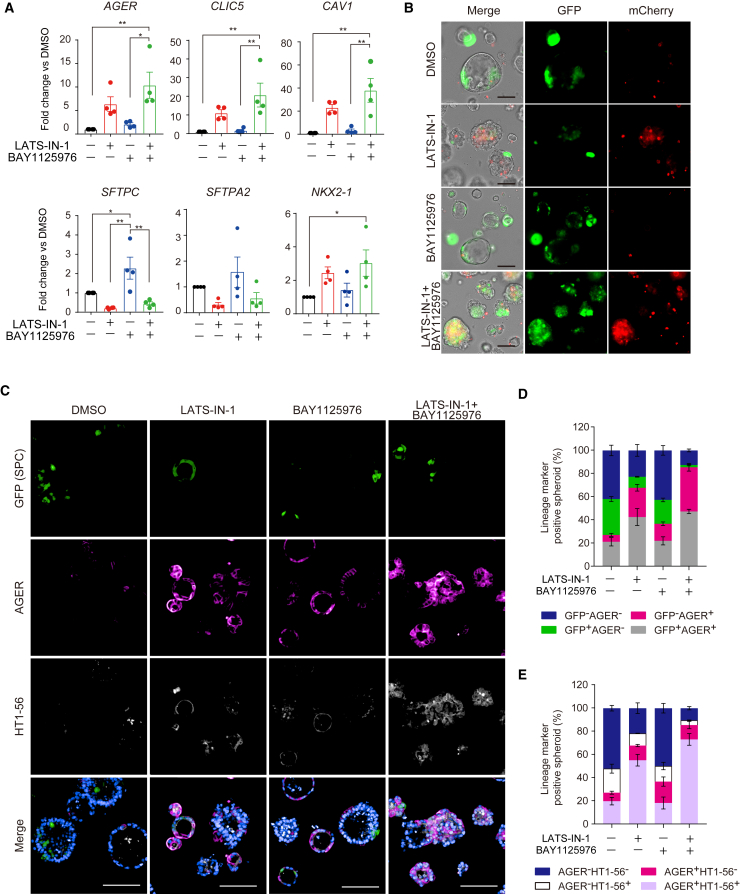
Synergistic effect of YAP/TAZ activation and AKT suppression on AT2-to-AT1 cell differentiation (A) mRNA expression in dual-reporter iAT2 cell-derived on-gel spheroids (P1). Each column represents samples treated with 10 μM LATS-IN-1, BAY1125976, or both. Data are shown as mean ± SEM (n = 3 from independent experiments). One-way ANOVA with Tukey’s multiple comparison test; ^∗^p < 0.05; ^∗∗^p < 0.01. (B) Live-cell imaging of iAT2 cell-derived on-gel spheroids treated with the compounds. Scale bars, 100 μm. (C) Whole-mount immunofluorescence imaging of on-gel spheroids derived from GFP^+^ iAT2 using *SFTPC*^*GFP*^ reporter iPSC. Scale bars, 100 μm. (D and E) Quantitative analysis of whole-mount images shown in (C). Data are shown as mean ± SEM (n = 3 from independent experiments using P1-2 iAT2 derived on-gel spheroids).

Tofacitinib, FM381, BAY1125976, and SCH772984 were suspected to upregulate the three AT1 marker genes (Figure 3D). However, since the efficacy of each of these compounds was weak, we evaluated their synergistic effect in combination with the activation of YAP/TAZ signaling for AT1 cell differentiation. Among these compounds, only BAY1125976, an allosteric inhibitor of AKT, and LATS-IN-1 synergistically increased the expression of AT1 cell marker genes (Figures 4A and S4A). AKT signaling functions as a hub for multiple pathways, including cell survival and proliferation, and is important for AT2 cell proliferation (Portnoy et al., 2004). A few reports have suggested its involvement in AT2-to-AT1 cell differentiation (Zhang et al., 2023; Zhong et al., 2022), although it has not been sufficiently evaluated. Furthermore, since recent transcriptomic analysis indicated that AKT signaling can be affected in the transitional state of alveolar epithelial cells observed in damaged fibrotic lungs (Kobayashi et al., 2020; Xu et al., 2016), we hypothesized that AKT signaling may be associated with AT2-to-AT1 cell differentiation. Since YAP signaling has been reported to positively regulate AKT signaling (Gokey et al., 2018), it is intriguing that AKT inhibition may be involved in AT1 differentiation in concert with LATS-IN-1. We determined the optimal concentrations of LATS-IN-1 and BAY1125976 to activate YAP/TAZ and inhibit AKT, respectively. Phosphorylated YAP was decreased in a LATS-IN-1 dose-dependent manner, whereas total YAP expression peaked at 10 μM; hence, subsequent experiments were performed at 10 μM (Figure S4B). BAY1125976 inhibited phosphorylated AKT at 10 μM; therefore, subsequent experiments were set at 10 μM (Figure S4C). Since AKT signaling suppression triggers apoptosis (Coffey et al., 2005), the initiation of apoptosis may have influenced the AT1 cell marker gene expression. To test this, we investigated whether the two apoptotic inducers, Camptothecin and Staurosporine, contribute to AT1 cell marker gene expression. The apoptotic inducers and BAY1125976 significantly increase caspase activity; however, camptothecin and staurosporine did not increase AT1 cell marker gene expression (Figures S4D and S4E). Furthermore, Alpelisib and AZD6482, PI3K (phosphatidylinositol 3-kinase)/AKT pathway inhibitors, upregulated AT1 cell marker gene expression synergistically with LATS-IN-1 (Figure S4F). Therefore, we focused on the relationship between YAP/TAZ and AKT signaling using LATS-IN-1 and BAY1125976.

Along with the increased expression of *AGER* and other AT1 marker genes, cotreatment with the two compounds enhanced mCherry fluorescence (Figure 4B). Interestingly, BAY1125976 increased *SFTPC* gene expression, and cotreatment with the two compounds slightly increased its expression compared to that of LATS-IN-1 alone, suggesting that BAY1125976 suppressed cell proliferation and maintained AT2 marker gene expression. The immunofluorescence data showed that LATS-IN-1 and its combination with BAY1125976 decreased the number of GFP single-positive spheroids and increased the number of AT1 marker-positive spheroids (Figures 4C–4E). In particular, in treatment with the combination of LATS-IN-1 and BAY1125976, 89.3% of the spheroids were positive for at least either AGER or HT1-56. NKX2-1 was expressed in all of the cells derived from the repeatedly passaged GFP^+^ cells, suggesting that alveolar epithelial cell lineage was maintained (Figure S4G). Among the GFP and AGER double-negative cells, surfactant protein B (SPB)-positive cells were also present, suggesting that immature or distal lung AT2 subpopulations were present in the spheroids (Figure S4H).

**Figure 5:**
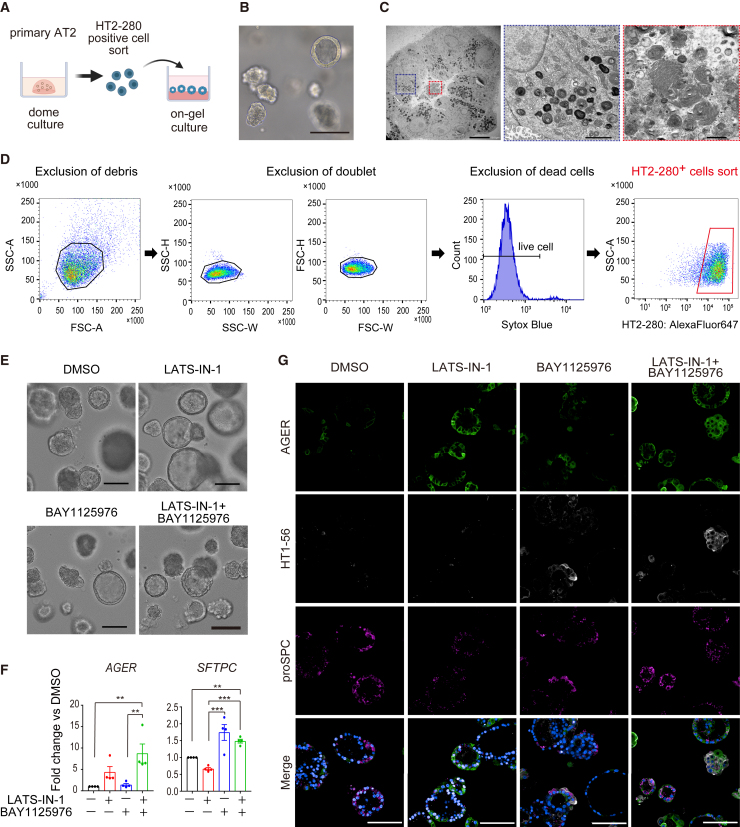
Validation of hit compounds using human adult primary AT2 cells (A) Schematic representation of maintenance and on-gel culture of primary AT2 cells. (B) Live-cell imaging of maintenance culture of primary AT2 cells. Scale bar, 100 μm. (C) Transmission electron microscopy imaging of primary AT2 cell maintenance culture. Left: overall view of the spheroid. Scale bar, 10 μm. Center: enlarged view of the area boxed in the blue square on the left. Imaging of lamellar bodies in cells. Scale bar, 2 μm. Right: enlarged view of the area boxed in the red square on the left. Imaging of tubular myelin-like structures in the spheroid lumen. Scale bar, 500 nm. (D) Gating strategy of flow cytometry for isolating HT2-280^+^ primary AT2 cells. (E) Live-cell imaging of each compound-treated spheroid derived from primary AT2 cells. Scale bars, 100 μm. (F) mRNA expression in on-gel primary AT2 cell-derived alveolar spheroids (P1-4). Each column represents samples treated with DMSO, 10 μM LATS-IN-1, 10 μM BAY1125976, or both. Data are shown as mean ± SEM (n = 4 from independent experiments). One-way ANOVA with Tukey’s multiple comparison test; ^∗∗^p < 0.01, ^∗∗∗^p < 0.005. (G) Whole-mount immunofluorescent imaging. Scale bars, 100 μm.

To confirm the reproducibility, another iPSC line, ChiPSC18, was differentiated into CPM^hi^ lung progenitor cells, and the isolated CPM^hi^ cells were cocultured with HFLFs to generate FD-AOs (Figures S5A and S5B). After 2 weeks of coculture, EpCAM^+^ NaPi2b^+^ iAT2 cells were isolated using fluorescence-activated cell sorting (FACS) and subjected to on-gel culture to test the compounds (Figure S5C). Unlike the dual-reporter iPSC line, no conspicuous clumped morphological changes were observed following LATS-IN-1 treatment (Figure S5D). As expected, AT1 marker gene expression was significantly increased following cotreatment with both compounds (Figure S5E). Furthermore, we determined whether these findings could be reproduced in human primary AT2 cells. We observed intracellular lamellar bodies and tubular myelin-like structures on the luminal side of the spheroids after 2 weeks of dome culture, suggesting that functional AT2 cells were maintained in the spheroids (Figures 5A–5C). We isolated HT2-280^+^ AT2 cells, subjected them to on-gel culture, and treated the spheroids with the above-mentioned compounds (Figure 5D). In contrast to iPSC, no increase in clumped forms was observed in primary AT2 cell-derived spheroids by LATS-IN-1 treatment (Figure 5E). A synergistic upregulation of the *AGER* gene was observed upon compound cotreatment, consistent with the immunofluorescence staining (Figures 5F and 5G).

### Mechanistic analysis of on-gel spheroids upon compound treatment

**Figure 6:**
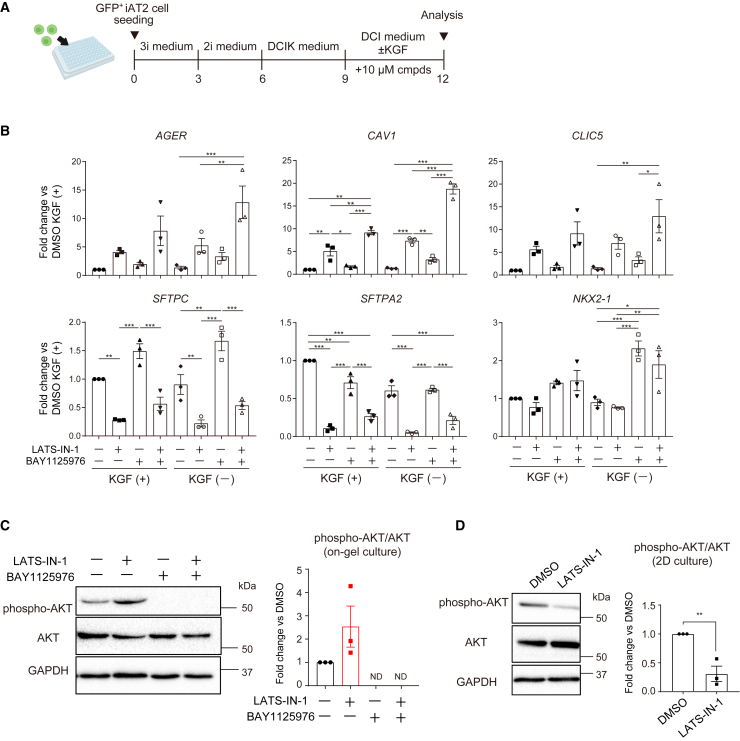
Mechanistic analysis of on-gel spheroids upon compound treatment (A) Schematic illustration of the strategy used to analyze the impact of KGF in the culture medium on AT1 cell differentiation. (B) AT1 and AT2 marker gene expression in *SFTPC*^*GFP*^*AGER*^*mCherry-HiBiT*^ dual-reporter iAT2 cell-derived on-gel spheroids (P1). Each column represents samples treated with 10 μM LATS-IN-1, BAY1125976, or both, with or without KGF. Data are shown as mean ± SEM (n = 3 from independent experiments). One-way ANOVA with Tukey’s multiple comparison test was used for analysis. Among several significant differences, only the comparison between the KGF contained group and KGF uncontained group was indicated; ^∗^p < 0.05; ^∗∗^p < 0.01; ^∗∗∗^p < 0.005. (C) Western blotting of the on-gel alveolar epithelial spheroids using *SFTPC*^*GFP*^ reporter iPSC-derived CPM^hi^ lung progenitor cells. Spheroids were treated with 10 μM LATS-IN-1, 10 μM BAY1125976, or both. Phospho (Ser473)-AKT and total AKT bands were quantified and phospho-AKT bands were normalized to total AKT bands. Data are shown as mean ± SEM (n = 3 from independent experiments). ND, not detected. (D) Western blotting of the 2D-cultured CPM^hi^ lung progenitor cells derived from *SFTPC*^*GFP*^ reporter iPSCs. Cells were treated with DMSO or 10 μM LATS-IN-1. Phospho (Ser473)-AKT and total AKT bands were quantified and phospho-AKT bands were normalized to total AKT bands. Data are shown as mean ± SEM (n = 3 from independent experiments).

AKT is downstream of keratinocyte growth factor (KGF) and suppresses the AT1 cell differentiation (Borok et al., 1998). Therefore, it is also possible that this observation is attributed to the incorporation of KGF in the differentiation media. However, even upon the removal of KGF from the media, the synergistic impact of the two compounds persisted, and the effect was augmented (Figures 6A and 6B). The absence of KGF did not induce changes in AT1 cell gene expression or reduce *SFTPC* expression. However, the removal of KGF decreased *SFTPA2* expression, implying that although KGF is essential for AT2 cell maintenance, it is not directly involved in the differentiation into AT1 cells. Increased phosphorylated AKT was observed under LATS-IN-1 treatment in three-dimensional (3D) culture (Figure 6C), suggesting that YAP/TAZ activation is essential for AT2-to-AT1 cell differentiation but may also activate AKT and interrupt the complete differentiation process. Intriguingly, AKT phosphorylation was spontaneously reduced under two-dimensional (2D) culture conditions with LATS-IN-1 treatment, in which cells were flattened, compared with the DMSO-treated cells (Figure 6D), suggesting that the regulation of AKT by YAP/TAZ depends on the microenvironment.

### AT1 cell maturation is promoted by active YAP/TAZ with suppressed AKT signaling

**Figure 7:**
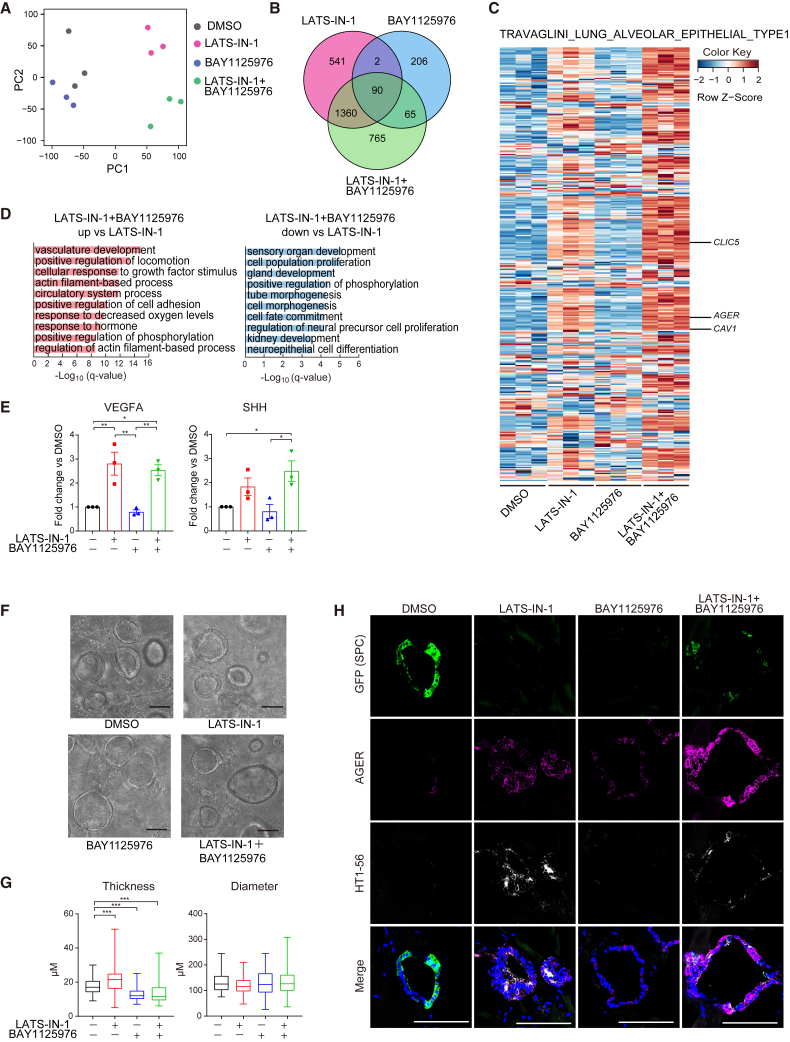
Effects of cotreatment with LATS-IN-1 and BAY1125976 on AT1 cell function and morphology (A) PCA of the transcriptomes of iAT2 cell-derived on-gel spheroids under each treatment condition. Log_2_ (transcripts per kilobase million [TPM] values) were used for the analysis. Data were obtained from 3 independent experiments. (B) Venn diagram of DEGs that were upregulated in compound-treated cells compared with DMSO-treated cells. The threshold for upregulation was set to log_2_ (fold-change) > 1, with an adjusted p value of <0.05. (C) Heatmap of TRAVAGLINI LUNG ALVEOLAR EPITELIAL TYPE1 created with the value of log_2_ (TPM + 0.01). Data were obtained from 3 independent experiments. (D) Enrichment analysis based on GO biological processes. Left: increased following cotreatment with LATS-IN-1 and BAY1125976 compared with LATS-IN-1 alone. Right: decreased following cotreatment with LATS-IN-1 and BAY1125976 compared with LATS-IN-1 alone. (E) VEGFA and SHH in the conditioned medium measured using ELISA. Data are shown as mean ± SEM (n = 3 from 3 independent experiments). One-way ANOVA with Tukey’s multiple comparison test; ^∗^p < 0.05; ^∗∗^p < 0.01. (F and G) Live-cell imaging of FD-AOs treated with the compounds and quantification of organoid thickness and diameter. Scale bars, 100 μm. Data are shown as mean ± SEM (n = 60 from 3 independent experiments; 20 organoids were randomly selected from each experiment). One-way ANOVA with Dunnett’s multiple comparison test; ^∗∗∗^p < 0.005. (H) Immunofluorescence imaging of FD-AOs. Scale bars, 100 μm.

To further understand the effect of cotreatment with LATS-IN-1 and BAY1125976 on cell differentiation, we performed bulk RNA-seq analysis. Principal-component analysis (PCA) of the 16,430 genes showed that each compound-treated sample was divided into clusters (Figure 7A). In the PCA and Venn diagrams, BAY1125976-treated cells had a gene expression profile that was relatively close to that of the DMSO-treated control group, whereas each of the LATS-IN-1-treated and the two compound cotreated cells had distinct gene expression profiles that were far from the control group (Figures 7A and 7B). We next used the gene set of TRAVAGLINI_LUNG_ALVEOLAR_EPITHELIAL_TYPE1 (Travaglini et al., 2020) to comprehensively analyze the transcriptomes of each condition for elaborating the gene expression changes that characterize AT1 cells other than *AGER* (Figure 7C). LATS-IN-1 alone broadly increased the expression of AT1 cell marker genes, whereas cotreatment with LATS-IN-1 and BAY1125976 induced them more broadly and robustly. Next, we performed GO analysis to gain insight into how cotreatment with the compounds affected cellular biological processes compared to LATS-IN-1 treatment alone (Figure 7D). GOs related to AT1 cell characteristics, such as “vasculature development” and “response to decreased oxygen levels” and actin cytoskeleton, were ranked higher in the cotreated group. Since AT1 cells secrete multiple angiogenic factors and facilitate tissue formation during alveolar development (Zepp et al., 2021), angiogenic factors in the culture medium were measured (Figure 7E). Vascular endothelial growth factor A (VEGFA) was not synergistically increased following cotreatment with LATS-IN-1 and BAY1125976; however, sonic hedgehog (SHH) was predominantly elevated in the cotreated spheroids compared with the DMSO-treated control. The transcriptome of AT1 cells is enriched in genes associated with actin cytoskeleton regulation, suggesting that actin cytoskeleton determines AT1 cell morphology (Penkala et al., 2021; Shiraishi et al., 2023). We hypothesized that these reports and the GOs associated with the actin cytoskeleton could be the reminiscent of the thin-and-flat morphological changes characteristic of AT1 cells; however, it was impossible to measure the thickness and diameter of the spheroids in on-gel culture because LATS-IN-1 induced clumped morphological changes. Therefore, we analyzed the morphology in FD-AOs and found that LATS-IN-1 did not thin the organoids but rather thickened them, whereas BAY1125976 and cotreatment with LATS-IN-1 and BAY1125976 significantly thinned the organoids. In contrast, no significant change in organoid diameter was observed (Figures 7F and 7G). Alpelisib and AZD6482, PI3K/AKT inhibitors, consistently recapitulated the result of BAY1125976 (Figures S6A and S6B). Cotreatment with LATS-IN-1 and BAY1125976 increased AT1 marker-positive cells in FD-AOs (Figure 7H). These results suggest that the cotreatment with these two compounds reproduces not only the transcriptomic but also the functional and morphological features of iAT1 cells.

## Discussion

We identified the signaling pathways promoting AT2-to-AT1 cell differentiation by compound screening. Human iPSCs are a useful tool for studying lung alveolar differentiation, and we conceived a reporter cell-based screening method to determine signals that regulate AT2-to-AT1 cell differentiation. Fluorescent reporter genes help visualize differentiation, and HiBiT is superior to fluorescent reporters because it assesses changes in genes with low expression levels, such as *AGER*, sensitively and quantitatively. Many of the hit compounds, including BAY1125976, showed no apparent difference in mCherry fluorescence compared with the control group and would not have been detected without the HiBiT assay. In addition, we established a novel on-gel culture method of alveolar epithelial spheroids suitable for medium-throughput screening. On-gel culture is an epithelial cell-only culture system with advantages such as the ability to be cultured in 96-well plates, the small number of cells required per well, and the lack of complexity in embedding the cells in Matrigel. The on-gel culture enabled cell counting and the HiBiT assay to be performed in the same well. Furthermore, the spheroids were exposed on the outside of the gel, making the HiBiT substrate easily accessible to the spheroids without dissociation of the Matrigel. In the present study, multiple chemical compounds induced AT1 cell differentiation. Therefore, on-gel culture is beneficial for high-throughput screening or analyzing cell-based disease models using different markers in the future.

The chemical library used for screening contains several compounds that act on various intracellular signals, allowing for comprehensive evaluation of intracellular signaling. Since all of the compounds were used at 10 μM, false positives due to off-targeting remain a possibility. However, the library compensates for this concern since it contains multiple compounds per signal. Indeed, Alpelisib and AZD6482 did not reach hit criteria but increased HiBiT values (1.87- and 1.65-fold change vs DMSO, respectively), which helped identify the involvement of PI3K/AKT signaling in AT1 cell differentiation. Wnt signaling inhibitor and YAP/TAZ signaling activator were expected to be hits in the compound screening since we previously reported that XAV939, a suppressor of the canonical Wnt signaling via inhibition of tankyrase, promoted iAT1 cell differentiation from iAT2 cells in FD-AOs, and YAP/TAZ signaling is essential in AT1 cell differentiation (DiGiovanni et al., 2023; Gokey et al., 2021; Kanagaki et al., 2021; LaCanna et al., 2019; Liu et al., 2016; Nguyen et al., 2021; Warren et al., 2023). In the present study, we removed CHIR99021, which activates the canonical Wnt signaling via inhibition of GSK3β, from the medium before screening. Therefore, XAV939 was not a hit compound in this on-gel screening system without coculturing with fibroblasts. We speculated that fibroblasts could overactivate the Wnt pathway in AT2 cells in FD-AOs (Barkauskas et al., 2013; Kanagaki et al., 2021; Nabhan et al., 2018), whereas the canonical Wnt signaling was switched off by removing CHIR99021 from the medium during screening in an on-gel culture setting. XAV939 would have been a hit if the culture conditions had activated Wnt. LATS-IN-1 and ROCK-IN-2 showed the greatest increase in HiBiT luminescence among the hit compounds tested in this study, although ROCK-IN-2 appeared to stimulate YAP/TAZ signaling as an off-target effect. LATS-IN-1, which activates the YAP/TAZ signaling pathway, contributed significantly to AT1 differentiation, consistent with previous studies in mice and a recent study using human iPSC-derived AT2 cells (DiGiovanni et al., 2023; Gokey et al., 2021; LaCanna et al., 2019; Liu et al., 2016; Nguyen et al., 2021; Warren et al., 2023; Burgess et al., 2023). Our results provide insights into the relationship between PI3K/AKT signaling and AT1 differentiation. PI3K/AKT signal inhibition alone had a weak effect on the transcriptome, but when combined with LATS-IN-1, it significantly increased the expression of AT1 marker genes, suggesting that PI3K/AKT suppression under activated YAP/TAZ signaling is important. In this study, these two hit compounds promoted AT1 cell differentiation from CPM^hi^ progenitor cells as well as iAT2 cells. However, whether iAT2 cells transiently appear in the differentiation into AT1 cells from CPM^hi^ cells remains to be determined.

In the present study, treatment with LATS-IN-1 increased AT1 transcriptome broadly, but it alone did not thin the spheroids, consistent with recent *in vivo* reports that YAP/TAZ activation alone is insufficient for AT1 cell differentiation (Penkala et al., 2021). PI3K/AKT inhibitors induced thinning of the cells in FD-AOs, which is consistent with the recent report that AKT inhibition shortened stem cell length in the developing lung, indicating that AKT affects cell morphology in alveolar epithelial cells and is one of the factors controlling thinning (Liu et al., 2023). When dissociated AT2 cells were reseeded onto a 2D culture, the flattened morphology was achieved as well as increased AT1 cell markers (Kanagaki et al., 2021). Furthermore, activation of YAP/TAZ in the 2D culture spontaneously suppressed AKT (Figure 6D). We speculated that the change in the microenvironment of AT2 cells, such as the stiffness of the substrates or interactions with fibroblasts, was required for thinning during the AT1 cell differentiation along with the YAP/TAZ-related signal modulation. Nevertheless, the thinning of cells in the 3D culture conditions in the present study does not fully mimic the super-thin and super-flat AT1 morphology seen *in vivo*; hence, further modifications of the culture environment may better reproduce AT1 characteristics. In addition, because AKT can receive inputs from various signaling pathways other than KGF and YAP/TAZ signaling (Borok et al., 1998; Manning and Toker, 2017), AT1 differentiation may be regulated by some specific signals. Activated p53 signaling promotes AT1 cell differentiation, which is beneficial for suppressing lung adenocarcinoma (Kaiser et al., 2023). Activated p53 signaling induces PHLDA3, which represses AKT signaling and induces the apoptosis of cancer cells (Kawase et al., 2009), supporting our finding that AKT inhibition induced AT1 cells alternatively to activated p53 signaling and independently of YAP/TAZ signaling activation. Future studies are needed to elucidate the upstream role of AKT inhibition in AT1 cell differentiation.

In conclusion, we established a dual-reporter cell line for the quantitative evaluation of AT1 cell differentiation and a method of on-gel alveolar epithelial spheroid culture suitable for medium-throughput screening, identifying the activation of YAP/TAZ and inhibition of AKT as signals that promote AT1 differentiation. The findings of our study provide novel insight into human AT1 cell differentiation and contribute to lung regenerative medicine.

## Experimental procedures

### Resource availability

#### Lead contact

Further information and requests for resources and reagents should be directed to and will be fulfilled by the corresponding author, Shimpei Gotoh (gotoh.shimpei.5m@cira.kyoto-u.ac.jp).

#### Materials availability

Materials used in this study are available upon request under a completed materials transfer agreement.

#### Data and code availability

The accession number for the sequencing raw data reported in the present study is GEO: GSE241337.

### On-gel culture and compound treatment

Four types of flat-bottom clear plates were used for the on-gel culture: a 96-well white plate (Falcon 353377) for HiBiT assay, a 96-well black plate (PerkinElmer 6055300) for whole-mount immunofluorescence, a 48-well clear plate (Greiner 677180) for western blotting, and a 96-well clear plate (Greiner 655180) for the other experiments. For whole-mount immunofluorescence and western blotting, a plate was coated with 50% Matrigel diluted in the DCIK+3i (3 μM CHIR99021, 10 μM SB431542, and 10 μM Y-27632) medium in volumes of 25 and 100 μL, respectively. For other experiments, a 96-well plate was coated with 50 μL of Growth Factor Matrigel per well at least 30 min before seeding cells. A total of 5 × 10^3^ magnetic cell sorting (MACS)-isolated CPM^hi^ cells or 1 × 10^4^ FACS-isolated iAT2 cells were suspended in 100 μL DCIK+3i medium and seeded onto a Matrigel-coated 96-well plate. A total of 8 × 10^4^ MACS-isolated CPM^hi^ cells were suspended in 250 μL DCIK+3i medium and seeded onto a Matrigel-coated 48-well plate. Y-27632 was withdrawn after day 3, and the medium was changed every 3 days until day 12. When the compounds were evaluated, 2i (CHIR99021 and SB431542) were withdrawn from day 6 to exclude their effect on the results, and each compound was supplemented on day 9. The list of chemicals in the library is provided in Table S1. When the effect of KGF was evaluated, KGF was withdrawn from the DCIK medium on day 9. Each experiment was performed on day 12.

### HiBiT assay

Before the HiBiT assay, the cell count was measured using CCK8 (Dojindo CK04) according to the manufacturer’s instructions. The cells were incubated in the medium containing 10% CCK8 at 37°C for 1 h. Then, the medium was transferred into a new clear 96-well plate, and its absorbance was measured. After the cells were washed once with the medium, the medium was replaced with 100 μL fresh DCIK medium, and 100 μL of HiBiT reagent (Promega N3030) was added to each well. The plates were mixed on a shaker for 10 min and incubated at room temperature (RT) for 10 min. HiBiT luminescence was measured using an ARVO X5 plate reader (PerkinElmer), and luminescence values were corrected with the CCK8 absorbance values. When the HiBiT assay was performed in cell suspension, 3 × 10^4^ cells were suspended in 100 μL DCIK medium and mixed with 100 μL HiBiT reagent in a 1.5-mL tube. After vortexing and incubation at RT for 10 min, the samples were transferred to a white flat-bottom plate (Falcon 353296), and HiBiT luminescence was measured.

### Whole-mount immunofluorescent analysis of on-gel spheroids

Spheroids on-gel were washed with PBS and fixed with 4% paraformaldehyde in PBS for 1 h at RT. Spheroids attached to the bottom of the plate were gently washed in PBS three times, permeabilized, and immersed in a blocking buffer (5% BSA, 1% Triton X-100 in PBS) for 1 h. Primary antibodies were diluted in the blocking buffer, and spheroids were stained for 2 h at RT and washed with a wash buffer (1% Triton X-100 in PBS) four times. Secondary antibodies and Hoechst33342 (Dojindo 346-07951) were diluted in the blocking buffer, and spheroids were stained for 1 h at RT. After washing four times in the wash buffer, each sample was immersed in PBS. Immunofluorescent images were obtained using OperaPhenix (PerkinElmer). The antibodies that were used for immunofluorescence are listed in Table S1.

### VEGFA and SHH ELISA

The on-gel culture supernatant was collected and centrifuged at 200 × *g* for 10 min at 4°C. The supernatant was used for ELISA, which was performed according to the kit’s instructions (R&D Systems DVE00 and DSHH00). The number of cells on the plate from which the culture supernatant was removed was counted using CellTiter-Glo 2.0 Cell Viability Assay (Promega G9241), and the amount of VEGFA and SHH was corrected using the values of CellTiter-Glo.

### Statistical analysis and software

Data are represented as mean ± SEM. Statistical analysis was performed with GraphPad Prism 7, and p values <0.05 were considered significant. FlowJo version 10.6.1 (Becton Dickinson) was used to draw flow cytometry plots and their related analysis. The illustrations were created with Biorender.com.

## Supplemental information

Document S1. Figures S1–S6 and Tables S2–S5

Table S1. List of chemicals in the library used in this study

Document S2. Article plus supplemental information
