# Supplementary material for: Screening of factors inducing alveolar type 1 epithelial cells using human pluripotent stem cells
Source: Stem Cell Reports. 2024 Mar 28;19(4):529–44. doi: 10.1016/j.stemcr.2024.02.009 (PMC11096435; doi:10.1016/j.stemcr.2024.02.009)
Supplement: Document S1. Figures S1–S6 and Tables S2–S5 [file mmc1.pdf]

Stem Cell Reports, Volume *19*

Supplemental Information

Screening of factors inducing alveolar type 1 epithelial cells using human pluripotent stem cells

Yuko Ohnishi, Atsushi Masui, Takahiro Suezawa, Ryuta Mikawa, Toyohiro Hirai, Masatoshi Hagiwara, and Shimpei Gotoh

Supplemental information

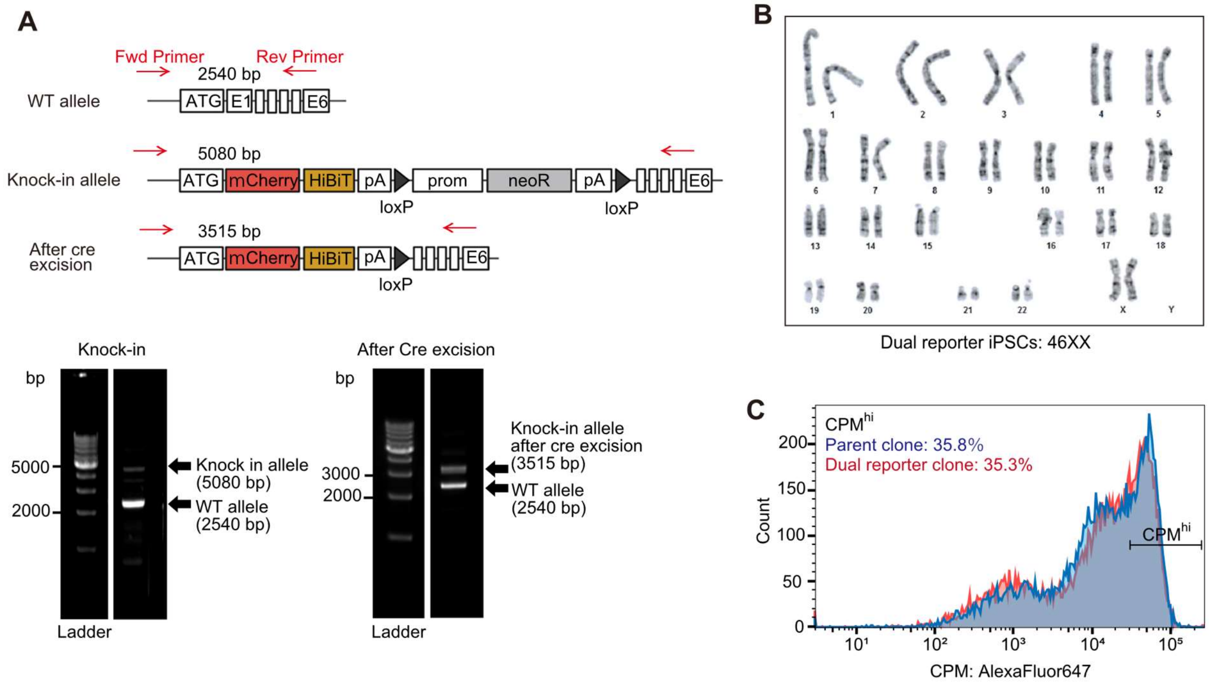

Figure S1. Validation of the *SFTPC*^{GFP} *AGER*$^{mCherry\text{-}HiBiT}$ dual-reporter iPSC line. Related to Figure 1.

A. Schematic diagram of PCR confirming the successful genome editing and the result of electrophoresis of PCR products. Red arrows represent the forward and reverse primers.
B. Karyotype of the dual reporter iPSC line (B2-3-dual-42-7).
C. Flow cytometry analysis of CPM^{hi} lung epithelial progenitor cells differentiated from the dual reporter iPSCs and its parental clone.

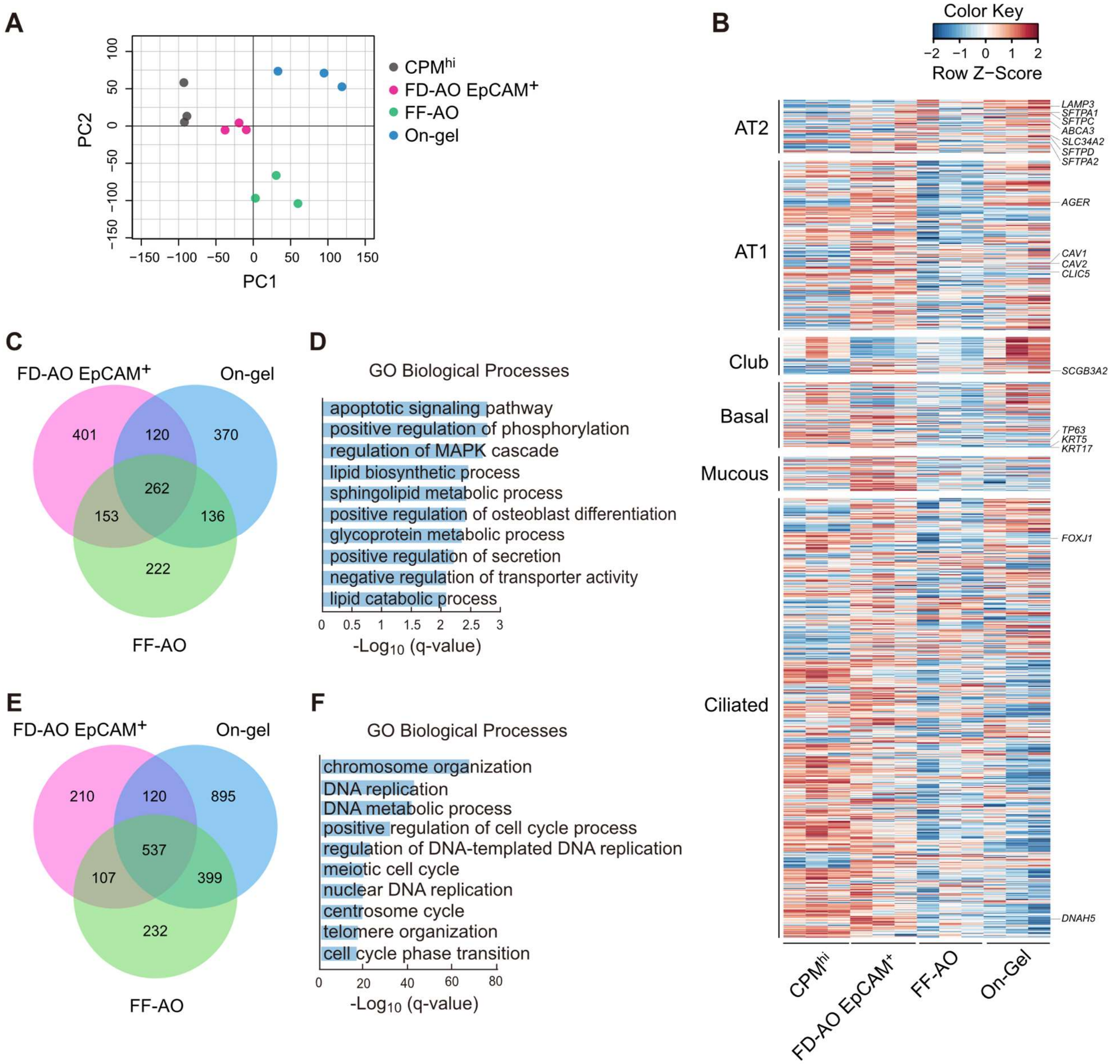

Figure S2. Characterization of the on-gel culture method. Related to Figure 2.

A. PCA of CPM^{hi} progenitor cells and P1 GFP^{+} iAT2 cell-derived epithelial cells using the three culture methods. Log_2 (TPM values) were used for analysis. Data were obtained from three independent experiments.

B. A heatmap of the transcriptomes of iPSC-derived lung epithelial cells was created with a value of log_2 (TPM + 0.01) using a gene set from the previous study[1].

C. Venn diagram of DEGs that were upregulated in epithelial cells from the three culture methods compared with CPM^{hi} cells. The threshold for upregulation was set to log_2 (fold-change) > 1, with an adjusted P-value of < 0.01.

D. Enrichment analysis of 370 genes specifically upregulated in on-gel alveolar epithelial spheroids.

E. Venn diagram of DEGs that were downregulated in epithelial cells from the three culture methods compared with CPM^{hi} cells. The threshold of downregulation was set to log_2 (fold change) < −1 with an adjusted P-value of < 0.01.

F. Enrichment analysis of 895 genes specifically downregulated in on-gel alveolar epithelial spheroids.

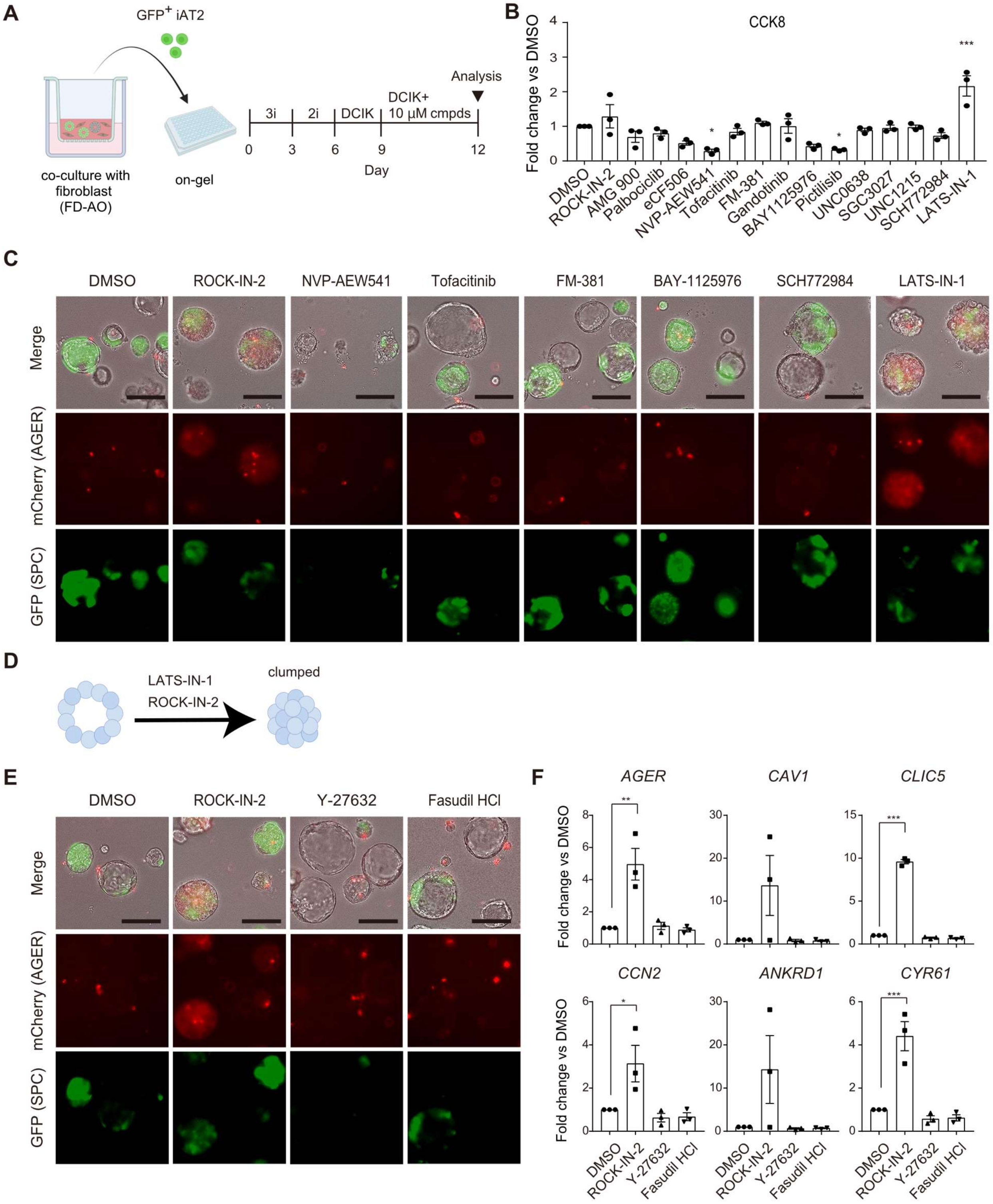
A
GFP+ iAT2
co-culture with fibroblast (FD-AO)
on-gel
3i
2i
DCIK
DCIK+ 10 μM cmpds
Analysis
0
3
6
9
12
Day
B
CCK8
Fold change vs DMSO
DMSO
ROCK-IN-2
AMG 900
Palbociclib
eCF506
NVP-AEW541
Tofacitinib
FM-381
Gandotinib
BAY1125976
Pictilisib
UNC0638
SGC3027
UNC1215
SCH772984
LATS-IN-1
C
DMSO
ROCK-IN-2
NVP-AEW541
Tofacitinib
FM-381
BAY-1125976
SCH772984
LATS-IN-1
Merge
mCherry (AGER)
GFP (SPC)
D
LATS-IN-1
ROCK-IN-2
clumped
E
DMSO
ROCK-IN-2
Y-27632
Fasudil HCl
Merge
mCherry (AGER)
GFP (SPC)
F
AGER
CAV1
CLIC5
CCN2
ANKRD1
CYR61
Fold change vs DMSO
DMSO
ROCK-IN-2
Y-27632
Fasudil HCl

Figure S3. Validation of screening hit compounds with GFP^+ iAT2 cell-derived spheroids on-gel. Related to Figure 3.

A. Schematic illustration of the strategy for the secondary evaluation of the hit compounds using GFP^+ iAT2 cell-derived on-gel alveolar epithelial spheroids.
B. CCK8 assay of on-gel iAT2 cell-derived spheroids treated with the compounds. Data are shown as mean ± SEM (n = 3 from independent experiments). One-way ANOVA with Dunnett's multiple comparison test: *P < 0.05, ***P < 0.005.
C. Live cell imaging of on-gel iAT2 cell-derived spheroids treated with each compound. Scale bars = 100 μm.
D. Illustration of the morphological change following treatment with LATS-IN-1 or ROCK-IN-2.
E. Live cell imaging of on-gel iAT2 cell-derived spheroids treated with each compound. Scale bars = 100 μm.
F. mRNA expression of GFP^+ iAT2 cell-derived spheroids treated with 10 μM of each ROCK inhibitor. Data are shown as mean ± SEM (n = 3 from independent experiments). One-way ANOVA with Dunnett's multiple comparison test: *P < 0.05, **P < 0.01, ***P < 0.005.

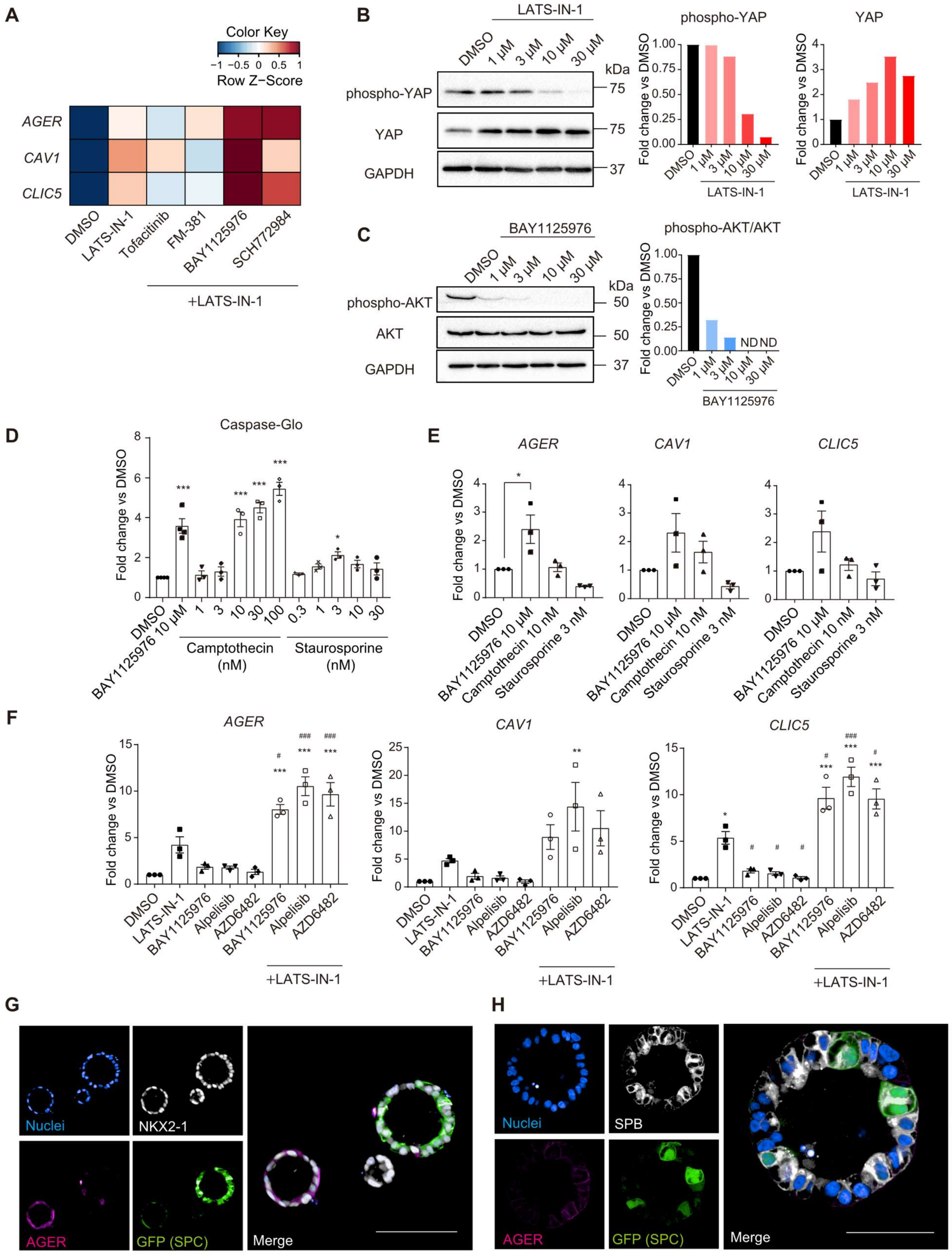
A
Color Key
−1 −0.5 0 0.5 1
Row Z−Score
AGER
CAV1
CLIC5
DMSO
LATS-IN-1
Tofacitinib
FM-381
BAY1125976
SCH772984
+LATS-IN-1
B
LATS-IN-1
DMSO
1 μM
3 μM
10 μM
30 μM
kDa
phospho-YAP
YAP
GAPDH
75
75
37
phospho-YAP
YAP
Fold change vs DMSO
C
BAY1125976
phospho-AKT
AKT
GAPDH
50
50
37
phospho-AKT/AKT
ND ND
D
Caspase-Glo
Fold change vs DMSO
BAY1125976 10 μM
Camptothecin (nM)
Staurosporine (nM)
E
AGER
CAV1
CLIC5
Camptothecin 10 nM
Staurosporine 3 nM
F
AGER
CAV1
CLIC5
Alpelisib
AZD6482
+LATS-IN-1
G
Nuclei
NKX2-1
AGER
GFP (SPC)
Merge
H
Nuclei
SPB
AGER
GFP (SPC)
Merge

Figure S4. PI3K/AKT signaling involvement in AT1 cell differentiation. Related to Figure 4.

A. AT1 cell marker gene expression in the on-gel alveolar epithelial spheroids using GFP⁺ iAT2 cells derived from dual-reporter iPSCs (P1). On-gel spheroids were treated with 10 μM of each compound. Data are shown as the mean of the $\log_2$ (ratio to adult lung) (n = 3 from independent experiments).

B. Western blotting of the on-gel alveolar epithelial spheroids using *SFTPC*^{GFP} reporter iPSC-derived CPM^{hi} lung progenitor cells. Spheroids were treated with LATS-IN-1. Phospho (S127) -YAP and total YAP bands were quantified and normalized by the levels of GAPDH.

C. Western blotting of the on-gel alveolar epithelial spheroids using *SFTPC*^{GFP} reporter iPSC-derived CPM^{hi} lung progenitor cells. Spheroids were treated with BAY1125976. Phospho (Ser473) -AKT and total AKT bands were quantified and phosphor-AKT bands were normalized to total AKT bands. ND, Not Detected.

D. Caspase-Glo assay to quantify the apoptosis of GFP⁺ iAT2 cells derived from *SFTPC*^{GFP} *AGER*$^{mCherry\text{-}HiBiT}$ dual-reporter iPSCs (P1) affected by BAY1125976 and different concentrations of apoptosis inducers, Camptothecin and Staurosporine. Data are shown as mean ± SEM (n = 3 from independent experiments). One-way ANOVA with Dunnett's multiple comparison test: *P < 0.05, ***P < 0.005.

E. AT1 cell marker gene expression in the on-gel alveolar spheroids using GFP⁺ iAT2 cells derived from the dual-reporter iPSCs (P1). Each compound was applied at arbitrary concentrations. Data are shown as mean ± SEM (n = 3 from independent experiments). One-way ANOVA with Dunnett's multiple comparison test: *P < 0.05, ***P < 0.005.

F. AT1 cell marker gene expression in the dual-reporter GFP⁺ iAT2 cell-derived on-gel spheroids (P1). Each column represents samples treated with 10 μM of each compound alone or a combination of PI3K/AKT inhibitors and LATS-IN-1. Data are shown as mean ± SEM (n = 3 from independent experiments). One-way ANOVA with Tukey's multiple comparison test was used for analysis. Results are shown only in comparison with the DMSO-treated group: *P < 0.05, **P < 0.01, ***P < 0.005, and the LATS-IN-1-treated group: #P < 0.05, ###P < 0.005.

G, H. Whole mount immunofluorescence imaging of on-gel spheroids derived from GFP⁺ iAT2 using *SFTPC*^{GFP} reporter iPSCs treated with DMSO. Scale bars = 100 and 50 μm respectively.

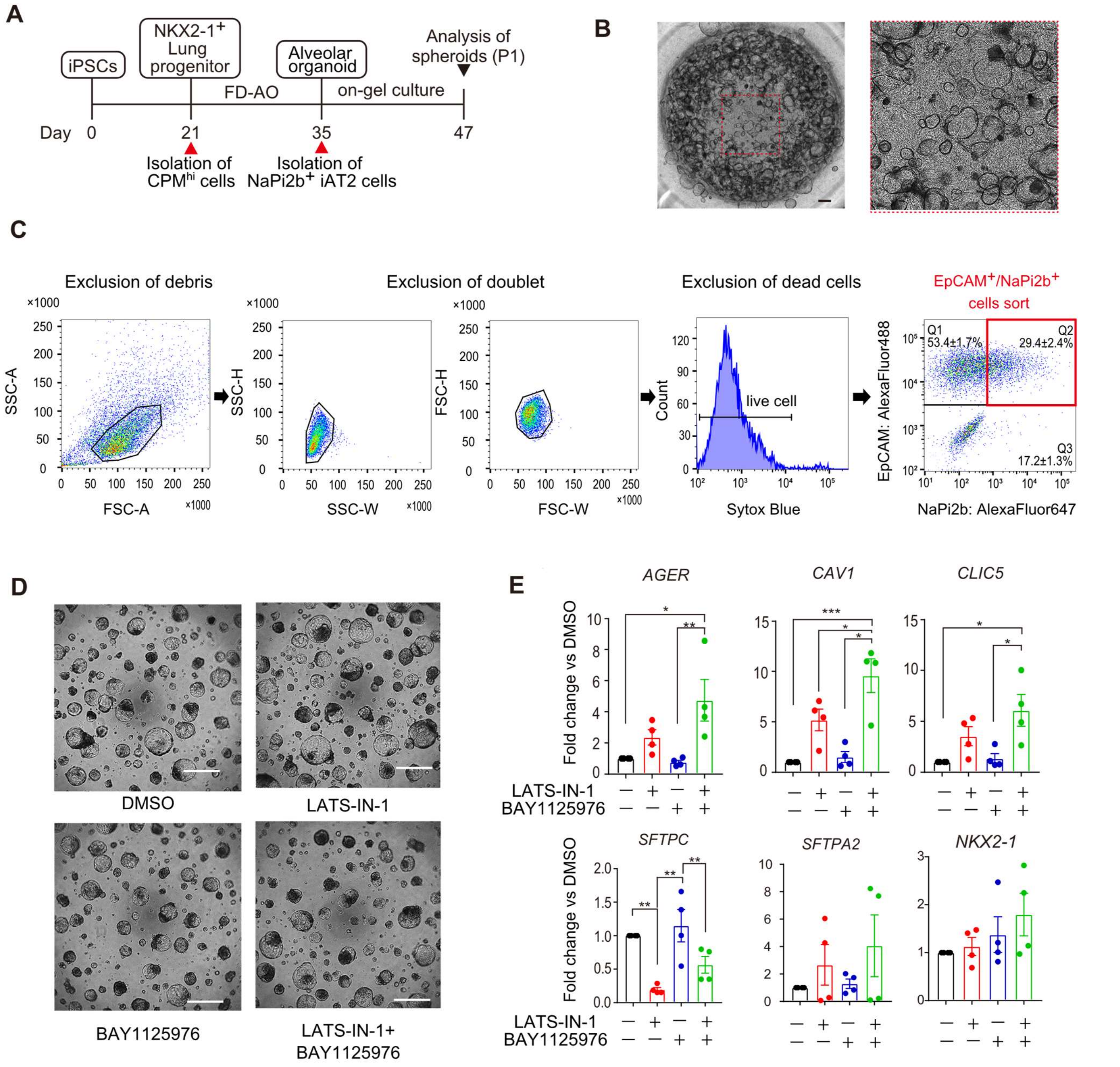

Figure S5. Validation of synergistic effect of YAP/TAZ activation and AKT suppression using ChiPSC18 line. Related to Figure 4.

A. Schematic diagram of the differentiation of the ChiPSC18 line into iAT2 cells followed by on-gel culture.
B. Live-cell imaging of FD-AOs derived from ChiPSC18 line. Scale bar = 500 µm.
C. Flow cytometry gating strategy for isolating iAT2 cells from FD-AOs derived from ChiPSC18 cells. The rate of each gated cell is shown as mean ± SEM (n = 3 from independent experiments).
D. Live-cell imaging of the compound-treated on-gel spheroids derived from iAT2 cells. Scale bars = 500 µm.
E. mRNA expression in on-gel iAT2 cell-derived spheroids (P1). Each column represents samples treated with 10 µM LATS-IN-1, 10 µM BAY1125976, or both. Data are shown as mean ± SEM (n = 4 from independent experiments). One-way ANOVA with Tukey's multiple comparisons test: *P < 0.05, **P < 0.01, ***P < 0.005.

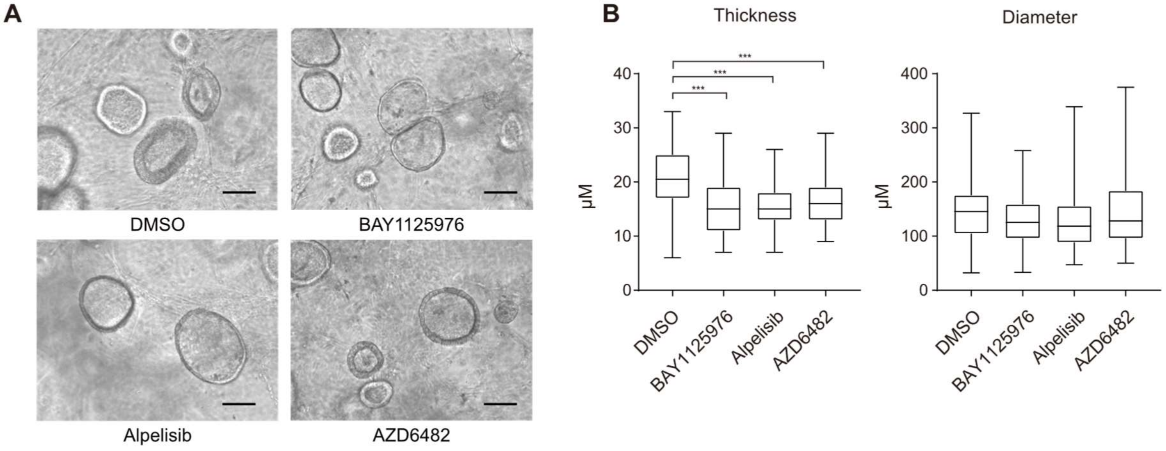

Figure S6. Morphological changes of FD-AOs induced by PI3K/AKT signaling inhibitors. Related to Figure 7.

A. Live cell imaging of FD-AOs (P2) treated with the indicated compounds. Scale bars = 100 μm.
B. Quantification of the organoid thickness and diameter. Data are shown as mean ± SEM (n = 60 from three independent experiments. A total of 20 organoids were randomly selected from each experiment). One-way ANOVA with Dunnett's multiple comparison test: ***P < 0.005.

Table S2: List of antibodies used in this study

Epitope	Host	Supplier	Catalog No.	Dilution (IF)	Dilution (FACS, MACS)	Dilution (Immunoblot)
HT2-280	Mouse	Terrace Biotech	SKU: TB-27AHT2-280		1:100	
NaPi2b	Mouse	kindly provided by Dr. Gerd Ritter (MX35)		1:100	1:100	
mCherry	Chicken	Abcam	ab205402	1:500		
GFP	Chicken	Abes labs	GFP -1020	1:500		
GFP	Rabbit	Cell Signaling Technology	2956	1:200		
EpCAM	Goat	R&D systems	AF960		1:100	
EpCAM	Mouse	Santa Cruz Biotechnology	sc-66020		1:100	
EpCAM-APC	Mouse	Milteny Biotec	130-113-260		1:100	
AGER	Goat	R&D systems	AF1145	1:100		
HT1-56	Mouse	Terrace Biotech	SKU: TB-29AHT1-56	1:150		
CPM	Mouse	Fujifilm Wako	014-27501		1:100	
SPB	Mouse	Santa Cruz	133143	1:50		
NKX2-1	Rabbit	Abcam	76013	1:100		
Phospho-YAP	Rabbit	Cell Signaling Technology	13008			1:1000
YAP	Mouse	Santa Cruz	101199			1:400
Phospho-AKT	Rabbit	Cell Signaling Technology	4060			1:1000
AKT	Rabbit	Cell Signaling Technology	9272			1:1000
GAPDH-HRP	Mouse	Fujifilm Wako	015-25473			1:3000
Chicken IgY-Cy3	Donkey	Jackson laboratory	703-165-155	1:500		
Chicken IgY-Alexa Fluor 488	Donkey	Jackson laboratory	703 -485-155	1:500		
Goat IgG-Alexa Fluor 488	Donkey	ThermoFisher Scientific	A11055		1:100	
Goat IgG-Alexa Fluor 647	Donkey	ThermoFisher Scientific	A21447	1:500		
Mouse IgM-Alexa Fluor 647	Donkey	Jackson laboratory	715-605-140		1:100	

Mouse IgG-Alexa Fluor 647	Donkey	ThermoFisher Scientific	A31571		1:100	
Mouse IgG-Alexa Fluor 488	Donkey	ThermoFisher Scientific	A21202	1:500		
Mouse IgG-Alexa Fluor 546	Donkey	ThermoFisher Scientific	A10036	1:500		
Rabbit IgG-Alexa Fluor 488	Donkey	ThermoFisher Scientific	A21206	1:500		
Mouse IgG-HRP	Horse	Cell Signaling Technology	7076			1:5000
Rabbit IgG-HRP	Horse	Cell Signaling Technology	7074			1:5000

Table S3. Primers used for TaqMan qPT-PCR

Gene name	TaqMan ID
18S	Hs99999901_s1
AGER	Hs00542584_g1
SFTPC	Hs00161628_m1

Table S4. Primers used for SYBR qPT-PCR

Gene name	Forward primer	Reverse primer
18S	TTGACGGAAGGGCACCACCAG	GCACCACCACCCACGGAATCG
CAV1	AGGGCAACATCTACAAGCCC	GCCGTCAAAACTGTGTGTCC
CLIC5	CTATGATATCCCGGCTGAGATGAC	CACGGGCATAGGCGTTCTT
GPRC5A	AGACAGGGGACACGCTCTAT	GGAGGCAAACTGTTCCCGTA
NKX2-1	AGCACACGACTCCGTTCTC	GCCCACTTTCTTGTAGCTTTCC
mCherry	AAGGCCTACGTGAAGCACCC	ATCACGCGCTCCCACTTGAA
SFTPA2	AAGCAGCTGGAGGCTCTGT	CCATCAAGATGAGGGTGAGG
LAMP3	ACCGATGTCCAACTTCAAGC	TGACACCTTAGGCGGATTTT

Table S5. Compounds related to Figure 3C, S3, S4

Compound	Cat.	Supplier
ROCK-IN-2	S6636	Selleck Biotech
AMG 900	HY-13253	MedChemExpress
Palbociclib	HY-50767A	MedChemExpress
eCF506	HY-112096	MedChemExpress
NVP-AEW541	HY-50866	MedChemExpress
Tofacitinib	S2789	Selleck Biotech
FM-381	HY-102046	MedChemExpress
Gandotinib	HY-13034	MedChemExpress
BAY-1125976	HY-100018	MedChemExpress

Pictilisib	HY-50094	MedChemExpress
UNC0638	HY-15273	MedChemExpress
SGC3027	HY-112445	MedChemExpress
UNC1215	13968	Cayman CHEMICAL
SCH772984	HY-50846	MedChemExpress
LATS-IN-1	E1061	Selleck Biotech
Fasudil HCl	S1573	Selleck Biotech
Y-27632	HY-10071	MedChemExpress
Alpelisib	HY-15244	MedChemExpress
AZD6482	HY-10344	MedChemExpress
Camptothecin	038-18191	Fujifilm Wako
Staurosporine	197-10251	Fujifilm Wako

SUPPLEMENTARY METHODS

Generation of *SFTPC*^{GFP} *AGER*$^{mCherry\text{-}HiBiT}$ dual-reporter iPSC

A guide RNA (gRNA) was designed to cleave downstream of the *AGER* start codon (gRNA sequence: 5`-ATGGCAGCCGGAACAGCAGT-3`). gRNA was synthesized (Macrogen) and inserted into pHL-H1-ccdB-mEF1a-RIH, which was a gift from Dr. Akitsu Hotta (Addgene plasmid # 6060). A donor vector carrying the *mCherry-HiBiT* reporter gene was designed and synthesized (Vector Builder). Before genome editing, the *SFTPC*^{GFP} reporter iPSC line (B2-3) was maintained on an iMatrix-511 (Takara Bio T311)-coated plate in StemFit AK02N medium (Ajinomoto AK02N) supplemented with penicillin-streptomycin (P–S; Thermo Fisher Scientific 15140122). Cells were treated with Y-27632 (LC Laboratories) at 10 μM for at least 1 h before electroporation. After washing cells with PBS, cells were dissociated into single cells with Accutase (Innovative Cell Technologies ICT-AT104-500-500) for 20 min under 37°C and collected with StemFit AK02N supplemented with 10 μM Y-27632. 5 μg of sgRNA vector, 5 μg of donor vector, and 5 μg of pHL-EF1a-SphcCas9-iP-A, a gift from Dr. Akitsu Hotta (Addgene plasmid # 60599) (Li et al., 2015), were electroporated into 1×10^6 cells suspended in Opti-MEM (ThermoFisher Scientific 31985062) using NEPA21 electroporator (Nepa Gene). The cells were re-seeded on an iMatrix-511-coated plate in StemFit AK02N supplemented with 10 μM Y-27632. 2 days after electroporation, Y-27632 was removed. 6 days after electroporation, the cells were cultured with StemFit AK02N supplemented with 100 μg/mL G418 (Gibco 10131035) for 6 days to select the transfected cells. Cells were dissociated and re-seeded at limiting dilutions of 200, 500, and 1,500 cells in a 10 cm dish coated with iMatrix-511 in StemFit AK02N and cultured for 7-10 days. Single clones were screened using genomic PCR and Sanger sequencing to obtain positive clones with the knock-in of the reporter gene into a single allele. A total of 10 μg of pCXW-Cre-Puro (a kind gift from Dr. Keisuke Okita) was electroporated into two positive clones, and the cells were re-seeded at limiting dilutions. Single clones were checked for excision of the G418 resistance gene cassette by genomic PCR and Sanger sequencing, and a dual-reporter iPSC clone (B2-3-dual-42-7) was selected for subsequent experiments. Karyotyping of the dual-reporter iPSC (B2-3-dual-42-7) was performed using G-banding (Nihon Gene Research Laboratories Inc, Japan).

Culture of human iPSC and fetal lung fibroblasts

Human iPSCs were cultured, as described previously (Yamamoto et al., 2017). Briefly, the *SFTPC*^{GFP} reporter iPSC line (B2-3), *SFTPC*^{GFP} *AGER*$^{mCherry\text{-}HiBiT}$ dual-reporter iPSC line (B2-3-dual-42-7), and ChiPSC18 (Takara-bio Y00305) cell line were cultured on Geltrex (Thermo Fisher Scientific A1413202)-coated plates in Essential 8 medium (Thermo Fisher Scientific A1517001) without feeder cells. Human fetal lung fibroblasts (HFLF) (17.5 weeks of gestation; DV Biologics #PP002-F-1349, lot 121109VA) were cultured in Dulbecco's Modified Eagle's medium (Nacalai Tesque 08459-64) supplemented with 50 U/mL P–S and 10% fetal bovine serum (Sigma Aldrich SF7524).

Differentiation of iPSC into NKX2.1+ lung epithelial progenitor cells

Human iPSCs were differentiated into lung epithelial progenitor cells as previously describe[2]. Briefly, each iPSC clone was seeded on a Geltrex-coated plate and cultured in RPMI medium (Nacalai Tesque 30264-56)

supplemented with Activin A (API GF-001-050L), 1 μM CHIR99021 (Axon Medchem), 10 μM Y-27632, 2% B-27 supplement (Thermo Fisher Scientific 17504001), and 50 U/mL P–S. One day after seeding, sodium butyrate was added at a final concentration of 0.25 mM. From day 2 to 5, the cells were cultured in RPMI medium supplemented with activin, 1 μM CHIR99021, 0.125 mM sodium butyrate, 2% B-27 supplement, and 50 U/mL P–S. After day 6, the cell culture medium was formulated based on DMEM/F12 (Thermo Fisher Scientific 11320033) supplemented with Glutamax (Thermo Fisher Scientific 35050-061), 2% B27 supplement, 0.05 mg/mL L-ascorbic acid (Wako 016-04805), 0.4 mM monothioglycerol (Wako 195-15791), and 50 U/mL P–S. From day 6 to 10, the cells were cultured in a basal medium containing 100 ng/mL Noggin (R&D systems 6057-NG-01M) and 10 μM SB431542 (Fujifilm Wako 198-16543) to differentiate into anterior foregut endoderm (AFE) cells. From the day 10 to 14, the medium was changed to basal medium supplemented with 3 μM CHIR99021, 0.05 μM all-trans retinoic acid (Sigma-Aldrich R2625), and 20 ng/mL BMP4 (Peprotech 120-05ET) for B2-3 and B2-3-dual-42-7 iPSC line, 3.5 μM CHIR99021, 1 μM all-trans retinoic acid, and 20 ng/mL BMP4 for ChiPSC18 line for differentiation into ventralized anterior foregut endoderm (VAFE) cells. From day 14 onward, cells were cultured in the basal medium supplemented with 3 μM CHIR99021, 10 ng/mL FGF10 (PeproTech 100-26), 10 ng/mL KGF (PeproTech AF-100-19), and 20 μM DAPT (Fujifilm Wako 049-33583) to differentiate into $NKX2.1^{+}$ lung progenitor cells. On day 21, CPM^{hi} cells were labeled with mouse anti-human CPM antibody (Fujifilm Wako 014-27501) and isolated by FACS using AlexaFluor647-conjugated donkey anti-mouse IgG antibody (Thermo Fisher Scientific A31571) or by magnetic cell sorting (MACS) using anti-mouse IgG MicroBeads (Miltenyi Biotec 130-047-302).

Induction and passages of iAT2 cells in FD-AOs

The isolated CPM^{hi} lung epithelial progenitor cells were differentiated into iAT2 cells in the FD-AOs in a 12-well plate with DCIK medium: Ham's F12 (Fujifilm Wako, 087-08335) containing 50 nM dexamethasone (Sigma-Aldrich, D4902), 100 μM 8-Br-cAMP (Biolog Life Science Institute, B007), 100 μM 3-isobutyl-1-methylxanthine (Fujifilm Wako, 099-03411), 10 ng/mL KGF, 1% B-27 supplement, 0.25% bovine albumin fraction V (Thermo Fisher Scientific,15260-037), 15 mM HEPES (Thermo Fisher Scientific, 17557-94), 0.8 mM $CaCl_2$ (Fujifilm Wako, 036-19731), 0.1% ITS premix (Corning, 354352), 50 U/mL Penicillin/Streptomycin, as described previously[2]. Briefly, 1×10^4 CPM^{hi} cells and 5×10^5 HFLF were mixed in 200 μL of 50%Growth Factor Reduced Matrigel (Corning 354230) diluted with DCIK+10 μM Y-27632 medium and seeded on a cell-culture insert. A 1 mL of DCIK medium was added to the lower chamber and supplemented with 10 μM Y-27632 for the first 3 days. The medium was changed every 2–3 days for two weeks. $SFTPC^{GFP}$ reporter iPSC-derived iAT2 cells were isolated by gating $EpCAM^{+}/GFP^{+}$ cells, whereas ChiPSC18 iPSC-derived iAT2 cells were isolated by gating $EpCAM^{+}/NaPi2b^{+}$ cells using FACS as described previously [3]. Isolated iAT2 cells were reseeded for on-gel culture.

Isolation of epithelial cells from FD-AOs

The matrices of FD-AOs were collected in 15 mL tubes containing 0.1% Trypsin EDTA (Thermo Fisher Scientific 25200072), and spheroids were dissociated into single cells via pipetting. After neutralizing trypsin by adding twice the volume of Dulbecco's Modified Eagle's medium supplemented with 50 U/mL P–S and 10% fetal bovine serum, the cells were collected via centrifugation at 4 °C, 900 rpm for 5 min. $EpCAM^{+}$ epithelial cells were isolated by MACS using a mouse anti-human EpCAM antibody (Santa Cruz SC-66020) and anti-mouse IgG MicroBeads (Table S2).

Flow cytometry (FCM)

"On-gel" spheroids were incubated with cell recovery solution (Corning 354253) for 1 h at 4 °C. The spheroids were collected and dissociated into single cells using Accutase for 20 min. The cells were suspended in FCM buffer (1% BSA and 10 μM Y-27632 in PBS) containing SYTOX Blue Dead Cell Stain (Thermo Fisher Scientific S34857) and analyzed using FACS Aria III (BD Biosciences).

Live cell imaging and measurement of organoid morphology

Live-cell images of organoids were acquired using a BZ-X710 microscope (Keyence). The thickness of the epithelium and the diameter of the spheroids were measured in 20 randomly selected spheroids in each sample per test using the BZ-X Analyzer.

Transmission electron microscopy

As described previously (Gotoh et al., 2014), each sample was fixed in 0.1M phosphate buffer (pH 7.4) containing 0.1% picric acid, 2% paraformaldehyde, 2% osmium tetroxide, 2.5% glutaraldehyde, and 4% sucrose. En-bloc

staining was performed using 1% uranyl acetate solution. The specimens were dehydrated and embedded in Epon 812 (Nacalai Tesque). The ultrathin sections were stained with uranyl acetate and lead citrate. Transmission electron microscopy (Nihondenshi, JEM-1400Flash) was used for the examination.

mRNA expression analysis

Total RNA was extracted using the RNeasy Micro Kit (QIAGEN 74004) or RNeasy Mini Kit (QIAGEN 74106), according to the manufacturer's protocol. ReverTra Ace qPCR RT Master Mix with gDNA Remover (Toyobo FSQ-301) was used for the reverse transcription of RNA. Quantification was performed using THUNDERBIRD Probe qPCR Mix (Toyobo QPS101) or Power SYBR Green Master Mix (Thermo Fisher Scientific 4368706); and the primers used are listed in Tables S3 and S4. Gene expression was normalized to that of 18S rRNA. The expression of each gene was compared with that of the human adult lung five donor pool (BioChain #R1234152-P, lot A811037) or each control.

RNA-seq and bioinformatics analysis

RNA was extracted using the RNeasy Mini Kit or RNeasy Micro Kit, and its quality was checked using the Agilent RNA 6000 Pico Assay Kit (Agilent Technologies) according to the manufacturer's protocol. The sequence library was prepared using Illumina Stranded Total RNA Prep, ligation with Ribo-Zero Plus (Illumina), and the quality of the library was checked using a High Sensitivity D500 kit and 4200 TapeStation (Agilent). The sequence was performed using Illumina NextSeq2000. FASTQ files were created using bcl2fastq-2.20. Cut adapt ver v4.1 was used for trimming adaptor sequences and low-quality bases from the raw reads. The trimmed reads were mapped to the human reference genome sequence (hg38) using STAR version 2.7.10a and a GENCODE (GRCh38.p13, release 32) GTF file. Htseq-count ver. 2.0.2 was used for calculating the row counts with the GENCODE GTF file. Gene expression levels were calculated as transcripts per kilobase million (TPM) with DEseq2 v1.34.0. Low expression genes with average TPM values among the comparison data set of < 1 were excluded for the following analyses. The R package "maptools" was used for describing the principal component analysis (PCA) of $\log_2$ (TPM + 0.01). The R package DESeq2 (Love et al., 2014) was used to identify the DEGs between the two groups. The R package "gplots" was used for creating a heatmap based on $\log_2$ (TPM + 0.01). Venn diagram was created with the R package "VennDiagram". Enrichment analysis for gene ontology (GO) Biological Processes was analyzed using the Metascape online software, and the analysis was limited to a "summary" group of GO.

Immunofluorescence analysis

Each sample was fixed with 4% paraformaldehyde in PBS. After being washed in PBS, each sample was immersed in 30% sucrose in PBS, embedded in OCT compound (Sakura Finetek 45833), and frozen at -80°C. The sample blocks were sliced into 10 μm with cryotome (Leica), permeabilized in 0.2% Triton X-100, immersed in the blocking buffer (5% Donkey serum, 1% bovine serum albumin (BSA) (Sigma A9647) in PBS, and stained, as described previously [4]. Immunofluorescence images were obtained using a confocal microscope (Leica SP8).

Caspase-Glo 3/7 assay

Prior to the Caspase-Glo assay, the cell count was measured using CCK8, according to the manufacturer's instructions. The cells were incubated in the medium containing 10% CCK8 at 37°C for 1 h. Then, the medium was transferred into a new clear 96-well plate and its absorbance was measured. After the cells were washed once with the medium, the medium was replaced with 50 μL of fresh DCIK medium, and 50 μL of Caspase-Glo 3/7 reagent (Promega N8090) was added to each well. The plates were mixed on a shaker for 30 sec and incubated at room temperature for 30 min. Luminescence was measured using ARVO X5 plate reader (PerkinElmer), and luminescence values were corrected with the CCK8 absorbance values.

Two-dimensional (2D) culture

A 6-well cell culture plate was coated with an iMatrix-511 at the concentration of 0.5 μg/cm^2 for at least 1 hour under 37°C before cell seeding; 1×10^6 of MACS-sorted CPM^{hi} progenitor cells were diluted in the 2 mL of DCIK+10 μM Y-27632 medium and seeded on iMatrix-511 coated plate. Y-27632 was withdrawn from the medium on day 4. LATS-IN-1 was applied at 10 μM final concentration on day 4 and cells were harvested on day 7.

Western blotting

Spheroids cultured in on-gel or 2D cultured cells were washed with PBS and harvested with Matrigel using 2×Lysis buffer consisting of 2×RIPA buffer (Nacalai Tesque 08714-04), 2% SDS (Biorad 1610301), and 2×Phosphatase

Inhibitor Cocktail (Nacalai Tesque 07574-61). Cell extracts were centrifuged at 4 °C and 15,000 × g for 20 min. Each supernatant was collected, and protein concentration was measured using a BCA protein assay kit (Thermo Fisher Scientific 23227). Protein concentrations were matched between samples, and 4xLDS sample buffer (Thermo Fisher Scientific NP0007) supplemented with 2-mercaptoethanol was added to each sample and incubated at 70 °C for 10 min. Samples were loaded on 8% polyacrylamide gel, electrophoresed in Tris-Glycine SDS buffer (Biorad1610732), and transferred to PVDF membrane (0.2 μm Merck) in Tris-Glycine buffer (Biorad 1610734) supplemented with 20% methanol. The membrane was blocked with 50% Blocking One-P (Nacalai Tesque 05999-84) diluted with TBS-T (Takarabio T9142) for 30 min and incubated with primary antibodies (Table S2) diluted with Can Get Signal Immunoreaction Enhancer Solution 1 (TOYOBO NKB-101) at 4 °C overnight. On the following day, the membrane was washed with TBS-T three times and incubated with secondary antibodies diluted with Can Get Signal Immunoreaction Enhancer Solution 2 (TOYOBO NKB-301) for 1 h at room temperature and washed with TBS-T three times. Bands were detected using Pierce ELC plus Western Blotting Substrate (Thermo Fisher Scientific 32132) and images were acquired using ChemiDoc Touch MP. Image Lab software 6.1 was used to quantify the bands.

Primary adult human alveolar epithelial cell culture

Adult human alveolar epithelial cells were purchased from ScienCell Research Laboratories (cat. # 3240). To sustain and expand cells, 2-3×10^4 cells were mixed with 50 μL of Growth Factor Reduced Matrigel and generated a Matrigel dome; 2-3 domes were generated per well of a 6-well plate. After incubation at 37 °C for > 30 min, 2 mL of PneumaCult™ Alveolar Organoid Expansion Medium (AvOE) containing Passage Supplement (STEMCELL Technologies ST-100-0847) was added per well. The medium was changed to AvOE without Passage Supplement from day 3, and the medium was changed every 2-3 days, until day 14. On day 14, Matrigel domes were collected in 15 mL tubes containing 0.1% Trypsin EDTA, and spheroids were dissociated into single cells via pipetting. After neutralizing trypsin by adding twice the volume of Dulbecco's Modified Eagle's medium supplemented with 50 U/mL P–S and 10% fetal bovine serum, the cells were collected via centrifugation at 4 °C, 900 rpm for 5 min. Dissociated cells were stained with 1 μL of anti-HT2-280 antibody per million cells for 20 min on ice, and subsequently stained with AlexaFluor647-conjugated anti-mouse IgM for 20 min on ice. HT2-280 positive cells were isolated using FACS and used for subsequent experiments or passages. When compounds were tested, 1×10^4 primary human AT2 cells were seeded for on-gel culture with Advanced DMEM (Thermo Fisher Scientific 12634010) supplemented with 2% B-27 supplement, 0.5% P–S, 10 mM HEPES (Nacalai Tesque 17557-94), 1×Glutamax (Gibco 35050-061), 1 mM N-acetyl-L-Cysteine (Sigma Aldrich SLCB5087), 50 ng/mL EGF (Thermo Fisher Scientific PHG0314), 5 ng/mL KGF , 100 ng/mL FGF-basic (Peprotech PEP-100-18B), 100 ng/mL FGF10, 100 ng/mL IGF1 (Biolegend 590906), 100 ng/mL Neuregulin1 (Peptotech PEP-100-03), 100 ng/mL Noggin, 10 nM [Leu15]Gastrin1 (Sigma Aldrich G914), 500 nM A83-01 (Wako 039-24111), 20 nM R-spondin-1 (Peprotech 120-38), 0.2% Afamin-Wnt-3A serum-free conditioned medium (MBL Life Science J2-001), and 10 μM Y-27632 described previously[5]. Y-27632 was withdrawn after day 3. From day 6, A83-01, R-spondin-1, and Afamin-Wnt-3A serum-free conditioned medium were withdrawn. On day 9, the compounds were added to the medium for 3 days, and subsequent experiments were performed.

Ethics

All iPSC cell lines (B2-3, B2-3-dual-42-7, and ChiPSC18), primary AT2 cells and fetal lung fibroblasts were exempt from ethical approval.

SUPPLEMENTAL REFERENCES

1. Travaglini, K.J., Nabhan, A.N., Penland, L., Sinha, R., Gillich, A., Sit, R.V., Chang, S., Conley, S.D., Mori, Y., Seita, J., et al. (2020). A molecular cell atlas of the human lung from single-cell RNA sequencing. Nature *587*, 619–625.

2. Yamamoto, Y., Gotoh, S., Korogi, Y., Seki, M., Konishi, S., Ikeo, S., Sone, N., Nagasaki, T., Matsumoto, H., Muro, S., et al. (2017). Long-term expansion of alveolar stem cells derived from human iPS cells in organoids. Nat. Methods *14*, 1097–1106.

3. Korogi, Y., Gotoh, S., Ikeo, S., Yamamoto, Y., Sone, N., Tamai, K., Konishi, S., Nagasaki, T., Matsumoto, H., Ito, I., et al. (2019). In Vitro Disease Modeling of Hermansky-Pudlak Syndrome Type 2 Us- ing Human Induced Pluripotent Stem Cell-Derived Alveolar Organoids. Stem Cell Reports.

4. Suezawa, T., Kanagaki, S., Moriguchi, K., Masui, A., Nakao, K., Toyomoto, M., Tamai, K., Mikawa, R., Hirai, T., Murakami, K., et al. (2021). Disease modeling of pulmonary fibrosis using human pluripotent stem cell-derived alveolar organoids. Stem Cell Reports *16*, 2973–2987.

5. Ebisudani, T., Sugimoto, S., Haga, K., Mitsuishi, A., Takai-Todaka, R., Fujii, M., Toshimitsu, K., Hamamoto, J., Sugihara, K., Hishida, T., et al. (2021). Direct derivation of human alveolospheres for SARS-CoV-2 infection modeling and drug screening. Cell Rep. *35*, 109218.
