# Supplementary material for: Screening of factors inducing alveolar type 1 epithelial cells using human pluripotent stem cells
Source: Stem Cell Reports. 2024 Mar 28;19(4):529–44. doi: 10.1016/j.stemcr.2024.02.009 (PMC11096435; doi:10.1016/j.stemcr.2024.02.009)
Supplement: Document S2. Article plus supplemental information [file mmc3.pdf]

Stem Cell Reports
Article
OPEN ACCESS

Screening of factors inducing alveolar type 1 epithelial cells using human pluripotent stem cells

Yuko Ohnishi,[1,2] Atsushi Masui,[1,2] Takahiro Suezawa,[1] Ryuta Mikawa,[1,2] Toyohiro Hirai,[3] Masatoshi Hagiwara,[4] and Shimpei Gotoh[1,2,5,*]
[1]Department of Drug Discovery for Lung Diseases, Graduate School of Medicine, Kyoto University, Kyoto 606-8501, Japan
[2]Center for iPS Cell Research and Application (CiRA), Kyoto University, Kyoto 606-8507, Japan
[3]Department of Respiratory Medicine, Graduate School of Medicine, Kyoto University, Kyoto 606-8507, Japan
[4]Department of Anatomy and Developmental Biology, Graduate School of Medicine, Kyoto University, Kyoto 606-8501, Japan
[5]Lead contact
*Correspondence: gotoh.shimpei.5m@cira.kyoto-u.ac.jp
https://doi.org/10.1016/j.stemcr.2024.02.009

SUMMARY

Alveolar type 2 (AT2) epithelial cells are tissue stem cells capable of differentiating into alveolar type 1 (AT1) cells for injury repair and maintenance of lung homeostasis. However, the factors involved in human AT2-to-AT1 cell differentiation are not fully understood. Here, we established *SFTPC*^{GFP} and *AGER*$^{mCherry\text{-}HiBiT}$ dual-reporter induced pluripotent stem cells (iPSCs), which detected AT2-to-AT1 cell differentiation with high sensitivity and identified factors inducing AT1 cell differentiation from AT2 and their progenitor cells. We also established an "on-gel" alveolar epithelial spheroid culture suitable for medium-throughput screening. Among the 274 chemical compounds, several single compounds, including LATS-IN-1, converted AT1 cells from AT2 and their progenitor cells. Moreover, YAP/TAZ signaling activation and AKT signaling suppression synergistically recapitulated the induction of transcriptomic, morphological, and functionally mature AT1 cells. Our findings provide novel insights into human lung development and lung regenerative medicine.

INTRODUCTION

Lung alveoli are a highly functional part of the lung and consist of 300 million small pouch-like structures. The alveolar epithelium comprises alveolar type 2 (AT2) and alveolar type 1 (AT1) epithelial cells. AT2 cells are cuboidal in shape and secrete pulmonary surfactants, which maintain the alveolar structure by reducing alveolar surface tension. AT2 cells are tissue stem cells that maintain alveolar homeostasis and regeneration after injury via self-renewal and differentiation into AT1 cells (Barkauskas et al., 2013). AT1 cells are thin and flat, covering 95% of the alveolar surface, and are responsible for gas exchange. AT1 cells differentiate from bipotent alveolar epithelial progenitor cells in the fetal lung (Desai et al., 2014; Zepp et al., 2021). Abnormal alveolar epithelial cells with transitional profiles between AT2 and AT1 cells exist in the lungs, such as idiopathic pulmonary fibrosis, and in *in vivo* models such as bleomycin mice (Choi et al., 2020; Kobayashi et al., 2020; Strunz et al., 2020), suggesting the abnormalities of the differentiation process in the diseased lungs.

Signals that regulate the differentiation of AT2-to-AT1 cells have been explored. Among them, the activation of yes-associated protein and the transcriptional coactivator with PDZ-binding motif (YAP/TAZ) signaling is essential for AT1 cell differentiation (DiGiovanni et al., 2023; Gokey et al., 2021; LaCanna et al., 2019; Liu et al., 2016; Nguyen et al., 2021; Penkala et al., 2021; Warren et al., 2023). Other signals such as Notch, transforming growth factor β (TGF-β), Wnt, and p53 are also involved in AT2-to-AT1 cell differentiation (Finn et al., 2019; Frank et al., 2016; Kaiser et al., 2023; Kanagaki et al., 2021; Zhao et al., 2013). However, most reports are based on mouse studies and have yet to be validated enough using human alveolar epithelial cells. Historically, human AT2 cell lines for studying AT2-to-AT1 cell differentiation are lacking. Human primary AT2 cells are available for long-term culture in organoids; however, their limited supply has hampered an in-depth study of AT1 cells. Data on the human AT2-to-AT1 cell differentiation pathway remain limited. Human induced pluripotent stem cells (iPSCs) are useful for studying human alveolar epithelial cells (Gotoh et al., 2014; Yamamoto et al., 2017). A long-term culture of iPSC-derived AT2 (iAT2) cells was established, whereas iPSC-derived AT1 (iAT1) cells depended on the culture system. In Matrigel-embedded organoids, AT1 cell marker genes increased in coculture with human fetal lung fibroblasts (HFLFs), whereas they were almost negative under feeder-free conditions (Alysandratos et al., 2022; Kanagaki et al., 2021). iPSC-derived mesenchymal cells induced iAT2 and iAT1 cells from both carboxypeptidase M$^+$ (CPM$^+$) lung progenitor cells and iAT2 cells (Tamai et al., 2022), indicating that differentiation into iAT1 cells requires signaling from a coculture with mesenchymal cells. Based on these findings, we screened compounds that convert alveolar epithelial cell fate in a nonbiased setting without feeder mesenchymal cells. In this study, we established a dual-reporter iPSC line harboring *SFTPC*^{GFP} and *AGER*$^{mCherry\text{-}HiBiT}$ to evaluate AT2-to-AT1 cell differentiation quantitatively with high sensitivity and established an "on-gel" culture of iAT2 cells

This is an open access article under the CC BY license (http://creativecommons.org/licenses/by/4.0/).

suitable for medium-throughput screening. Combining dual-reporter iPSCs and on-gel culture, we screened 274 compounds to find those that increased HiBiT luminescence, leading to comprehensive signaling pathways. Among these, LATS-IN-1, an activator of YAP/TAZ signaling via inhibition of LATS1/2 kinases, and BAY1125976, an AKT inhibitor, synergistically promoted AT1 cell differentiation.

RESULTS

Generation of a dual-reporter iPSC line with *SFTPC^GFP^* and *AGER^mCherry-HiBiT^*

To quantitatively detect AT1 cell differentiation in a highly sensitive manner, we generated a dual-reporter iPSC line by knocking in the *mCherry-HiBiT* gene at the locus of *AGER* in the $SFTPC^{GFP}$ reporter iPSC line (B2-3 clone used for detecting AT2 cells) (Gotoh et al., 2014) using the CRISPR-Cas9 system (Figure 1A). *AGER*, expressed in AT1 cells, is a marker gene in human and mouse AT1 cells (Chung and Hogan, 2018; Dahlin et al., 2004; Guo et al., 2023). PCR confirmed that the reporter gene was knocked in into a single allele and that the drug resistance gene cassette was removed by introducing Cre recombinase (Figure S1A). The karyotype was normal (Figure S1B). The dual-reporter iPSCs were differentiated into CPM^{hi} lung progenitor cells, as previously described (Yamamoto et al., 2017), and the induction efficiency was similar to that of the parental cell line (Figures 1B and S1C). Alveolarized epithelial cells co-cultured with HFLF showed increased *AGER* and *mCherry* gene expression and HiBiT luminescence (Figures 1C and 1D). Immunofluorescence staining showed that almost all of the $AGER^{+}$ cells were identical to $mCherry^{+}$ cells and that $AGER^{+}$ cells expressed HT1-56, another AT1 cell marker (Figures 1E and 1F).

On-gel culture of fibroblast-free alveolar epithelial spheroids for screening

Since our previously reported fibroblast-dependent alveolar organoids (FD-AOs) and fibroblast-free alveolar organoids (FF-AOs) were embedded in Matrigel, they were not ideal for screening due to the requirement for careful mixing of the cells in Matrigel (Yamamoto et al., 2017). In addition, air-liquid interface cultures need 520,000 cells/cm^2 to apply these culture methods to screening (Hekman et al., 2020). We established a new alveolar epithelial cell culture method, on-gel culture, for compound screening. A total of 5×10^3 of CPM^{hi} lung progenitor cells or 1×10^4 of GFP^{+} iAT2 cells were seeded on thick-coated Matrigel in 96-well plates and cultured in DCIK+2i (CHIR99021 and SB431542) medium for 12 days, resulting in the formation of spheroids on Matrigel, exposing themselves to culture medium (Figures 2A and 2B). GFP fluorescence indicating the presence of iAT2 cells showed that the rate of GFP^{+} cells was significantly higher in iAT2 cell-derived on-gel spheroids than FD-AOs (Figures 2B and 2C). *SFTPC* expression was markedly higher in on-gel culture than FD-AOs up to the level in human adult lungs (Figure 2D). Another AT2 cell marker, *LAMP3*, and a lung epithelial cell marker, *NKX2-1*, did not differ significantly between the two culture methods. Although *AGER* expression in iAT2 cell-derived on-gel spheroids (passages 1-3) was equivalent between the FD-AOs, the expression levels were low compared to those in the adult lung. The expression of other AT1 cell markers, such as *CAV1*, *CLIC5*, and *GPRC5A* was lower in the on-gel culture than FD-AOs, indicating that iAT1 cells were barely present or immature in the on-gel culture (Figure 2D). Immunofluorescence staining confirmed the presence of iAT2 cells and AT1 marker-positive cells (Figures 2E and 2F). RNA sequencing (RNA-seq) analysis showed that on-gel spheroids tended to express the highest levels of AT2 and club cell marker genes among CPM^{hi} lung progenitor cells and epithelial cells from FD-AOs and FF-AOs (Figures S2A and S2B). FD-AOs expressed AT1 and airway epithelial cell marker genes more strongly than FF-AOs and on-gel spheroids. In contrast, FF-AOs expressed each lineage marker gene at a lower level compared with FD-AOs and on-gel spheroids. Venn diagrams of the differentially expressed genes (DEGs) extracted from each culture method showed that 370 genes were specifically upregulated in the on-gel culture, and Gene Ontology (GO) analysis showed that GOs related to lipid synthesis and metabolism, which are characteristics of AT2 cells, were enriched (Figures S2C and S2D). Furthermore, lamellar body structures, a characteristic of functional AT2 cells, were observed in the cytoplasm of on-gel-cultured iAT2 cells, and tubular myelin-like structures were observed on the luminal side of the spheroid (Figure 2G). In contrast, 895 genes were explicitly reduced in the on-gel culture, and GOs related to DNA replication and cell proliferation were enriched, indicating that on-gel culture is characterized by a lower proliferative potential of iAT2 cells compared to the other two culture methods (Figures S2E and S2F). These data demonstrate that on-gel culture is suitable for the culture of alveolar spheroids (which consist of epithelial cells alone) and it requires only a small number of cells, allowing the culture of iAT2 cells with easy handling.

Compound screening for searching AT2-to-AT1 cell differentiation signal

We screened for compounds that convert AT2-to-AT1 cells by combining dual-reporter iPSCs and on-gel culture method. Due to the readily available cell numbers, we used CPM^{hi} lung progenitor cells for screening (Figure 3A).

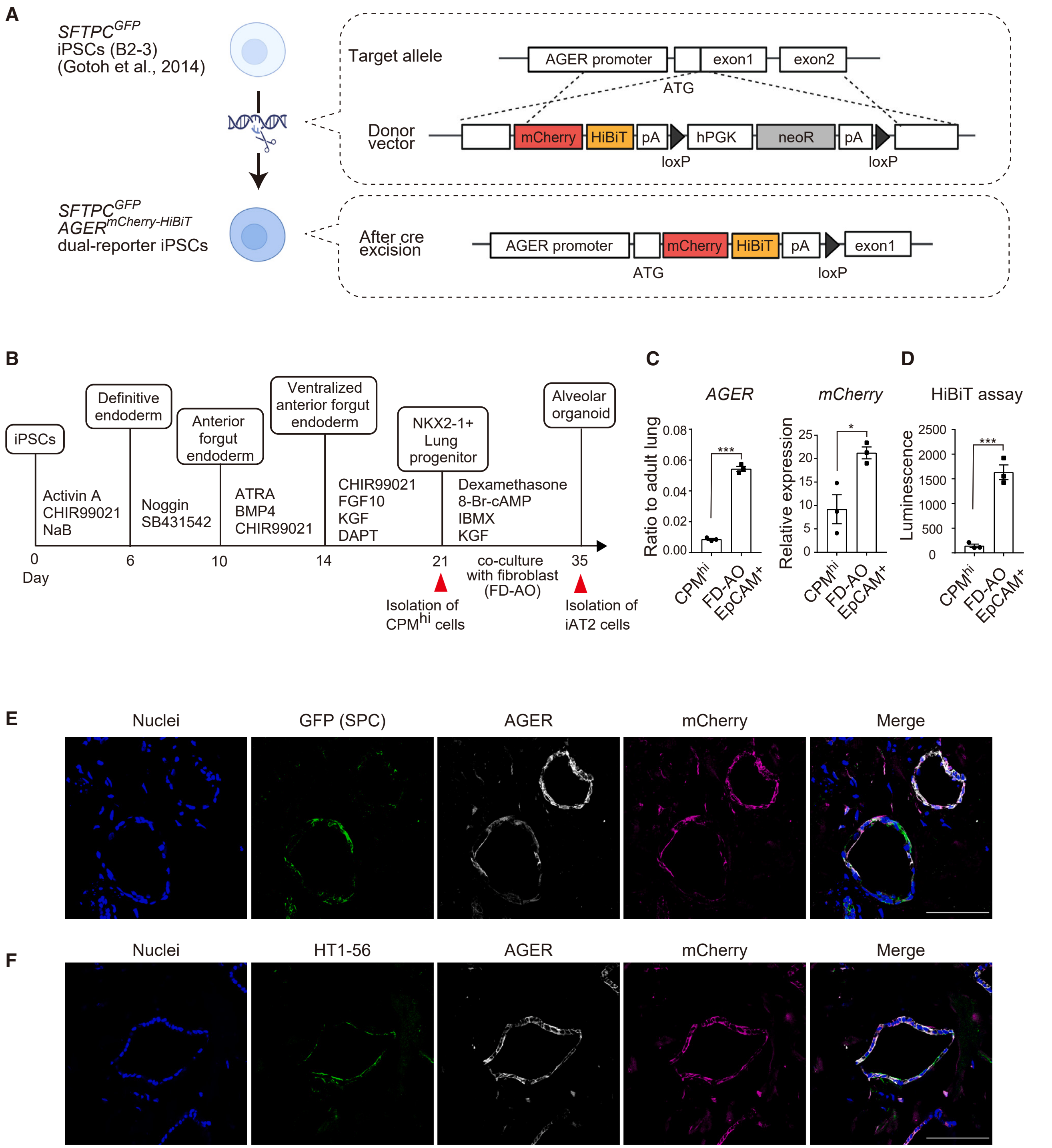

Figure 1. Generation of an *SFTPC*^{GFP} *AGER*$^{mCherry\text{-}HiBiT}$ dual-reporter iPSC line

(A) Schematic illustration of the strategy used to build a dual-reporter iPSC line by gene editing.

(B) Schematic diagram of the stepwise differentiation from iPSCs into iAT2 cells.

(C) Comparison of mRNA expression levels in CPM^{hi} lung progenitor cells and MACS-isolated EpCAM^{+} cells from FD-AOs on day 35 (B). Data are shown as mean ± SEM (n = 3 from independent experiments). Unpaired 2-tailed Student's t test; *$p < 0.05$; ***$p < 0.005$.

(D) Comparison of HiBiT luminescence between CPM^{hi} lung progenitor cells and MACS-isolated EpCAM^{+} cells from FD-AOs on day 35 (B). Data are shown as mean ± SEM (n = 3 from independent experiments). Unpaired 2-tailed Student's t test; ***$p < 0.005$.

(E and F) Immunofluorescence imaging of FD-AOs. Scale bars: 100 μm.

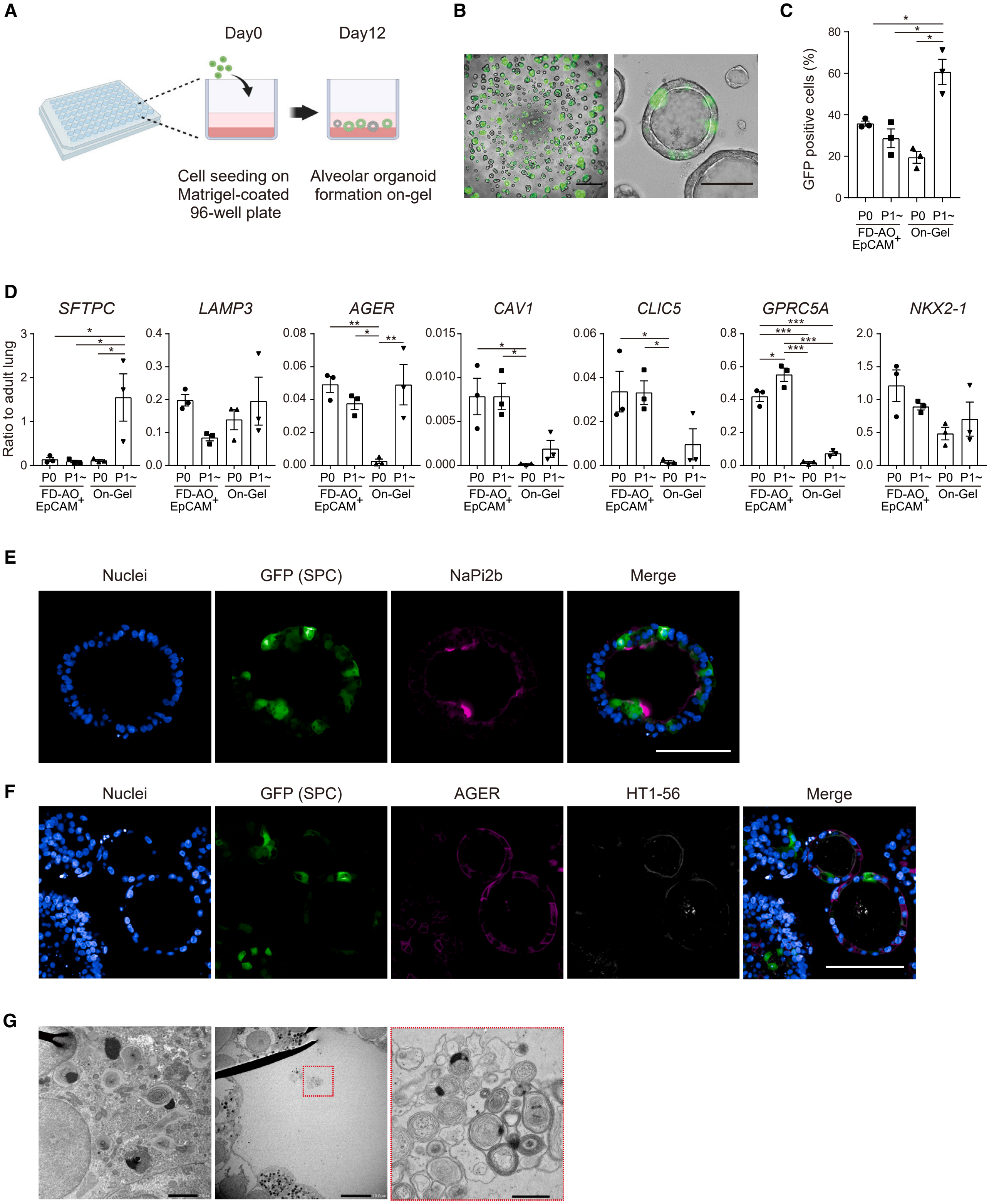

Figure 2. On-gel alveolar epithelial spheroid culture

(A) Schematic representation of the on-gel culture.

(B) Live-cell imaging of on-gel culture of alveolar spheroids derived from GFP+ iAT2 cells. Left scale bar, 500 μm; right scale bar, 100 μm.

(legend continued on next page)

The chemical library of 274 compounds (Table S1) was applied at 10 μM for 3 days, and the compounds were narrowed down using the HiBiT luminescence values corrected using the Cell Counting Kit 8 (CCK8) absorbance values as an indicator. As a result, 15 compounds exceeded the hit criteria, >1.5 SD, whereas 15 were judged to have high cytotoxicity based on the CCK8 values (Figure 3B). These hit compounds were validated for their effects on AT1 marker gene expression in GFP^+ iAT2 cell-derived spheroids (Figures 3C and 3D). Among these compounds, LATS-IN-1 upregulated *AGER*, *CLIC5*, and *CAV1*. LATS-IN-1, an inhibitor of LATS1 and LATS2, activates YAP/TAZ signaling by inhibiting YAP/TAZ phosphorylation (Kastan et al., 2021). YAP/TAZ signaling is essential for AT1 cell differentiation, and recently, LATS-IN-1 reportedly promoted the differentiation of iPSC-derived AT2 cells into AT1 cells (Burgess et al., 2023). However, LATS-IN-1 clumped the spheroids and promoted cell proliferation, which did not induce cell thinning or quiescence, a hallmark of AT1 cells (Figures S3A–S3D). ROCK-IN-2, an inhibitor of rho-associated protein kinase 2 (ROCK2), elevated AT1 gene expression and clumped spheroids, similar to LATS-IN-1. Since a recent study reported that stretching increased the number of AT1 cells via the ROCK-YAP pathway in mice (Nguyen et al., 2021), we did not expect that ROCK-IN-2 promoted AT1 differentiation. To determine whether ROCK-IN-2 promotes AT1 differentiation via on-target effects, Y-27632 and Fasudil HCl, other ROCK inhibitors, were tested. However, these compounds did not upregulate AT1 gene expression (Figures S3E and S3F). Furthermore, ROCK-IN-2 upregulated the expression of genes downstream of YAP/TAZ signaling, *CCN2*, *ANKRD1*, and *CYR61*, suggesting ROCK-IN-2 as an off-target regulator of YAP/TAZ signaling. NVP-AEW541 upregulated AT1 marker gene expression but was excluded from further studies because it was considered cytotoxic in iAT2 cells based on CCK8 values and cell morphology.

Combination of active YAP/TAZ signaling with AKT signal suppression promotes AT2-to-AT1 cell differentiation

Tofacitinib, FM381, BAY1125976, and SCH772984 were suspected to upregulate the three AT1 marker genes (Figure 3D). However, since the efficacy of each of these compounds was weak, we evaluated their synergistic effect in combination with the activation of YAP/TAZ signaling for AT1 cell differentiation. Among these compounds, only BAY1125976, an allosteric inhibitor of AKT, and LATS-IN-1 synergistically increased the expression of AT1 cell marker genes (Figures 4A and S4A). AKT signaling functions as a hub for multiple pathways, including cell survival and proliferation, and is important for AT2 cell proliferation (Portnoy et al., 2004). A few reports have suggested its involvement in AT2-to-AT1 cell differentiation (Zhang et al., 2023; Zhong et al., 2022), although it has not been sufficiently evaluated. Furthermore, since recent transcriptomic analysis indicated that AKT signaling can be affected in the transitional state of alveolar epithelial cells observed in damaged fibrotic lungs (Kobayashi et al., 2020; Xu et al., 2016), we hypothesized that AKT signaling may be associated with AT2-to-AT1 cell differentiation. Since YAP signaling has been reported to positively regulate AKT signaling (Gokey et al., 2018), it is intriguing that AKT inhibition may be involved in AT1 differentiation in concert with LATS-IN-1. We determined the optimal concentrations of LATS-IN-1 and BAY1125976 to activate YAP/TAZ and inhibit AKT, respectively. Phosphorylated YAP was decreased in a LATS-IN-1 dose-dependent manner, whereas total YAP expression peaked at 10 μM; hence, subsequent experiments were performed at 10 μM (Figure S4B). BAY1125976 inhibited phosphorylated AKT at 10 μM; therefore, subsequent experiments were set at 10 μM (Figure S4C). Since AKT signaling suppression triggers apoptosis (Coffey et al., 2005), the initiation of apoptosis may have influenced the AT1 cell marker gene expression. To test this, we investigated whether the two apoptotic inducers, Camptothecin and Staurosporine, contribute to AT1 cell marker gene expression. The apoptotic inducers and BAY1125976 significantly increase caspase activity; however, camptothecin and staurosporine did not increase AT1 cell marker gene expression (Figures S4D and S4E). Furthermore, Alpelisib and AZD6482, PI3K (phosphatidylinositol 3-kinase)/AKT pathway inhibitors, upregulated AT1 cell marker gene expression synergistically with LATS-IN-1 (Figure S4F). Therefore, we focused on the relationship between YAP/TAZ and AKT signaling using LATS-IN-1 and BAY1125976.

(C) GFP^+ rate of epithelial cells in FD-AOs and on-gel alveolar spheroids. Data were obtained from passage 0 (P0) CPM^{hi} lung progenitor cells or P1-4 GFP^+ iAT2 cell-derived cells. Data are shown as mean ± SEM (n = 3 from independent experiments). One-way ANOVA with Tukey's multiple comparison test; *$p < 0.05$.
(D) mRNA expression in the epithelial cells of FD-AOs and on-gel alveolar spheroids (P0 or P1-4). P1-4 on-gel alveolar spheroids were derived from P0-3 FD-AOs. Data are shown as mean ± SEM (n = 3 from independent experiments). One-way ANOVA with Tukey's multiple comparison test; *$p < 0.05$; **$p < 0.01$; ***$p < 0.005$.
(E and F) Whole-mount immunofluorescent imaging of the on-gel alveolar spheroids on-gel derived from P1. Scale bars, 100 μm.
(G) Transmission electron microscopy imaging of lamellar bodies and tubular myelin-like structures of on-gel iAT2 cells. Left scale bar, 2 μm; center scale bar, 10 μm; right scale bar, 1 μm.

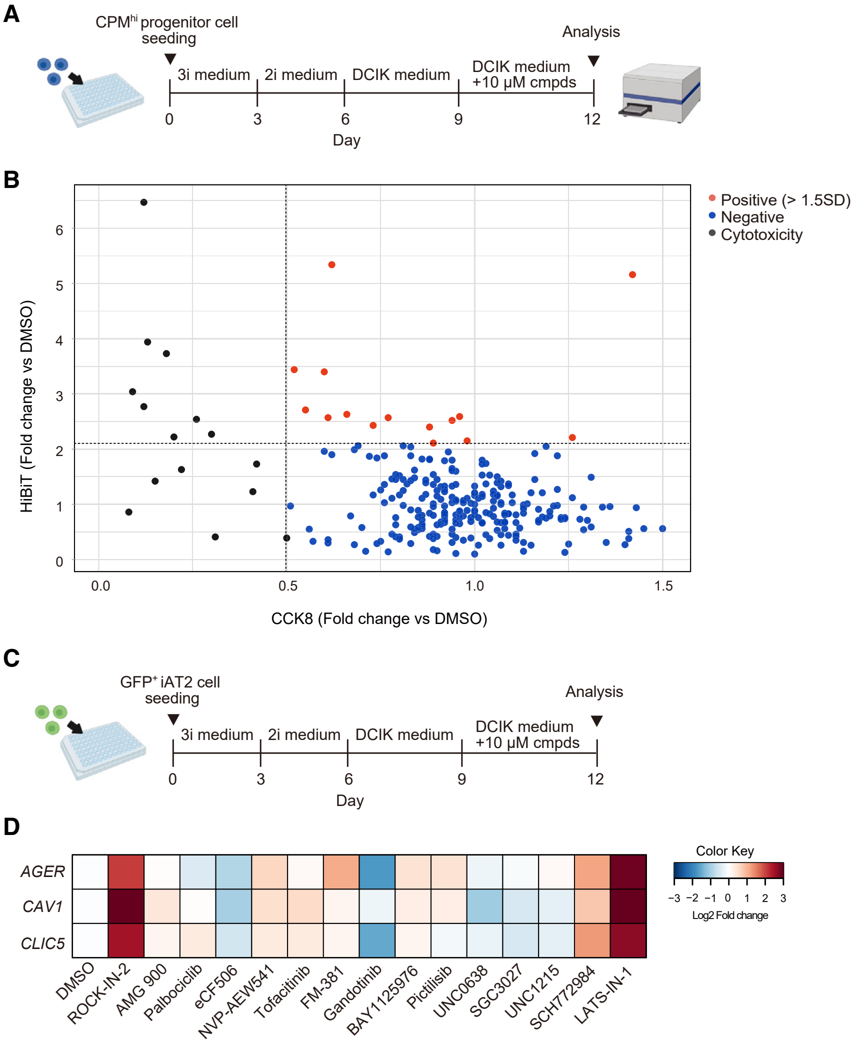

Figure 3. Compound screening using the *SFTPC*^{GFP} *AGER*$^{mCherry\text{-}HiBiT}$ dual-reporter iPSC-derived alveolar epithelial cells

(A) Schematic illustration of the strategy used for compound screening using the dual-reporter iPSC-derived CPM^{hi} lung progenitor cells.

(B) Compound screening using HiBiT luminescence and CCK8 absorbance as indicators. Compounds with CCK8 values <0.5 times the value of DMSO were excluded as cytotoxic, and 1.5 times the SD value of HiBiT/CCK8 of the remaining compounds was used as the hit criteria.

(C) Schematic illustration of the strategy used for the validation of the hit compounds using dual-reporter iPSC-derived GFP^{+} iAT2 cells.

(D) mRNA expression of on-gel GFP^{+} iAT2 cell-derived spheroids treated with 10 μM hit compounds. The heatmap was created with the value of $\log_2$ (fold change vs DMSO). n = 3 from independent experiments.

Along with the increased expression of *AGER* and other AT1 marker genes, cotreatment with the two compounds enhanced mCherry fluorescence (Figure 4B). Interestingly, BAY1125976 increased *SFTPC* gene expression, and co-treatment with the two compounds slightly increased its expression compared to that of LATS-IN-1 alone, suggesting that BAY1125976 suppressed cell proliferation and maintained AT2 marker gene expression. The immunofluorescence data showed that LATS-IN-1 and its combination with BAY1125976 decreased the number of GFP single-positive spheroids and increased the number of AT1 marker-positive spheroids (Figures 4C–4E). In particular, in treatment with the combination of LATS-IN-1 and BAY1125976, 89.3% of the spheroids were positive for at least either AGER or HT1-56. NKX2-1 was expressed in all of the cells derived from the repeatedly passaged GFP^{+} cells, suggesting that alveolar epithelial cell lineage was maintained (Figure S4G). Among the GFP and AGER double-negative cells, surfactant protein B (SPB)-positive cells were also present, suggesting that immature or distal lung AT2 subpopulations were present in the spheroids (Figure S4H).

To confirm the reproducibility, another iPSC line, ChiPSC18, was differentiated into CPM^{hi} lung progenitor cells, and the isolated CPM^{hi} cells were cocultured with HFLFs to generate FD-AOs (Figures S5A and S5B). After 2 weeks of coculture, EpCAM^{+} NaPi2b^{+} iAT2 cells were isolated using fluorescence-activated cell sorting (FACS) and subjected to on-gel culture to test the compounds (Figure S5C). Unlike the dual-reporter iPSC line, no conspicuous clumped morphological changes were observed following LATS-IN-1 treatment (Figure S5D). As expected, AT1 marker gene expression was significantly increased following cotreatment with both compounds (Figure S5E). Furthermore, we determined whether these findings could be reproduced in human primary AT2 cells. We observed

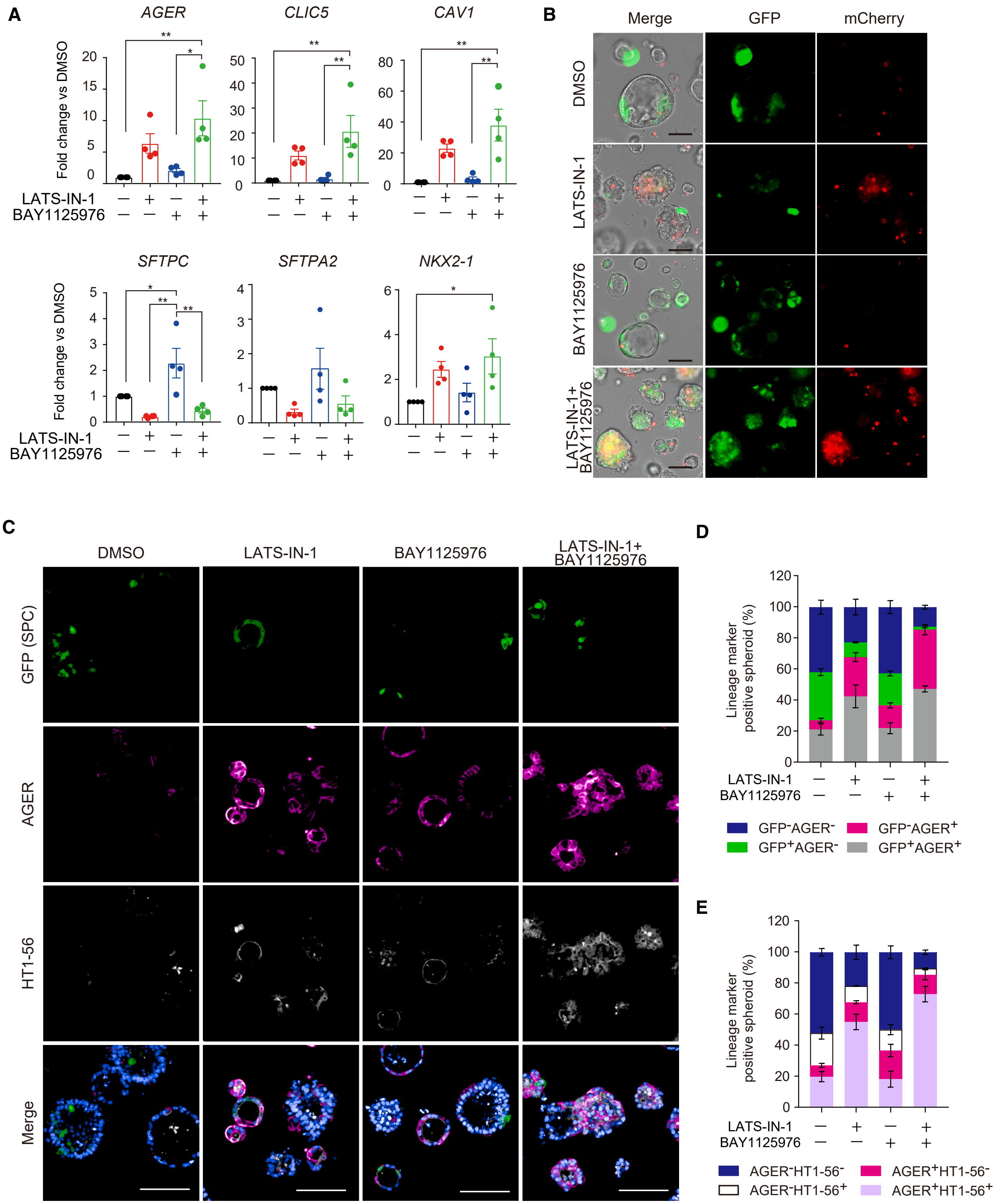

(legend on next page)

intracellular lamellar bodies and tubular myelin-like structures on the luminal side of the spheroids after 2 weeks of dome culture, suggesting that functional AT2 cells were maintained in the spheroids (Figures 5A–5C). We isolated HT2-280^{+} AT2 cells, subjected them to on-gel culture, and treated the spheroids with the above-mentioned compounds (Figure 5D). In contrast to iPSC, no increase in clumped forms was observed in primary AT2 cell-derived spheroids by LATS-IN-1 treatment (Figure 5E). A synergistic upregulation of the *AGER* gene was observed upon compound cotreatment, consistent with the immunofluorescence staining (Figures 5F and 5G).

Mechanistic analysis of on-gel spheroids upon compound treatment

AKT is downstream of keratinocyte growth factor (KGF) and suppresses the AT1 cell differentiation (Borok et al., 1998). Therefore, it is also possible that this observation is attributed to the incorporation of KGF in the differentiation media. However, even upon the removal of KGF from the media, the synergistic impact of the two compounds persisted, and the effect was augmented (Figures 6A and 6B). The absence of KGF did not induce changes in AT1 cell gene expression or reduce *SFTPC* expression. However, the removal of KGF decreased *SFTPA2* expression, implying that although KGF is essential for AT2 cell maintenance, it is not directly involved in the differentiation into AT1 cells. Increased phosphorylated AKT was observed under LATS-IN-1 treatment in three-dimensional (3D) culture (Figure 6C), suggesting that YAP/TAZ activation is essential for AT2-to-AT1 cell differentiation but may also activate AKT and interrupt the complete differentiation process. Intriguingly, AKT phosphorylation was spontaneously reduced under two-dimensional (2D) culture conditions with LATS-IN-1 treatment, in which cells were flattened, compared with the DMSO-treated cells (Figure 6D), suggesting that the regulation of AKT by YAP/TAZ depends on the microenvironment.

AT1 cell maturation is promoted by active YAP/TAZ with suppressed AKT signaling

To further understand the effect of cotreatment with LATS-IN-1 and BAY1125976 on cell differentiation, we performed bulk RNA-seq analysis. Principal-component analysis (PCA) of the 16,430 genes showed that each compound-treated sample was divided into clusters (Figure 7A). In the PCA and Venn diagrams, BAY1125976-treated cells had a gene expression profile that was relatively close to that of the DMSO-treated control group, whereas each of the LATS-IN-1-treated and the two compound cotreated cells had distinct gene expression profiles that were far from the control group (Figures 7A and 7B). We next used the gene set of TRAVAGLINI_LUNG_ALVEOLAR_EPITHELIAL_TYPE1 (Travaglini et al., 2020) to comprehensively analyze the transcriptomes of each condition for elaborating the gene expression changes that characterize AT1 cells other than *AGER* (Figure 7C). LATS-IN-1 alone broadly increased the expression of AT1 cell marker genes, whereas cotreatment with LATS-IN-1 and BAY1125976 induced them more broadly and robustly. Next, we performed GO analysis to gain insight into how cotreatment with the compounds affected cellular biological processes compared to LATS-IN-1 treatment alone (Figure 7D). GOs related to AT1 cell characteristics, such as "vasculature development" and "response to decreased oxygen levels" and actin cytoskeleton, were ranked higher in the cotreated group. Since AT1 cells secrete multiple angiogenic factors and facilitate tissue formation during alveolar development (Zepp et al., 2021), angiogenic factors in the culture medium were measured (Figure 7E). Vascular endothelial growth factor A (VEGFA) was not synergistically increased following cotreatment with LATS-IN-1 and BAY1125976; however, sonic hedgehog (SHH) was predominantly elevated in the cotreated spheroids compared with the DMSO-treated control. The transcriptome of AT1 cells is enriched in genes associated with actin cytoskeleton regulation, suggesting that actin cytoskeleton determines AT1 cell morphology (Penkala et al., 2021; Shiraishi et al., 2023). We hypothesized that these reports and the GOs associated with the actin cytoskeleton could be the reminiscent of the thin-and-flat morphological changes characteristic of AT1 cells; however, it was impossible to measure the thickness and diameter of the spheroids in on-gel culture because LATS-IN-1 induced clumped morphological changes. Therefore, we analyzed the morphology in FD-AOs and found that LATS-IN-1 did not thin the organoids but rather thickened them,

Figure 4. Synergistic effect of YAP/TAZ activation and AKT suppression on AT2-to-AT1 cell differentiation

(A) mRNA expression in dual-reporter iAT2 cell-derived on-gel spheroids (P1). Each column represents samples treated with 10 μM LATS-IN-1, BAY1125976, or both. Data are shown as mean ± SEM (n = 3 from independent experiments). One-way ANOVA with Tukey's multiple comparison test; *$p < 0.05$; **$p < 0.01$.

(B) Live-cell imaging of iAT2 cell-derived on-gel spheroids treated with the compounds. Scale bars, 100 μm.

(C) Whole-mount immunofluorescence imaging of on-gel spheroids derived from GFP^{+} iAT2 using *SFTPC*^{GFP} reporter iPSC. Scale bars, 100 μm.

(D and E) Quantitative analysis of whole-mount images shown in (C). Data are shown as mean ± SEM (n = 3 from independent experiments using P1-2 iAT2 derived on-gel spheroids).

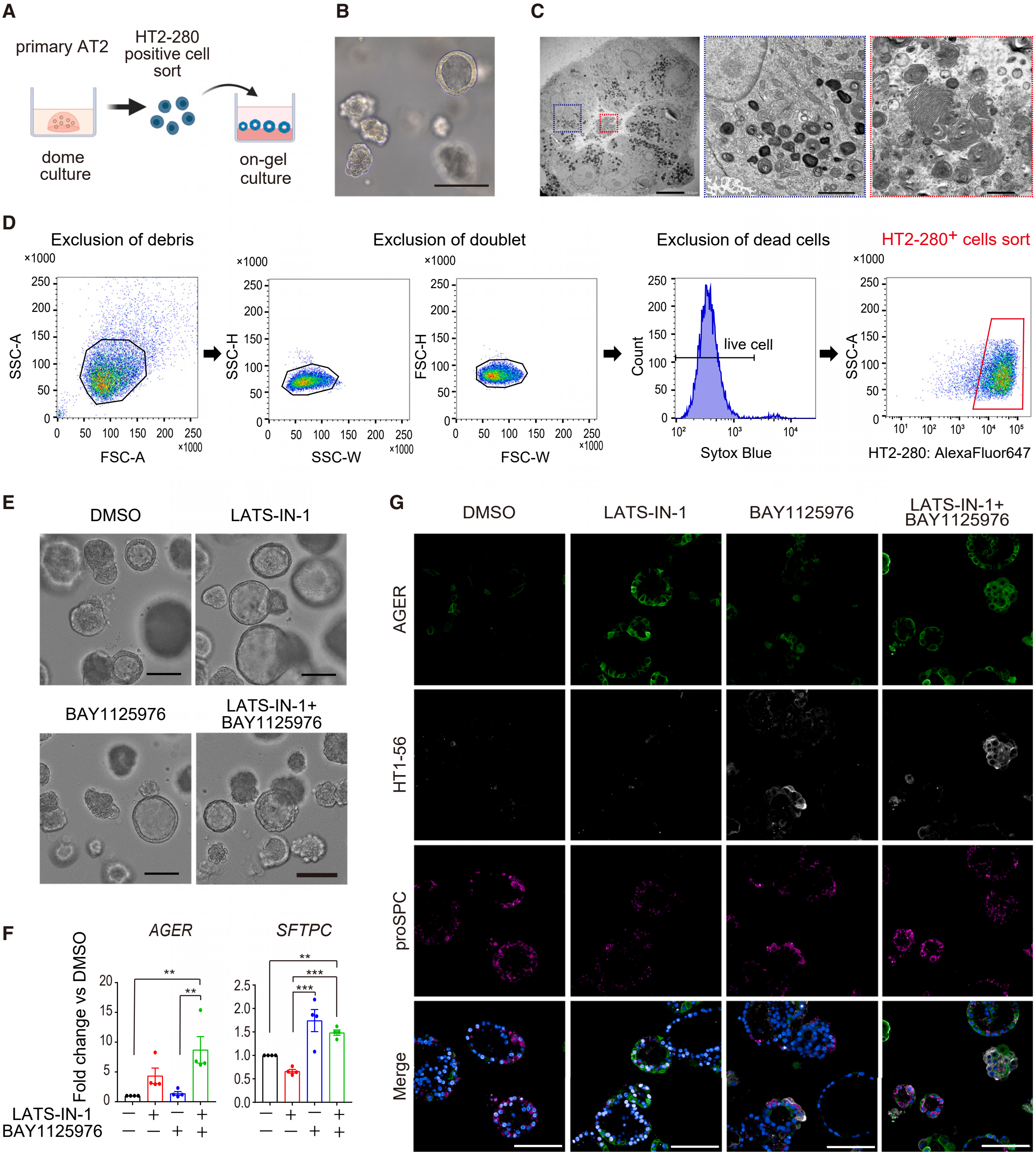

Figure 5. Validation of hit compounds using human adult primary AT2 cells

(A) Schematic representation of maintenance and on-gel culture of primary AT2 cells.

(B) Live-cell imaging of maintenance culture of primary AT2 cells. Scale bar, 100 μm.

(C) Transmission electron microscopy imaging of primary AT2 cell maintenance culture. Left: overall view of the spheroid. Scale bar, 10 μm. Center: enlarged view of the area boxed in the blue square on the left. Imaging of lamellar bodies in cells. Scale bar, 2 μm. Right: enlarged view of the area boxed in the red square on the left. Imaging of tubular myelin-like structures in the spheroid lumen. Scale bar, 500 nm.

(D) Gating strategy of flow cytometry for isolating HT2-280^{+} primary AT2 cells.

(legend continued on next page)

whereas BAY1125976 and cotreatment with LATS-IN-1 and BAY1125976 significantly thinned the organoids. In contrast, no significant change in organoid diameter was observed (Figures 7F and 7G). Alpelisib and AZD6482, PI3K/AKT inhibitors, consistently recapitulated the result of BAY1125976 (Figures S6A and S6B). Cotreatment with LATS-IN-1 and BAY1125976 increased AT1 marker-positive cells in FD-AOs (Figure 7H). These results suggest that the cotreatment with these two compounds reproduces not only the transcriptomic but also the functional and morphological features of iAT1 cells.

DISCUSSION

We identified the signaling pathways promoting AT2-to-AT1 cell differentiation by compound screening. Human iPSCs are a useful tool for studying lung alveolar differentiation, and we conceived a reporter cell-based screening method to determine signals that regulate AT2-to-AT1 cell differentiation. Fluorescent reporter genes help visualize differentiation, and HiBiT is superior to fluorescent reporters because it assesses changes in genes with low expression levels, such as *AGER*, sensitively and quantitatively. Many of the hit compounds, including BAY1125976, showed no apparent difference in mCherry fluorescence compared with the control group and would not have been detected without the HiBiT assay. In addition, we established a novel on-gel culture method of alveolar epithelial spheroids suitable for medium-throughput screening. On-gel culture is an epithelial cell-only culture system with advantages such as the ability to be cultured in 96-well plates, the small number of cells required per well, and the lack of complexity in embedding the cells in Matrigel. The on-gel culture enabled cell counting and the HiBiT assay to be performed in the same well. Furthermore, the spheroids were exposed on the outside of the gel, making the HiBiT substrate easily accessible to the spheroids without dissociation of the Matrigel. In the present study, multiple chemical compounds induced AT1 cell differentiation. Therefore, on-gel culture is beneficial for high-throughput screening or analyzing cell-based disease models using different markers in the future.

The chemical library used for screening contains several compounds that act on various intracellular signals, allowing for comprehensive evaluation of intracellular signaling. Since all of the compounds were used at 10 μM, false positives due to off-targeting remain a possibility. However, the library compensates for this concern since it contains multiple compounds per signal. Indeed, Alpelisib and AZD6482 did not reach hit criteria but increased HiBiT values (1.87- and 1.65-fold change vs DMSO, respectively), which helped identify the involvement of PI3K/AKT signaling in AT1 cell differentiation. Wnt signaling inhibitor and YAP/TAZ signaling activator were expected to be hits in the compound screening since we previously reported that XAV939, a suppressor of the canonical Wnt signaling via inhibition of tankyrase, promoted iAT1 cell differentiation from iAT2 cells in FD-AOs, and YAP/TAZ signaling is essential in AT1 cell differentiation (DiGiovanni et al., 2023; Gokey et al., 2021; Kanagaki et al., 2021; LaCanna et al., 2019; Liu et al., 2016; Nguyen et al., 2021; Warren et al., 2023). In the present study, we removed CHIR99021, which activates the canonical Wnt signaling via inhibition of GSK3β, from the medium before screening. Therefore, XAV939 was not a hit compound in this on-gel screening system without coculturing with fibroblasts. We speculated that fibroblasts could overactivate the Wnt pathway in AT2 cells in FD-AOs (Barkauskas et al., 2013; Kanagaki et al., 2021; Nabhan et al., 2018), whereas the canonical Wnt signaling was switched off by removing CHIR99021 from the medium during screening in an on-gel culture setting. XAV939 would have been a hit if the culture conditions had activated Wnt. LATS-IN-1 and ROCK-IN-2 showed the greatest increase in HiBiT luminescence among the hit compounds tested in this study, although ROCK-IN-2 appeared to stimulate YAP/TAZ signaling as an off-target effect. LATS-IN-1, which activates the YAP/TAZ signaling pathway, contributed significantly to AT1 differentiation, consistent with previous studies in mice and a recent study using human iPSC-derived AT2 cells (DiGiovanni et al., 2023; Gokey et al., 2021; LaCanna et al., 2019; Liu et al., 2016; Nguyen et al., 2021; Warren et al., 2023; Burgess et al., 2023). Our results provide insights into the relationship between PI3K/AKT signaling and AT1 differentiation. PI3K/AKT signal inhibition alone had a weak effect on the transcriptome, but when combined with LATS-IN-1, it significantly increased the expression of AT1 marker genes, suggesting that PI3K/AKT suppression under activated YAP/TAZ signaling is important. In this study, these two hit compounds promoted AT1 cell differentiation from CPM^{hi} progenitor cells as well as iAT2 cells. However, whether iAT2 cells transiently appear in the differentiation into AT1 cells from CPM^{hi} cells remains to be determined.

(E) Live-cell imaging of each compound-treated spheroid derived from primary AT2 cells. Scale bars, 100 μm.
(F) mRNA expression in on-gel primary AT2 cell-derived alveolar spheroids (P1-4). Each column represents samples treated with DMSO, 10 μM LATS-IN-1, 10 μM BAY1125976, or both. Data are shown as mean ± SEM (n = 4 from independent experiments). One-way ANOVA with Tukey's multiple comparison test; $^{**}p < 0.01$, $^{***}p < 0.005$.
(G) Whole-mount immunofluorescent imaging. Scale bars, 100 μm.

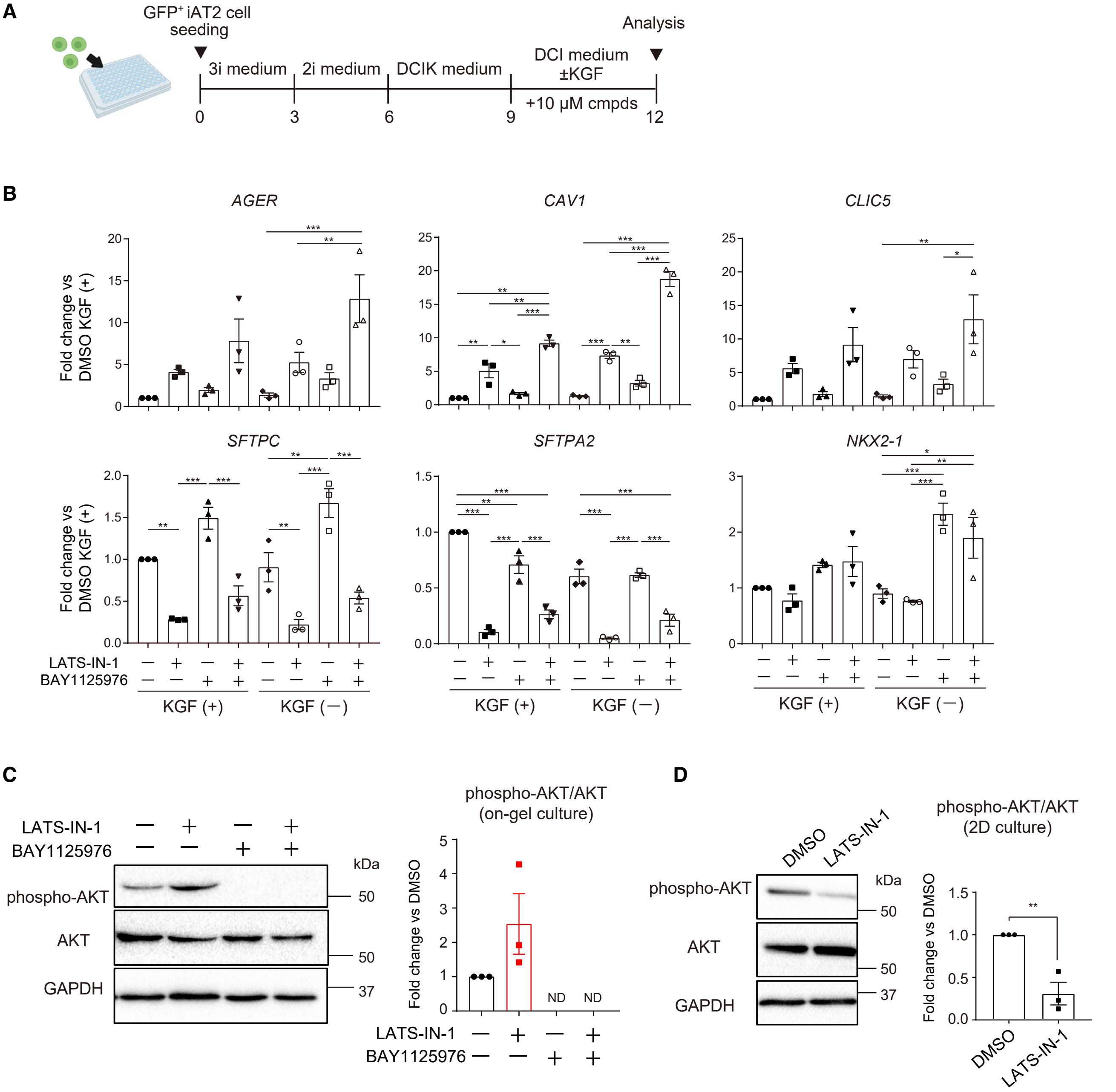

Figure 6. Mechanistic analysis of on-gel spheroids upon compound treatment

(A) Schematic illustration of the strategy used to analyze the impact of KGF in the culture medium on AT1 cell differentiation.

(B) AT1 and AT2 marker gene expression in *SFTPC^{GFP} AGER$^{mCherry\text{-}HiBiT}$* dual-reporter iAT2 cell-derived on-gel spheroids (P1). Each column represents samples treated with 10 μM LATS-IN-1, BAY1125976, or both, with or without KGF. Data are shown as mean ± SEM (n = 3 from independent experiments). One-way ANOVA with Tukey's multiple comparison test was used for analysis. Among several significant differences, only the comparison between the KGF contained group and KGF uncontained group was indicated; $*p < 0.05$; $**p < 0.01$; $***p < 0.005$.

(C) Western blotting of the on-gel alveolar epithelial spheroids using *SFTPC^{GFP}* reporter iPSC-derived CPM^{hi} lung progenitor cells. Spheroids were treated with 10 μM LATS-IN-1, 10 μM BAY1125976, or both. Phospho (Ser473)-AKT and total AKT bands were quantified and phospho-AKT bands were normalized to total AKT bands. Data are shown as mean ± SEM (n = 3 from independent experiments). ND, not detected.

(D) Western blotting of the 2D-cultured CPM^{hi} lung progenitor cells derived from *SFTPC^{GFP}* reporter iPSCs. Cells were treated with DMSO or 10 μM LATS-IN-1. Phospho (Ser473)-AKT and total AKT bands were quantified and phospho-AKT bands were normalized to total AKT bands. Data are shown as mean ± SEM (n = 3 from independent experiments).

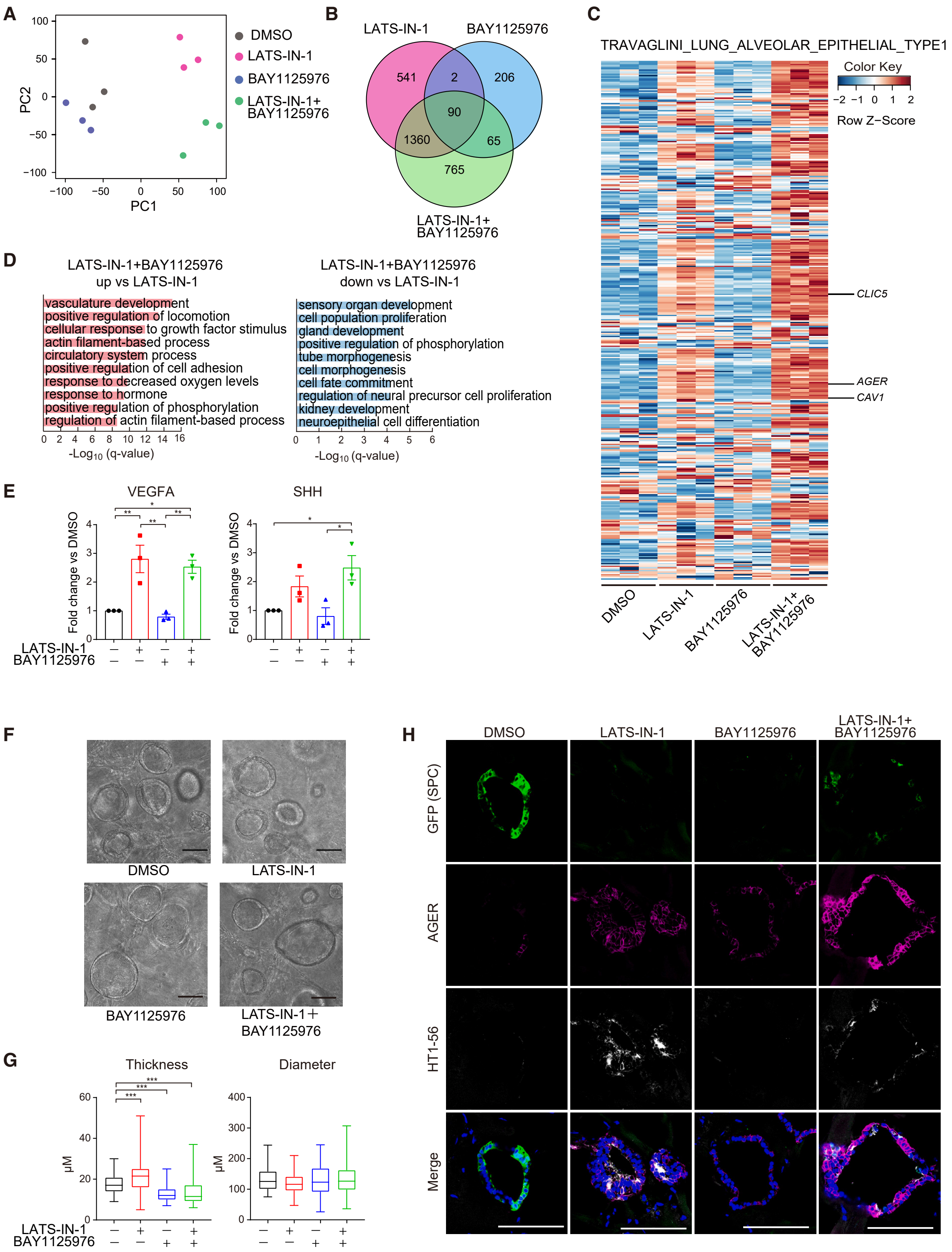

(legend on next page)

In the present study, treatment with LATS-IN-1 increased AT1 transcriptome broadly, but it alone did not thin the spheroids, consistent with recent *in vivo* reports that YAP/TAZ activation alone is insufficient for AT1 cell differentiation (Penkala et al., 2021). PI3K/AKT inhibitors induced thinning of the cells in FD-AOs, which is consistent with the recent report that AKT inhibition shortened stem cell length in the developing lung, indicating that AKT affects cell morphology in alveolar epithelial cells and is one of the factors controlling thinning (Liu et al., 2023). When dissociated AT2 cells were reseeded onto a 2D culture, the flattened morphology was achieved as well as increased AT1 cell markers (Kanagaki et al., 2021). Furthermore, activation of YAP/TAZ in the 2D culture spontaneously suppressed AKT (Figure 6D). We speculated that the change in the microenvironment of AT2 cells, such as the stiffness of the substrates or interactions with fibroblasts, was required for thinning during the AT1 cell differentiation along with the YAP/TAZ-related signal modulation. Nevertheless, the thinning of cells in the 3D culture conditions in the present study does not fully mimic the super-thin and super-flat AT1 morphology seen *in vivo*; hence, further modifications of the culture environment may better reproduce AT1 characteristics. In addition, because AKT can receive inputs from various signaling pathways other than KGF and YAP/TAZ signaling (Borok et al., 1998; Manning and Toker, 2017), AT1 differentiation may be regulated by some specific signals. Activated p53 signaling promotes AT1 cell differentiation, which is beneficial for suppressing lung adenocarcinoma (Kaiser et al., 2023). Activated p53 signaling induces PHLDA3, which represses AKT signaling and induces the apoptosis of cancer cells (Kawase et al., 2009), supporting our finding that AKT inhibition induced AT1 cells alternatively to activated p53 signaling and independently of YAP/TAZ signaling activation. Future studies are needed to elucidate the upstream role of AKT inhibition in AT1 cell differentiation.

In conclusion, we established a dual-reporter cell line for the quantitative evaluation of AT1 cell differentiation and a method of on-gel alveolar epithelial spheroid culture suitable for medium-throughput screening, identifying the activation of YAP/TAZ and inhibition of AKT as signals that promote AT1 differentiation. The findings of our study provide novel insight into human AT1 cell differentiation and contribute to lung regenerative medicine.

EXPERIMENTAL PROCEDURES

Resource availability

Lead contact

Further information and requests for resources and reagents should be directed to and will be fulfilled by the corresponding author, Shimpei Gotoh (gotoh.shimpei.5m@cira.kyoto-u.ac.jp).

Materials availability

Materials used in this study are available upon request under a completed materials transfer agreement.

Data and code availability

The accession number for the sequencing raw data reported in the present study is GEO: GSE241337.

On-gel culture and compound treatment

Four types of flat-bottom clear plates were used for the on-gel culture: a 96-well white plate (Falcon 353377) for HiBiT assay, a 96-well black plate (PerkinElmer 6055300) for whole-mount immunofluorescence, a 48-well clear plate (Greiner 677180) for western blotting, and a 96-well clear plate (Greiner 655180) for the other experiments. For whole-mount immunofluorescence and western blotting, a plate was coated with 50% Matrigel diluted in the DCIK+3i (3 μM CHIR99021, 10 μM SB431542, and 10 μM Y-27632) medium in volumes of 25 and 100 μL, respectively. For other experiments, a 96-well plate was coated with 50 μL of Growth Factor Matrigel per well at least 30 min before seeding cells. A total of 5×10^3 magnetic cell sorting (MACS)-isolated CPM^{hi} cells or 1×10^4 FACS-isolated iAT2 cells were suspended in 100 μL DCIK+3i medium and seeded onto a Matrigel-coated 96-well plate. A total of 8×10^4 MACS-isolated CPM^{hi} cells were

Figure 7. Effects of cotreatment with LATS-IN-1 and BAY1125976 on AT1 cell function and morphology
(A) PCA of the transcriptomes of iAT2 cell-derived on-gel spheroids under each treatment condition. Log_2 (transcripts per kilobase million [TPM] values) were used for the analysis. Data were obtained from 3 independent experiments.
(B) Venn diagram of DEGs that were upregulated in compound-treated cells compared with DMSO-treated cells. The threshold for upregulation was set to log_2 (fold-change) > 1, with an adjusted p value of <0.05.
(C) Heatmap of TRAVAGLINI LUNG ALVEOLAR EPITELIAL TYPE1 created with the value of log_2 (TPM + 0.01). Data were obtained from 3 independent experiments.
(D) Enrichment analysis based on GO biological processes. Left: increased following cotreatment with LATS-IN-1 and BAY1125976 compared with LATS-IN-1 alone. Right: decreased following cotreatment with LATS-IN-1 and BAY1125976 compared with LATS-IN-1 alone.
(E) VEGFA and SHH in the conditioned medium measured using ELISA. Data are shown as mean ± SEM (n = 3 from 3 independent experiments). One-way ANOVA with Tukey's multiple comparison test; *p < 0.05; **p < 0.01.
(F and G) Live-cell imaging of FD-AOs treated with the compounds and quantification of organoid thickness and diameter. Scale bars, 100 μm. Data are shown as mean ± SEM (n = 60 from 3 independent experiments; 20 organoids were randomly selected from each experiment). One-way ANOVA with Dunnett's multiple comparison test; ***p < 0.005.
(H) Immunofluorescence imaging of FD-AOs. Scale bars, 100 μm.

suspended in 250 μL DCIK+3i medium and seeded onto a Matrigel-coated 48-well plate. Y-27632 was withdrawn after day 3, and the medium was changed every 3 days until day 12. When the compounds were evaluated, 2i (CHIR99021 and SB431542) were withdrawn from day 6 to exclude their effect on the results, and each compound was supplemented on day 9. The list of chemicals in the library is provided in Table S1. When the effect of KGF was evaluated, KGF was withdrawn from the DCIK medium on day 9. Each experiment was performed on day 12.

HiBiT assay

Before the HiBiT assay, the cell count was measured using CCK8 (Dojindo CK04) according to the manufacturer's instructions. The cells were incubated in the medium containing 10% CCK8 at 37°C for 1 h. Then, the medium was transferred into a new clear 96-well plate, and its absorbance was measured. After the cells were washed once with the medium, the medium was replaced with 100 μL fresh DCIK medium, and 100 μL of HiBiT reagent (Promega N3030) was added to each well. The plates were mixed on a shaker for 10 min and incubated at room temperature (RT) for 10 min. HiBiT luminescence was measured using an ARVO X5 plate reader (PerkinElmer), and luminescence values were corrected with the CCK8 absorbance values. When the HiBiT assay was performed in cell suspension, 3×10^4 cells were suspended in 100 μL DCIK medium and mixed with 100 μL HiBiT reagent in a 1.5-mL tube. After vortexing and incubation at RT for 10 min, the samples were transferred to a white flat-bottom plate (Falcon 353296), and HiBiT luminescence was measured.

Whole-mount immunofluorescent analysis of on-gel spheroids

Spheroids on-gel were washed with PBS and fixed with 4% paraformaldehyde in PBS for 1 h at RT. Spheroids attached to the bottom of the plate were gently washed in PBS three times, permeabilized, and immersed in a blocking buffer (5% BSA, 1% Triton X-100 in PBS) for 1 h. Primary antibodies were diluted in the blocking buffer, and spheroids were stained for 2 h at RT and washed with a wash buffer (1% Triton X-100 in PBS) four times. Secondary antibodies and Hoechst33342 (Dojindo 346-07951) were diluted in the blocking buffer, and spheroids were stained for 1 h at RT. After washing four times in the wash buffer, each sample was immersed in PBS. Immunofluorescent images were obtained using OperaPhenix (PerkinElmer). The antibodies that were used for immunofluorescence are listed in Table S1.

VEGFA and SHH ELISA

The on-gel culture supernatant was collected and centrifuged at $200 \times g$ for 10 min at 4°C. The supernatant was used for ELISA, which was performed according to the kit's instructions (R&D Systems DVE00 and DSHH00). The number of cells on the plate from which the culture supernatant was removed was counted using CellTiter-Glo 2.0 Cell Viability Assay (Promega G9241), and the amount of VEGFA and SHH was corrected using the values of CellTiter-Glo.

Statistical analysis and software

Data are represented as mean ± SEM. Statistical analysis was performed with GraphPad Prism 7, and p values <0.05 were considered significant. FlowJo version 10.6.1 (Becton Dickinson) was used to draw flow cytometry plots and their related analysis. The illustrations were created with Biorender.com.

SUPPLEMENTAL INFORMATION

Supplemental information can be found online at https://doi.org/10.1016/j.stemcr.2024.02.009.

ACKNOWLEDGMENTS

We thank all of the members of the Gotoh lab (CiRA, Kyoto University); Y. Tsutsui, K. Nakao, S. Kanagaki, and K. Moriguchi (Kyorin Pharmaceutical) for experimental assistance and helpful discussion; A. Hotta (CiRA, Kyoto University) for CRISPR-related plasmids; R. Gerd (Ludwig Institute for Cancer Research) for the MX35 antibody; T. Yamamoto, K. Okita, K. Deguchi, and S. Sakurai (CiRA, Kyoto University) for RNA-seq analysis; K. Okamoto-Furuta and T. Katsuno (Division of Electron Microscopic Study, Center for Anatomical Studies, Kyoto University) for supporting transmission electron microscopy; all of the members of the Medical Research Support Center, Kyoto University for adjustment of laboratory equipment. This study was supported by Research Fund to Department of Drug Discovery for Lung Diseases at Kyoto University from Kyorin Pharmaceutical Co. Ltd. and by AMED (JP17bm0804007, JP23bm1423004, and JP23bm1323001), JSPS KAKENHI (JP22K19525), and the iPS Cell Research Fund (to S.G.). MX35 antibody production was supported by an NIH/NCI Cancer Center support grant (2P30 CA008748-53).

AUTHOR CONTRIBUTIONS

Conceptualization, Y.O., T.S., and S.G.; methodology, Y.O., A.M., and T.S.; validation, Y.O. and R.M.; formal analysis, Y.O. and S.G.; investigation, Y.O. and S.G.; resources, S.G.; writing, Y.O. and S.G.; supervision, T.H. and M.H.

DECLARATION OF INTERESTS

Y.O., A.M., and T.S. are employees, received research funding from, and are shareholders of Kyorin Pharmaceutical. R.M., T.H., M.H., and S.G. received research funding from Kyorin Pharmaceutical. S.G. and M.H. are founders and shareholders of HiLung. S.G. is an external board member of HiLung. S.G. is listed as one of the inventors of Kyoto University's patents related to the method of generating alveolar organoids: WO2014168264A1 and WO2016143803A1.

Received: September 3, 2023
Revised: February 26, 2024
Accepted: February 27, 2024
Published: March 28, 2024

REFERENCES

Alysandratos, K.-D., Garcia-de-Alba, C., Yao, C., Pessina, P., Huang, J., Villacorta-Martin, C., Hix, O.T., Minakin, K., Burgess, C.L., Bawa, P., et al. (2022). Culture impact on the transcriptomic

programs of primary and iPSC-derived human alveolar type 2 cells. JCI Insight *8*, e158937.

Barkauskas, C.E., Cronce, M.J., Rackley, C.R., Bowie, E.J., Keene, D.R., Stripp, B.R., Randell, S.H., Noble, P.W., and Hogan, B.L.M. (2013). Type 2 alveolar cells are stem cells in adult lung. J. Clin. Invest. *123*, 3025–3036.

Borok, Z., Danto, S.I., Lubman, R.L., Cao, Y., Williams, M.C., and Crandall, E.D. (1998). Modulation of t1alpha expression with alveolar epithelial cell phenotype in vitro. Am. J. Physiol. *275*, L155–L164.

Burgess, C.L., Huang, J., Bawa, P., Alysandratos, K.-D., Minakin, K., Morley, M.P., Babu, A., Villacorta-Martin, C., Hinds, A., Thapa, B.R., et al. (2023). Generation of human alveolar epithelial type I cells from pluripotent stem cells. BioRxiv. https://doi.org/10.1101/2023.01.19.524655.

Choi, J., Park, J.-E., Tsagkogeorga, G., Yanagita, M., Koo, B.-K., Han, N., and Lee, J.-H. (2020). Inflammatory Signals Induce AT2 Cell-Derived Damage-Associated Transient Progenitors that Mediate Alveolar Regeneration. Cell Stem Cell *27*, 366–382.e7.

Chung, M.-I., and Hogan, B.L.M. (2018). Ager-CreERT2: A New Genetic Tool for Studying Lung Alveolar Development, Homeostasis, and Repair. Am. J. Respir. Cell Mol. Biol. *59*, 706–712.

Coffey, J.C., Wang, J.H., Smith, M.J.F., Laing, A., Bouchier-Hayes, D., Cotter, T.G., and Redmond, H.P. (2005). Phosphoinositide 3-kinase accelerates postoperative tumor growth by inhibiting apoptosis and enhancing resistance to chemotherapy-induced apoptosis. Novel role for an old enemy. J. Biol. Chem. *280*, 20968–20977.

Dahlin, K., Mager, E.M., Allen, L., Tigue, Z., Goodglick, L., Wadehra, M., and Dobbs, L. (2004). Identification of genes differentially expressed in rat alveolar type I cells. Am. J. Respir. Cell Mol. Biol. *31*, 309–316.

Desai, T.J., Brownfield, D.G., and Krasnow, M.A. (2014). Alveolar progenitor and stem cells in lung development, renewal and cancer. Nature *507*, 190–194.

DiGiovanni, G.T., Han, W., Sherrill, T.P., Taylor, C.J., Nichols, D.S., Geis, N.M., Singha, U.K., Calvi, C.L., McCall, A.S., Dixon, M.M., et al. (2023). Epithelial Yap/Taz are required for functional alveolar regeneration following acute lung injury. JCI Insight *8*, e173374. https://doi.org/10.1172/jci.insight.173374.

Finn, J., Sottoriva, K., Pajcini, K.V., Kitajewski, J.K., Chen, C., Zhang, W., Malik, A.B., and Liu, Y. (2019). Dlk1-Mediated Temporal Regulation of Notch Signaling Is Required for Differentiation of Alveolar Type II to Type I Cells during Repair. Cell Rep. *26*, 2942–2954.e5.

Frank, D.B., Peng, T., Zepp, J.A., Snitow, M., Vincent, T.L., Penkala, I.J., Cui, Z., Herriges, M.J., Morley, M.P., Zhou, S., et al. (2016). Emergence of a Wave of Wnt Signaling that Regulates Lung Alveologenesis by Controlling Epithelial Self-Renewal and Differentiation. Cell Rep. *17*, 2312–2325.

Gokey, J.J., Sridharan, A., Xu, Y., Green, J., Carraro, G., Stripp, B.R., Perl, A.-K.T., and Whitsett, J.A. (2018). Active epithelial Hippo signaling in idiopathic pulmonary fibrosis. JCI Insight *3*, e98738. https://doi.org/10.1172/jci.insight.98738.

Gokey, J.J., Snowball, J., Sridharan, A., Sudha, P., Kitzmiller, J.A., Xu, Y., and Whitsett, J.A. (2021). YAP regulates alveolar epithelial cell differentiation and AGER via NFIB/KLF5/NKX2-1. iScience *24*, 102967.

Gotoh, S., Ito, I., Nagasaki, T., Yamamoto, Y., Konishi, S., Korogi, Y., Matsumoto, H., Muro, S., Hirai, T., Funato, M., et al. (2014). Generation of alveolar epithelial spheroids via isolated progenitor cells from human pluripotent stem cells. Stem Cell Rep. *3*, 394–403.

Guo, M., Morley, M.P., Jiang, C., Wu, Y., Li, G., Du, Y., Zhao, S., Wagner, A., Cakar, A.C., Kouril, M., et al. (2023). Guided construction of single cell reference for human and mouse lung. Nat. Commun. *14*, 4566.

Hekman, R.M., Hume, A.J., Goel, R.K., Abo, K.M., Huang, J., Blum, B.C., Werder, R.B., Suder, E.L., Paul, I., Phanse, S., et al. (2020). Actionable Cytopathogenic Host Responses of Human Alveolar Type 2 Cells to SARS-CoV-2. Mol. Cell *80*, 1104–1122.e9.

Kaiser, A.M., Gatto, A., Hanson, K.J., Zhao, R.L., Raj, N., Ozawa, M.G., Seoane, J.A., Bieging-Rolett, K.T., Wang, M., Li, I., et al. (2023). p53 governs an AT1 differentiation programme in lung cancer suppression. Nature *619*, 851–859.

Kanagaki, S., Ikeo, S., Suezawa, T., Yamamoto, Y., Seki, M., Hirai, T., Hagiwara, M., Suzuki, Y., and Gotoh, S. (2021). Directed induction of alveolar type I cells derived from pluripotent stem cells via Wnt signaling inhibition. Stem Cell. *39*, 156–169.

Kastan, N., Gnedeva, K., Alisch, T., Petelski, A.A., Huggins, D.J., Chiaravalli, J., Aharanov, A., Shakked, A., Tzahor, E., Nagiel, A., et al. (2021). Small-molecule inhibition of Lats kinases may promote Yap-dependent proliferation in postmitotic mammalian tissues. Nat. Commun. *12*, 3100.

Kawase, T., Ohki, R., Shibata, T., Tsutsumi, S., Kamimura, N., Inazawa, J., Ohta, T., Ichikawa, H., Aburatani, H., Tashiro, F., and Taya, Y. (2009). PH domain-only protein PHLDA3 is a p53-regulated repressor of Akt. Cell *136*, 535–550.

Kobayashi, Y., Tata, A., Konkimalla, A., Katsura, H., Lee, R.F., Ou, J., Banovich, N.E., Kropski, J.A., and Tata, P.R. (2020). Persistence of a regeneration-associated, transitional alveolar epithelial cell state in pulmonary fibrosis. Nat. Cell Biol. *22*, 934–946.

LaCanna, R., Liccardo, D., Zhang, P., Tragesser, L., Wang, Y., Cao, T., Chapman, H.A., Morrisey, E.E., Shen, H., Koch, W.J., et al. (2019). Yap/Taz regulate alveolar regeneration and resolution of lung inflammation. J. Clin. Invest. *129*, 2107–2122.

Liu, S., Sun, D., Butler, R., and Rawlins, E.L. (2023). RTK signalling promotes epithelial columnar cell shape and apical junction maintenance in human lung progenitor cells. Development *150*, dev201284. https://doi.org/10.1242/dev.201284.

Liu, Z., Wu, H., Jiang, K., Wang, Y., Zhang, W., Chu, Q., Li, J., Huang, H., Cai, T., Ji, H., et al. (2016). MAPK-Mediated YAP Activation Controls Mechanical-Tension-Induced Pulmonary Alveolar Regeneration. Cell Rep. *16*, 1810–1819.

Manning, B.D., and Toker, A. (2017). AKT/PKB Signaling: Navigating the Network. Cell *169*, 381–405.

Nabhan, A.N., Brownfield, D.G., Harbury, P.B., Krasnow, M.A., and Desai, T.J. (2018). Single-cell Wnt signaling niches maintain stemness of alveolar type 2 cells. Science *359*, 1118–1123.

Nguyen, T.M., van der Merwe, J., Elowsson Rendin, L., Larsson-Callerfelt, A.-K., Deprest, J., Westergren-Thorsson, G., and Toelen, J. (2021). Stretch increases alveolar type 1 cell number in fetal lungs through ROCK-Yap/Taz pathway. Am. J. Physiol. Lung Cell Mol. Physiol. *321*, L814–L826. https://doi.org/10.1152/ajplung.00484.2020.

Penkala, I.J., Liberti, D.C., Pankin, J., Sivakumar, A., Kremp, M.M., Jayachandran, S., Katzen, J., Leach, J.P., Windmueller, R., Stolz, K., et al. (2021). Age-dependent alveolar epithelial plasticity orchestrates lung homeostasis and regeneration. Cell Stem Cell *28*, 1775–1789.e5.

Portnoy, J., Curran-Everett, D., and Mason, R.J. (2004). Keratinocyte growth factor stimulates alveolar type II cell proliferation through the extracellular signal-regulated kinase and phosphatidylinositol 3-OH kinase pathways. Am. J. Respir. Cell Mol. Biol. *30*, 901–907.

Shiraishi, K., Shah, P.P., Morley, M.P., Loebel, C., Santini, G.T., Katzen, J., Basil, M.C., Lin, S.M., Planer, J.D., Cantu, E., et al. (2023). Biophysical forces mediated by respiration maintain lung alveolar epithelial cell fate. Cell *186*, 1478–1492.e15.

Strunz, M., Simon, L.M., Ansari, M., Kathiriya, J.J., Angelidis, I., Mayr, C.H., Tsidiridis, G., Lange, M., Mattner, L.F., Yee, M., et al. (2020). Alveolar regeneration through a Krt8+ transitional stem cell state that persists in human lung fibrosis. Nat. Commun. *11*, 3559.

Tamai, K., Sakai, K., Yamaki, H., Moriguchi, K., Igura, K., Maehana, S., Suezawa, T., Takehara, K., Hagiwara, M., Hirai, T., and Gotoh, S. (2022). iPSC-derived mesenchymal cells that support alveolar organoid development. Cell Rep. Methods *2*, 100314.

Travaglini, K.J., Nabhan, A.N., Penland, L., Sinha, R., Gillich, A., Sit, R.V., Chang, S., Conley, S.D., Mori, Y., Seita, J., et al. (2020). A molecular cell atlas of the human lung from single-cell RNA sequencing. Nature *587*, 619–625.

Warren, R., Lyu, H., Klinkhammer, K., and De Langhe, S.P. (2023). Hippo signaling impairs alveolar epithelial regeneration in pulmonary fibrosis. Elife *12*, e85092. https://doi.org/10.7554/eLife.85092.

Xu, Y., Mizuno, T., Sridharan, A., Du, Y., Guo, M., Tang, J., Wikenheiser-Brokamp, K.A., Perl, A.-K.T., Funari, V.A., Gokey, J.J., et al. (2016). Single-cell RNA sequencing identifies diverse roles of epithelial cells in idiopathic pulmonary fibrosis. JCI Insight *1*, e90558.

Yamamoto, Y., Gotoh, S., Korogi, Y., Seki, M., Konishi, S., Ikeo, S., Sone, N., Nagasaki, T., Matsumoto, H., Muro, S., et al. (2017). Long-term expansion of alveolar stem cells derived from human iPS cells in organoids. Nat. Methods *14*, 1097–1106.

Zepp, J.A., Morley, M.P., Loebel, C., Kremp, M.M., Chaudhry, F.N., Basil, M.C., Leach, J.P., Liberti, D.C., Niethamer, T.K., Ying, Y., et al. (2021). Genomic, epigenomic, and biophysical cues controlling the emergence of the lung alveolus. Science *371*, eabc3172. https://doi.org/10.1126/science.abc3172.

Zhang, J., Zhou, J., Yu, Y., Cai, Y., Li, Z., Lu, Y., and Zhao, J. (2023). Sesamin Induces the Transdifferentiation of Type II Alveolar Epithelial Cells via AnnexinA1 and TRPV1. Lung *201*, 65–77.

Zhao, L., Yee, M., and O'Reilly, M.A. (2013). Transdifferentiation of alveolar epithelial type II to type I cells is controlled by opposing TGF-β and BMP signaling. Am. J. Physiol. Lung Cell Mol. Physiol. *305*, L409–L418.

Zhong, Q., Liu, Y., Correa, M.R., Marconett, C.N., Minoo, P., Li, C., Ann, D.K., Zhou, B., and Borok, Z. (2022). FOXO1 Couples KGF and PI-3K/AKT Signaling to NKX2.1-Regulated Differentiation of Alveolar Epithelial Cells. Cells *11*, 1122. https://doi.org/10.3390/cells11071122.

Stem Cell Reports, Volume *19*

Supplemental Information

Screening of factors inducing alveolar type 1 epithelial cells using human pluripotent stem cells

Yuko Ohnishi, Atsushi Masui, Takahiro Suezawa, Ryuta Mikawa, Toyohiro Hirai, Masatoshi Hagiwara, and Shimpei Gotoh

Supplemental information

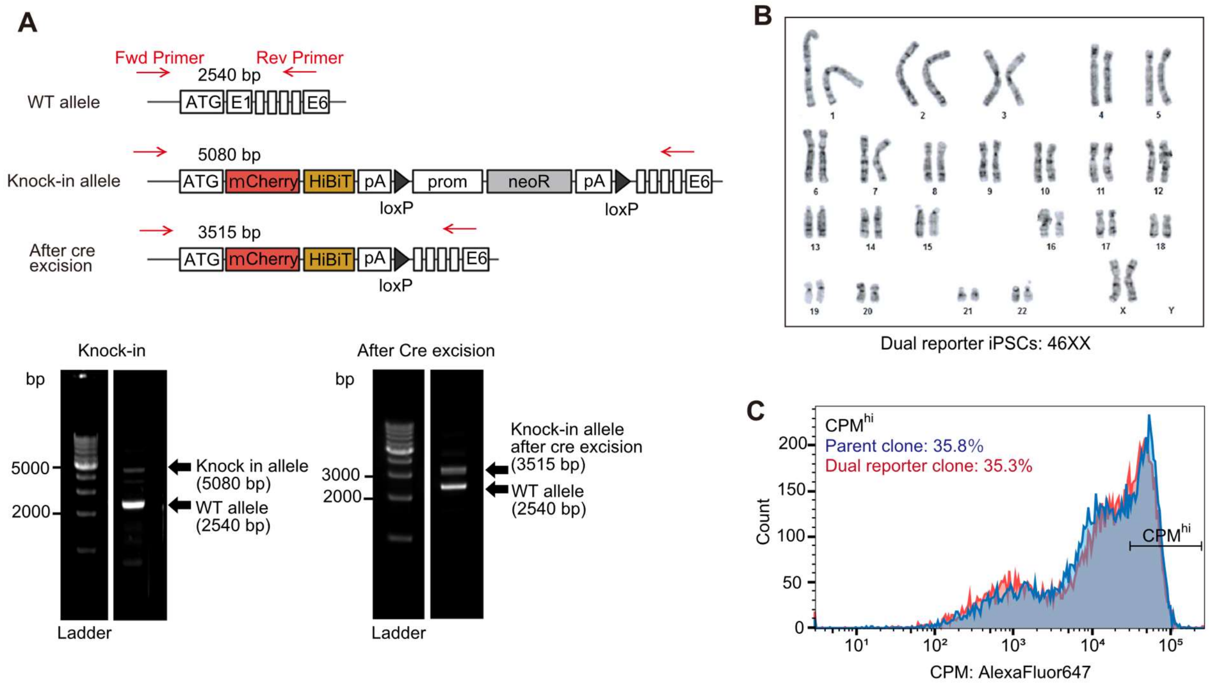

Figure S1. Validation of the *SFTPC^{GFP} AGER$^{mCherry\text{-}HiBiT}$* dual-reporter iPSC line. Related to Figure 1.

A. Schematic diagram of PCR confirming the successful genome editing and the result of electrophoresis of PCR products. Red arrows represent the forward and reverse primers.
B. Karyotype of the dual reporter iPSC line (B2-3-dual-42-7).
C. Flow cytometry analysis of CPM^{hi} lung epithelial progenitor cells differentiated from the dual reporter iPSCs and its parental clone.

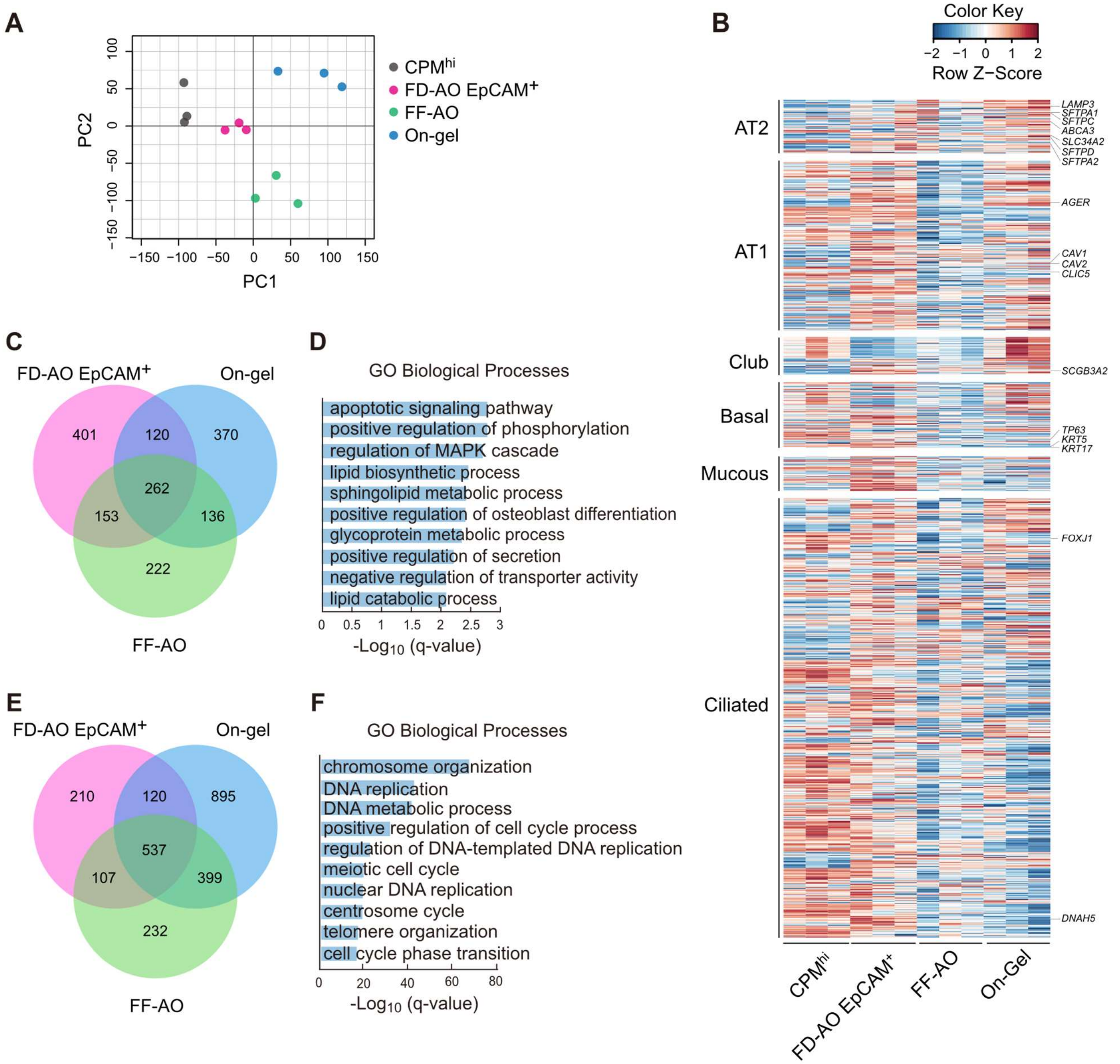

Figure S2. Characterization of the on-gel culture method. Related to Figure 2.

A. PCA of CPM^{hi} progenitor cells and P1 GFP^{+} iAT2 cell-derived epithelial cells using the three culture methods. Log_2 (TPM values) were used for analysis. Data were obtained from three independent experiments.

B. A heatmap of the transcriptomes of iPSC-derived lung epithelial cells was created with a value of log_2 (TPM + 0.01) using a gene set from the previous study[1].

C. Venn diagram of DEGs that were upregulated in epithelial cells from the three culture methods compared with CPM^{hi} cells. The threshold for upregulation was set to log_2 (fold-change) > 1, with an adjusted P-value of < 0.01.

D. Enrichment analysis of 370 genes specifically upregulated in on-gel alveolar epithelial spheroids.

E. Venn diagram of DEGs that were downregulated in epithelial cells from the three culture methods compared with CPM^{hi} cells. The threshold of downregulation was set to log_2 (fold change) < −1 with an adjusted P-value of < 0.01.

F. Enrichment analysis of 895 genes specifically downregulated in on-gel alveolar epithelial spheroids.

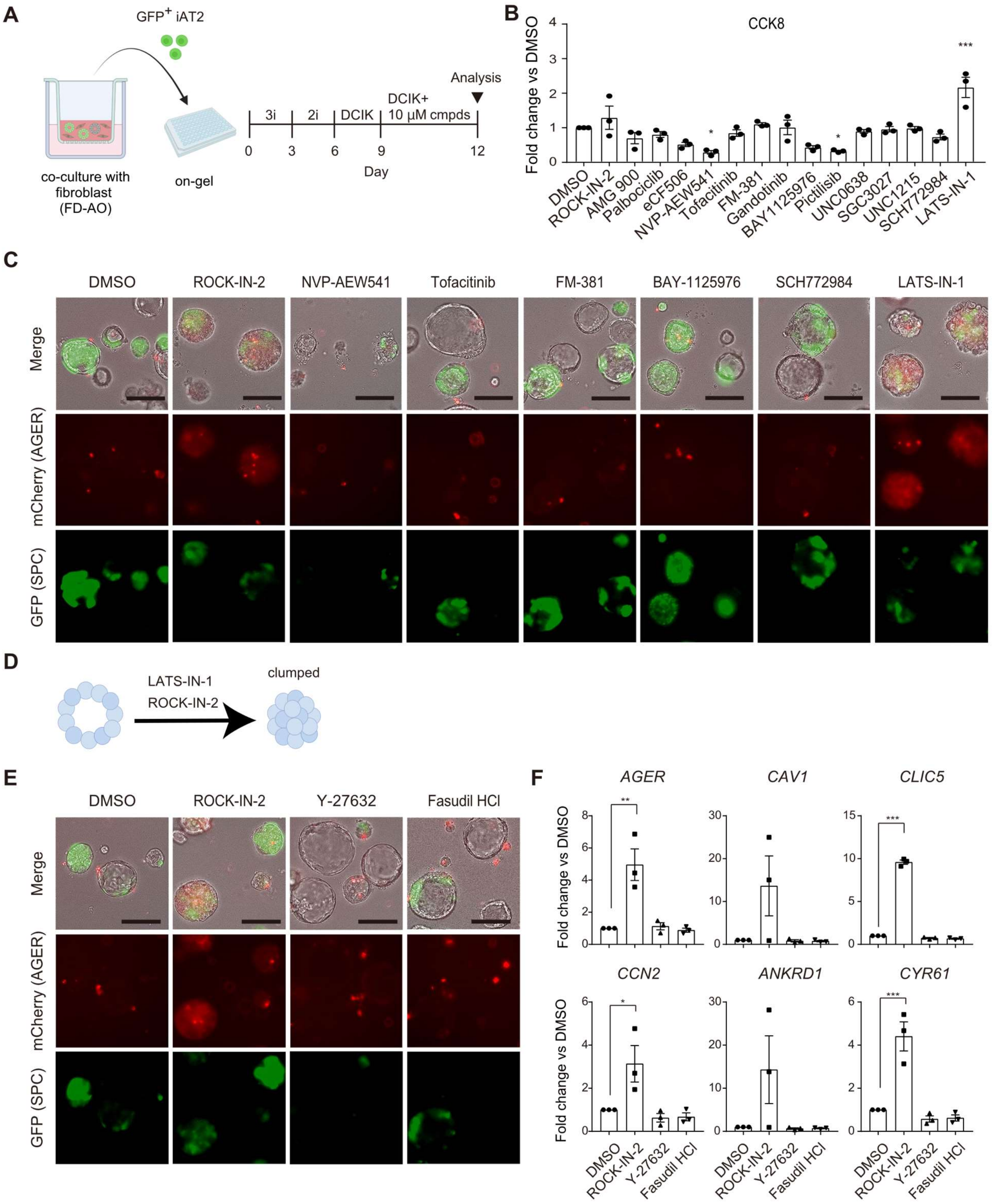
A
GFP+ iAT2
co-culture with fibroblast (FD-AO)
on-gel
3i
2i
DCIK
DCIK+ 10 µM cmpds
Analysis
0
3
6
9
12
Day
B
CCK8
Fold change vs DMSO
DMSO
ROCK-IN-2
AMG 900
Palbociclib
eCF506
NVP-AEW541
Tofacitinib
FM-381
Gandotinib
BAY1125976
Pictilisib
UNC0638
SGC3027
UNC1215
SCH772984
LATS-IN-1
C
DMSO
ROCK-IN-2
NVP-AEW541
Tofacitinib
FM-381
BAY-1125976
SCH772984
LATS-IN-1
Merge
mCherry (AGER)
GFP (SPC)
D
LATS-IN-1
ROCK-IN-2
clumped
E
DMSO
ROCK-IN-2
Y-27632
Fasudil HCl
Merge
mCherry (AGER)
GFP (SPC)
F
AGER
CAV1
CLIC5
CCN2
ANKRD1
CYR61
Fold change vs DMSO
DMSO
ROCK-IN-2
Y-27632
Fasudil HCl

Figure S3. Validation of screening hit compounds with GFP$^+$ iAT2 cell-derived spheroids on-gel. Related to Figure 3.

A. Schematic illustration of the strategy for the secondary evaluation of the hit compounds using GFP$^+$ iAT2 cell-derived on-gel alveolar epithelial spheroids.
B. CCK8 assay of on-gel iAT2 cell-derived spheroids treated with the compounds. Data are shown as mean ± SEM (n = 3 from independent experiments). One-way ANOVA with Dunnett's multiple comparison test: *P < 0.05, ***P < 0.005.
C. Live cell imaging of on-gel iAT2 cell-derived spheroids treated with each compound. Scale bars = 100 μm.
D. Illustration of the morphological change following treatment with LATS-IN-1 or ROCK-IN-2.
E. Live cell imaging of on-gel iAT2 cell-derived spheroids treated with each compound. Scale bars = 100 μm.
F. mRNA expression of GFP$^+$ iAT2 cell-derived spheroids treated with 10 μM of each ROCK inhibitor. Data are shown as mean ± SEM (n = 3 from independent experiments). One-way ANOVA with Dunnett's multiple comparison test: *P < 0.05, **P < 0.01, ***P < 0.005.

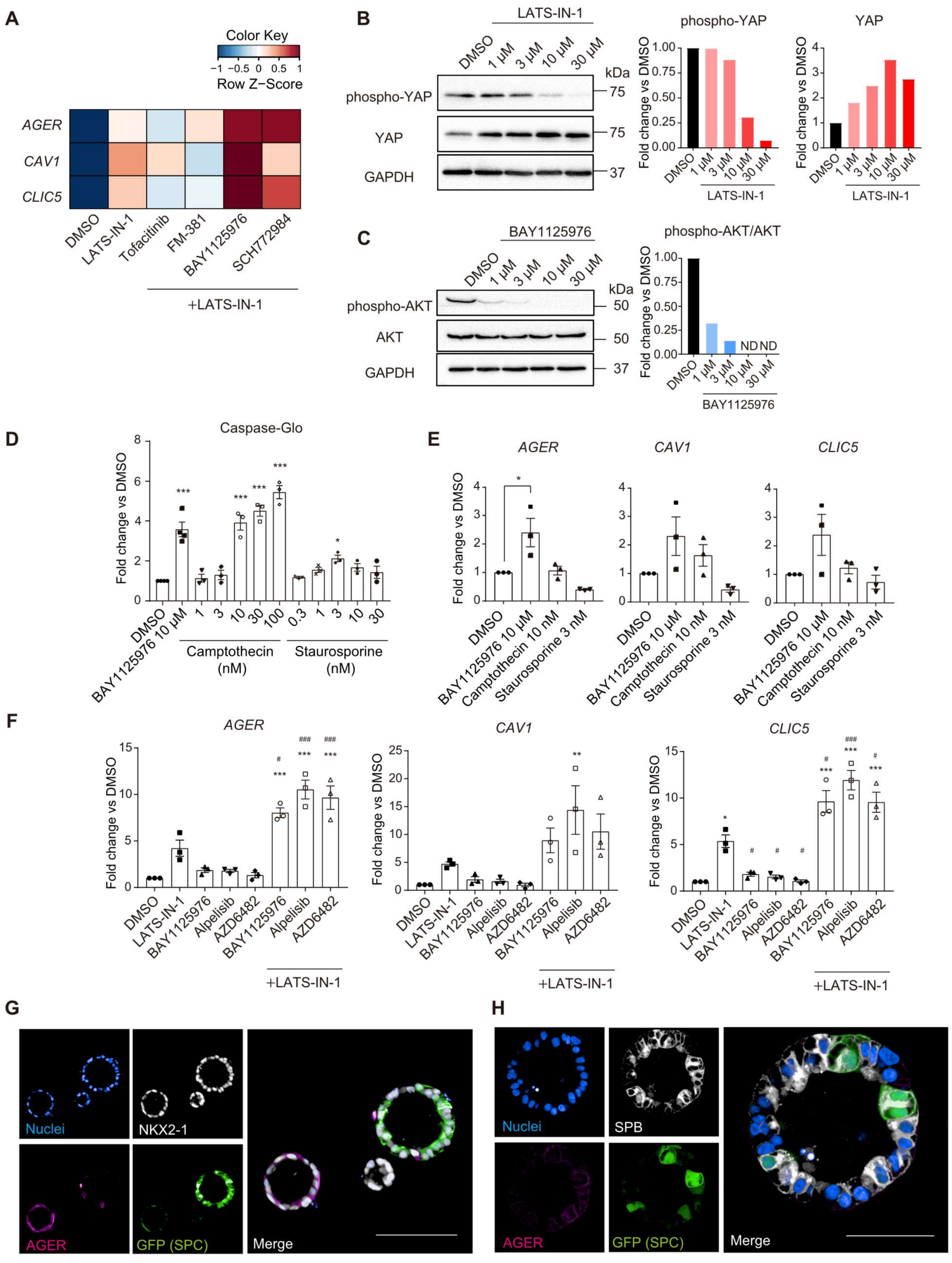
A
Color Key
Row Z−Score
AGER
CAV1
CLIC5
DMSO
LATS-IN-1
Tofacitinib
FM-381
BAY1125976
SCH772984
+LATS-IN-1
B
LATS-IN-1
DMSO 1 μM 3 μM 10 μM 30 μM
kDa
phospho-YAP
YAP
GAPDH
phospho-YAP
YAP
Fold change vs DMSO
C
BAY1125976
phospho-AKT
AKT
GAPDH
phospho-AKT/AKT
ND ND
D
Caspase-Glo
Fold change vs DMSO
BAY1125976 10 μM
Camptothecin (nM)
Staurosporine (nM)
E
AGER
CAV1
CLIC5
Camptothecin 10 nM
Staurosporine 3 nM
F
AGER
CAV1
CLIC5
Alpelisib
AZD6482
+LATS-IN-1
G
Nuclei
NKX2-1
AGER
GFP (SPC)
Merge
H
Nuclei
SPB
AGER
GFP (SPC)
Merge

Figure S4. PI3K/AKT signaling involvement in AT1 cell differentiation. Related to Figure 4.

A. AT1 cell marker gene expression in the on-gel alveolar epithelial spheroids using GFP^+ iAT2 cells derived from dual-reporter iPSCs (P1). On-gel spheroids were treated with 10 μM of each compound. Data are shown as the mean of the $\log_2$ (ratio to adult lung) (n = 3 from independent experiments).

B. Western blotting of the on-gel alveolar epithelial spheroids using $SFTPC^{GFP}$ reporter iPSC-derived CPM^{hi} lung progenitor cells. Spheroids were treated with LATS-IN-1. Phospho (S127) -YAP and total YAP bands were quantified and normalized by the levels of GAPDH.

C. Western blotting of the on-gel alveolar epithelial spheroids using $SFTPC^{GFP}$ reporter iPSC-derived CPM^{hi} lung progenitor cells. Spheroids were treated with BAY1125976. Phospho (Ser473) -AKT and total AKT bands were quantified and phosphor-AKT bands were normalized to total AKT bands. ND, Not Detected.

D. Caspase-Glo assay to quantify the apoptosis of GFP^+ iAT2 cells derived from $SFTPC^{GFP}$ $AGER^{mCherry\text{-}HiBiT}$ dual-reporter iPSCs (P1) affected by BAY1125976 and different concentrations of apoptosis inducers, Camptothecin and Staurosporine. Data are shown as mean ± SEM (n = 3 from independent experiments). One-way ANOVA with Dunnett's multiple comparison test: *P < 0.05, ***P < 0.005.

E. AT1 cell marker gene expression in the on-gel alveolar spheroids using GFP^+ iAT2 cells derived from the dual-reporter iPSCs (P1). Each compound was applied at arbitrary concentrations. Data are shown as mean ± SEM (n = 3 from independent experiments). One-way ANOVA with Dunnett's multiple comparison test: *P < 0.05, ***P < 0.005.

F. AT1 cell marker gene expression in the dual-reporter GFP^+ iAT2 cell-derived on-gel spheroids (P1). Each column represents samples treated with 10 μM of each compound alone or a combination of PI3K/AKT inhibitors and LATS-IN-1. Data are shown as mean ± SEM (n = 3 from independent experiments). One-way ANOVA with Tukey's multiple comparison test was used for analysis. Results are shown only in comparison with the DMSO-treated group: *P < 0.05, **P < 0.01, ***P < 0.005, and the LATS-IN-1-treated group: #P < 0.05, ###P < 0.005.

G, H. Whole mount immunofluorescence imaging of on-gel spheroids derived from GFP^+ iAT2 using $SFTPC^{GFP}$ reporter iPSCs treated with DMSO. Scale bars = 100 and 50 μm respectively.

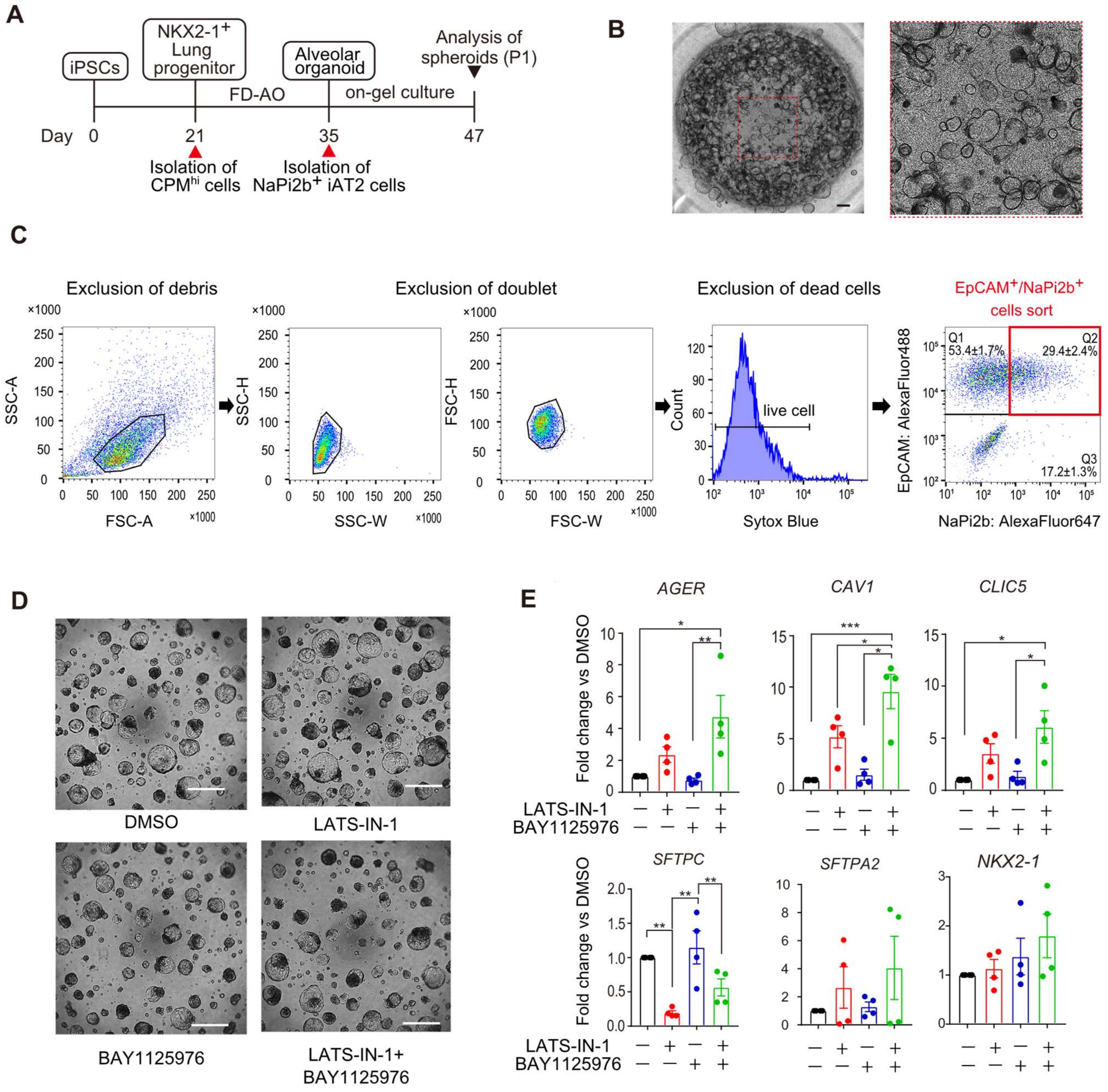

Figure S5. Validation of synergistic effect of YAP/TAZ activation and AKT suppression using ChiPSC18 line. Related to Figure 4.

A. Schematic diagram of the differentiation of the ChiPSC18 line into iAT2 cells followed by on-gel culture.
B. Live-cell imaging of FD-AOs derived from ChiPSC18 line. Scale bar = 500 μm.
C. Flow cytometry gating strategy for isolating iAT2 cells from FD-AOs derived from ChiPSC18 cells. The rate of each gated cell is shown as mean ± SEM (n = 3 from independent experiments).
D. Live-cell imaging of the compound-treated on-gel spheroids derived from iAT2 cells. Scale bars = 500 μm.
E. mRNA expression in on-gel iAT2 cell-derived spheroids (P1). Each column represents samples treated with 10 μM LATS-IN-1, 10 μM BAY1125976, or both. Data are shown as mean ± SEM (n = 4 from independent experiments). One-way ANOVA with Tukey's multiple comparisons test: *P < 0.05, **P < 0.01, ***P < 0.005.

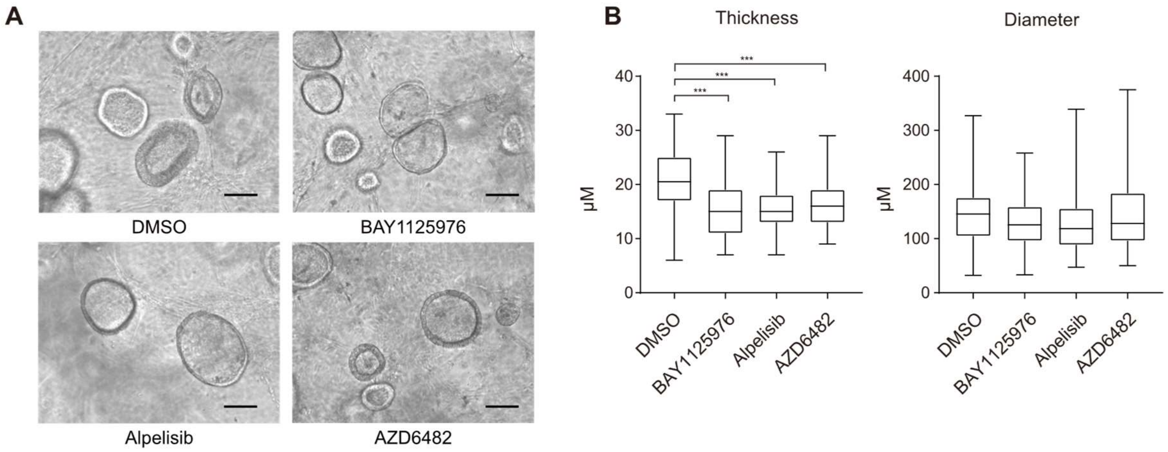

Figure S6. Morphological changes of FD-AOs induced by PI3K/AKT signaling inhibitors. Related to Figure 7.

A. Live cell imaging of FD-AOs (P2) treated with the indicated compounds. Scale bars = 100 μm.
B. Quantification of the organoid thickness and diameter. Data are shown as mean ± SEM (n = 60 from three independent experiments. A total of 20 organoids were randomly selected from each experiment). One-way ANOVA with Dunnett's multiple comparison test: ***P < 0.005.

Table S2: List of antibodies used in this study

Epitope	Host	Supplier	Catalog No.	Dilution (IF)	Dilution (FACS, MACS)	Dilution (Immunoblot)
HT2-280	Mouse	Terrace Biotech	SKU: TB-27AHT2-280		1:100	
NaPi2b	Mouse	kindly provided by Dr. Gerd Ritter (MX35)		1:100	1:100	
mCherry	Chicken	Abcam	ab205402	1:500		
GFP	Chicken	Abes labs	GFP -1020	1:500		
GFP	Rabbit	Cell Signaling Technology	2956	1:200		
EpCAM	Goat	R&D systems	AF960		1:100	
EpCAM	Mouse	Santa Cruz Biotechnology	sc-66020		1:100	
EpCAM-APC	Mouse	Milteny Biotec	130-113-260		1:100	
AGER	Goat	R&D systems	AF1145	1:100		
HT1-56	Mouse	Terrace Biotech	SKU: TB-29AHT1-56	1:150		
CPM	Mouse	Fujifilm Wako	014-27501		1:100	
SPB	Mouse	Santa Cruz	133143	1:50		
NKX2-1	Rabbit	Abcam	76013	1:100		
Phospho-YAP	Rabbit	Cell Signaling Technology	13008			1:1000
YAP	Mouse	Santa Cruz	101199			1:400
Phospho-AKT	Rabbit	Cell Signaling Technology	4060			1:1000
AKT	Rabbit	Cell Signaling Technology	9272			1:1000
GAPDH-HRP	Mouse	Fujifilm Wako	015-25473			1:3000
Chicken IgY-Cy3	Donkey	Jackson laboratory	703-165-155	1:500		
Chicken IgY-Alexa Fluor 488	Donkey	Jackson laboratory	703 -485-155	1:500		
Goat IgG-Alexa Fluor 488	Donkey	ThermoFisher Scientific	A11055		1:100	
Goat IgG-Alexa Fluor 647	Donkey	ThermoFisher Scientific	A21447	1:500		
Mouse IgM-Alexa Fluor 647	Donkey	Jackson laboratory	715-605-140		1:100	

Mouse IgG-Alexa Fluor 647	Donkey	ThermoFisher Scientific	A31571		1:100	
Mouse IgG-Alexa Fluor 488	Donkey	ThermoFisher Scientific	A21202	1:500		
Mouse IgG-Alexa Fluor 546	Donkey	ThermoFisher Scientific	A10036	1:500		
Rabbit IgG-Alexa Fluor 488	Donkey	ThermoFisher Scientific	A21206	1:500		
Mouse IgG-HRP	Horse	Cell Signaling Technology	7076			1:5000
Rabbit IgG-HRP	Horse	Cell Signaling Technology	7074			1:5000

Table S3. Primers used for TaqMan qPT-PCR

Gene name	TaqMan ID
18S	Hs99999901_s1
AGER	Hs00542584_g1
SFTPC	Hs00161628_m1

Table S4. Primers used for SYBR qPT-PCR

Gene name	Forward primer	Reverse primer
18S	TTGACGGAAGGGCACCACCAG	GCACCACCACCCACGGAATCG
CAV1	AGGGCAACATCTACAAGCCC	GCCGTCAAAACTGTGTGTCC
CLIC5	CTATGATATCCCGGCTGAGATGAC	CACGGGCATAGGCGTTCTT
GPRC5A	AGACAGGGGACACGCTCTAT	GGAGGCAAACTGTTCCCGTA
NKX2-1	AGCACACGACTCCGTTCTC	GCCCACTTTCTTGTAGCTTTCC
mCherry	AAGGCCTACGTGAAGCACCC	ATCACGCGCTCCCACTTGAA
SFTPA2	AAGCAGCTGGAGGCTCTGT	CCATCAAGATGAGGGTGAGG
LAMP3	ACCGATGTCCAACTTCAAGC	TGACACCTTAGGCGGATTTT

Table S5. Compounds related to Figure 3C, S3, S4

Compound	Cat.	Supplier
ROCK-IN-2	S6636	Selleck Biotech
AMG 900	HY-13253	MedChemExpress
Palbociclib	HY-50767A	MedChemExpress
eCF506	HY-112096	MedChemExpress
NVP-AEW541	HY-50866	MedChemExpress
Tofacitinib	S2789	Selleck Biotech
FM-381	HY-102046	MedChemExpress
Gandotinib	HY-13034	MedChemExpress
BAY-1125976	HY-100018	MedChemExpress

Pictilisib	HY-50094	MedChemExpress
UNC0638	HY-15273	MedChemExpress
SGC3027	HY-112445	MedChemExpress
UNC1215	13968	Cayman CHEMICAL
SCH772984	HY-50846	MedChemExpress
LATS-IN-1	E1061	Selleck Biotech
Fasudil HCl	S1573	Selleck Biotech
Y-27632	HY-10071	MedChemExpress
Alpelisib	HY-15244	MedChemExpress
AZD6482	HY-10344	MedChemExpress
Camptothecin	038-18191	Fujifilm Wako
Staurosporine	197-10251	Fujifilm Wako

SUPPLEMENTARY METHODS

Generation of *SFTPC^{GFP} AGER$^{mCherry\text{-}HiBiT}$* dual-reporter iPSC

A guide RNA (gRNA) was designed to cleave downstream of the *AGER* start codon (gRNA sequence: 5`-ATGGCAGCCGGAACAGCAGT-3`). gRNA was synthesized (Macrogen) and inserted into pHL-H1-ccdB-mEF1a-RIH, which was a gift from Dr. Akitsu Hotta (Addgene plasmid # 6060). A donor vector carrying the *mCherry-HiBiT* reporter gene was designed and synthesized (Vector Builder). Before genome editing, the *SFTPC^{GFP}* reporter iPSC line (B2-3) was maintained on an iMatrix-511 (Takara Bio T311)-coated plate in StemFit AK02N medium (Ajinomoto AK02N) supplemented with penicillin-streptomycin (P–S; Thermo Fisher Scientific 15140122). Cells were treated with Y-27632 (LC Laboratories) at 10 μM for at least 1 h before electroporation. After washing cells with PBS, cells were dissociated into single cells with Accutase (Innovative Cell Technologies ICT-AT104-500-500) for 20 min under 37°C and collected with StemFit AK02N supplemented with 10 μM Y-27632. 5 μg of sgRNA vector, 5 μg of donor vector, and 5 μg of pHL-EF1a-SphcCas9-iP-A, a gift from Dr. Akitsu Hotta (Addgene plasmid # 60599) (Li et al., 2015), were electroporated into 1×10^6 cells suspended in Opti-MEM (ThermoFisher Scientific 31985062) using NEPA21 electroporator (Nepa Gene). The cells were re-seeded on an iMatrix-511-coated plate in StemFit AK02N supplemented with 10 μM Y-27632. 2 days after electroporation, Y-27632 was removed. 6 days after electroporation, the cells were cultured with StemFit AK02N supplemented with 100 μg/mL G418 (Gibco 10131035) for 6 days to select the transfected cells. Cells were dissociated and re-seeded at limiting dilutions of 200, 500, and 1,500 cells in a 10 cm dish coated with iMatrix-511 in StemFit AK02N and cultured for 7-10 days. Single clones were screened using genomic PCR and Sanger sequencing to obtain positive clones with the knock-in of the reporter gene into a single allele. A total of 10 μg of pCXW-Cre-Puro (a kind gift from Dr. Keisuke Okita) was electroporated into two positive clones, and the cells were re-seeded at limiting dilutions. Single clones were checked for excision of the G418 resistance gene cassette by genomic PCR and Sanger sequencing, and a dual-reporter iPSC clone (B2-3-dual-42-7) was selected for subsequent experiments. Karyotyping of the dual-reporter iPSC (B2-3-dual-42-7) was performed using G-banding (Nihon Gene Research Laboratories Inc, Japan).

Culture of human iPSC and fetal lung fibroblasts

Human iPSCs were cultured, as described previously (Yamamoto et al., 2017). Briefly, the *SFTPC^{GFP}* reporter iPSC line (B2-3), *SFTPC^{GFP} AGER$^{mCherry\text{-}HiBiT}$* dual-reporter iPSC line (B2-3-dual-42-7), and ChiPSC18 (Takara-bio Y00305) cell line were cultured on Geltrex (Thermo Fisher Scientific A1413202)-coated plates in Essential 8 medium (Thermo Fisher Scientific A1517001) without feeder cells. Human fetal lung fibroblasts (HFLF) (17.5 weeks of gestation; DV Biologics #PP002-F-1349, lot 121109VA) were cultured in Dulbecco's Modified Eagle's medium (Nacalai Tesque 08459-64) supplemented with 50 U/mL P–S and 10% fetal bovine serum (Sigma Aldrich SF7524).

Differentiation of iPSC into NKX2.1+ lung epithelial progenitor cells

Human iPSCs were differentiated into lung epithelial progenitor cells as previously describe[2]. Briefly, each iPSC clone was seeded on a Geltrex-coated plate and cultured in RPMI medium (Nacalai Tesque 30264-56)

supplemented with Activin A (API GF-001-050L), 1 μM CHIR99021 (Axon Medchem), 10 μM Y-27632, 2% B-27 supplement (Thermo Fisher Scientific 17504001), and 50 U/mL P–S. One day after seeding, sodium butyrate was added at a final concentration of 0.25 mM. From day 2 to 5, the cells were cultured in RPMI medium supplemented with activin, 1 μM CHIR99021, 0.125 mM sodium butyrate, 2% B-27 supplement, and 50 U/mL P–S. After day 6, the cell culture medium was formulated based on DMEM/F12 (Thermo Fisher Scientific 11320033) supplemented with Glutamax (Thermo Fisher Scientific 35050-061), 2% B27 supplement, 0.05 mg/mL L-ascorbic acid (Wako 016-04805), 0.4 mM monothioglycerol (Wako 195-15791), and 50 U/mL P–S. From day 6 to 10, the cells were cultured in a basal medium containing 100 ng/mL Noggin (R&D systems 6057-NG-01M) and 10 μM SB431542 (Fujifilm Wako 198-16543) to differentiate into anterior foregut endoderm (AFE) cells. From the day 10 to 14, the medium was changed to basal medium supplemented with 3 μM CHIR99021, 0.05 μM all-trans retinoic acid (Sigma-Aldrich R2625), and 20 ng/mL BMP4 (Peprotech 120-05ET) for B2-3 and B2-3-dual-42-7 iPSC line, 3.5 μM CHIR99021, 1 μM all-trans retinoic acid, and 20 ng/mL BMP4 for ChiPSC18 line for differentiation into ventralized anterior foregut endoderm (VAFE) cells. From day 14 onward, cells were cultured in the basal medium supplemented with 3 μM CHIR99021, 10 ng/mL FGF10 (PeproTech 100-26), 10 ng/mL KGF (PeproTech AF-100-19), and 20 μM DAPT (Fujifilm Wako 049-33583) to differentiate into $NKX2.1^{+}$ lung progenitor cells. On day 21, CPM^{hi} cells were labeled with mouse anti-human CPM antibody (Fujifilm Wako 014-27501) and isolated by FACS using AlexaFluor647-conjugated donkey anti-mouse IgG antibody (Thermo Fisher Scientific A31571) or by magnetic cell sorting (MACS) using anti-mouse IgG MicroBeads (Miltenyi Biotec 130-047-302).

Induction and passages of iAT2 cells in FD-AOs

The isolated CPM^{hi} lung epithelial progenitor cells were differentiated into iAT2 cells in the FD-AOs in a 12-well plate with DCIK medium: Ham's F12 (Fujifilm Wako, 087-08335) containing 50 nM dexamethasone (Sigma-Aldrich, D4902), 100 μM 8-Br-cAMP (Biolog Life Science Institute, B007), 100 μM 3-isobutyl-1-methylxanthine (Fujifilm Wako, 099-03411), 10 ng/mL KGF, 1% B-27 supplement, 0.25% bovine albumin fraction V (Thermo Fisher Scientific,15260-037), 15 mM HEPES (Thermo Fisher Scientific, 17557-94), 0.8 mM $CaCl_2$ (Fujifilm Wako, 036-19731), 0.1% ITS premix (Corning, 354352), 50 U/mL Penicillin/Streptomycin, as described previously[2]. Briefly, 1×10^{4} CPM^{hi} cells and 5×10^{5} HFLF were mixed in 200 μL of 50%Growth Factor Reduced Matrigel (Corning 354230) diluted with DCIK+10 μM Y-27632 medium and seeded on a cell-culture insert. A 1 mL of DCIK medium was added to the lower chamber and supplemented with 10 μM Y-27632 for the first 3 days. The medium was changed every 2–3 days for two weeks. *SFTPC*^{GFP} reporter iPSC-derived iAT2 cells were isolated by gating $EpCAM^{+}/GFP^{+}$ cells, whereas ChiPSC18 iPSC-derived iAT2 cells were isolated by gating $EpCAM^{+}/NaPi2b^{+}$ cells using FACS as described previously [3]. Isolated iAT2 cells were reseeded for on-gel culture.

Isolation of epithelial cells from FD-AOs

The matrices of FD-AOs were collected in 15 mL tubes containing 0.1% Trypsin EDTA (Thermo Fisher Scientific 25200072), and spheroids were dissociated into single cells via pipetting. After neutralizing trypsin by adding twice the volume of Dulbecco's Modified Eagle's medium supplemented with 50 U/mL P–S and 10% fetal bovine serum, the cells were collected via centrifugation at 4 °C, 900 rpm for 5 min. $EpCAM^{+}$ epithelial cells were isolated by MACS using a mouse anti-human EpCAM antibody (Santa Cruz SC-66020) and anti-mouse IgG MicroBeads (Table S2).

Flow cytometry (FCM)

"On-gel" spheroids were incubated with cell recovery solution (Corning 354253) for 1 h at 4 °C. The spheroids were collected and dissociated into single cells using Accutase for 20 min. The cells were suspended in FCM buffer (1% BSA and 10 μM Y-27632 in PBS) containing SYTOX Blue Dead Cell Stain (Thermo Fisher Scientific S34857) and analyzed using FACS Aria III (BD Biosciences).

Live cell imaging and measurement of organoid morphology

Live-cell images of organoids were acquired using a BZ-X710 microscope (Keyence). The thickness of the epithelium and the diameter of the spheroids were measured in 20 randomly selected spheroids in each sample per test using the BZ-X Analyzer.

Transmission electron microscopy

As described previously (Gotoh et al., 2014), each sample was fixed in 0.1M phosphate buffer (pH 7.4) containing 0.1% picric acid, 2% paraformaldehyde, 2% osmium tetroxide, 2.5% glutaraldehyde, and 4% sucrose. En-bloc

staining was performed using 1% uranyl acetate solution. The specimens were dehydrated and embedded in Epon 812 (Nacalai Tesque). The ultrathin sections were stained with uranyl acetate and lead citrate. Transmission electron microscopy (Nihondenshi, JEM-1400Flash) was used for the examination.

mRNA expression analysis

Total RNA was extracted using the RNeasy Micro Kit (QIAGEN 74004) or RNeasy Mini Kit (QIAGEN 74106), according to the manufacturer's protocol. ReverTra Ace qPCR RT Master Mix with gDNA Remover (Toyobo FSQ-301) was used for the reverse transcription of RNA. Quantification was performed using THUNDERBIRD Probe qPCR Mix (Toyobo QPS101) or Power SYBR Green Master Mix (Thermo Fisher Scientific 4368706); and the primers used are listed in Tables S3 and S4. Gene expression was normalized to that of 18S rRNA. The expression of each gene was compared with that of the human adult lung five donor pool (BioChain #R1234152-P, lot A811037) or each control.

RNA-seq and bioinformatics analysis

RNA was extracted using the RNeasy Mini Kit or RNeasy Micro Kit, and its quality was checked using the Agilent RNA 6000 Pico Assay Kit (Agilent Technologies) according to the manufacturer's protocol. The sequence library was prepared using Illumina Stranded Total RNA Prep, ligation with Ribo-Zero Plus (Illumina), and the quality of the library was checked using a High Sensitivity D500 kit and 4200 TapeStation (Agilent). The sequence was performed using Illumina NextSeq2000. FASTQ files were created using bcl2fastq-2.20. Cut adapt ver v4.1 was used for trimming adaptor sequences and low-quality bases from the raw reads. The trimmed reads were mapped to the human reference genome sequence (hg38) using STAR version 2.7.10a and a GENCODE (GRCh38.p13, release 32) GTF file. Htseq-count ver. 2.0.2 was used for calculating the row counts with the GENCODE GTF file. Gene expression levels were calculated as transcripts per kilobase million (TPM) with DEseq2 v1.34.0. Low expression genes with average TPM values among the comparison data set of < 1 were excluded for the following analyses. The R package "maptools" was used for describing the principal component analysis (PCA) of $\log_2$ (TPM + 0.01). The R package DESeq2 (Love et al., 2014) was used to identify the DEGs between the two groups. The R package "gplots" was used for creating a heatmap based on $\log_2$ (TPM + 0.01). Venn diagram was created with the R package "VennDiagram". Enrichment analysis for gene ontology (GO) Biological Processes was analyzed using the Metascape online software, and the analysis was limited to a "summary" group of GO.

Immunofluorescence analysis

Each sample was fixed with 4% paraformaldehyde in PBS. After being washed in PBS, each sample was immersed in 30% sucrose in PBS, embedded in OCT compound (Sakura Finetek 45833), and frozen at -80°C. The sample blocks were sliced into 10 µm with cryotome (Leica), permeabilized in 0.2% Triton X-100, immersed in the blocking buffer (5% Donkey serum, 1% bovine serum albumin (BSA) (Sigma A9647) in PBS, and stained, as described previously [4]. Immunofluorescence images were obtained using a confocal microscope (Leica SP8).

Caspase-Glo 3/7 assay

Prior to the Caspase-Glo assay, the cell count was measured using CCK8, according to the manufacturer's instructions. The cells were incubated in the medium containing 10% CCK8 at 37°C for 1 h. Then, the medium was transferred into a new clear 96-well plate and its absorbance was measured. After the cells were washed once with the medium, the medium was replaced with 50 µL of fresh DCIK medium, and 50 µL of Caspase-Glo 3/7 reagent (Promega N8090) was added to each well. The plates were mixed on a shaker for 30 sec and incubated at room temperature for 30 min. Luminescence was measured using ARVO X5 plate reader (PerkinElmer), and luminescence values were corrected with the CCK8 absorbance values.

Two-dimensional (2D) culture

A 6-well cell culture plate was coated with an iMatrix-511 at the concentration of 0.5 µg/cm^2 for at least 1 hour under 37°C before cell seeding; 1×10^6 of MACS-sorted CPM^{hi} progenitor cells were diluted in the 2 mL of DCIK+10 µM Y-27632 medium and seeded on iMatrix-511 coated plate. Y-27632 was withdrawn from the medium on day 4. LATS-IN-1 was applied at 10 µM final concentration on day 4 and cells were harvested on day 7.

Western blotting

Spheroids cultured in on-gel or 2D cultured cells were washed with PBS and harvested with Matrigel using 2×Lysis buffer consisting of 2×RIPA buffer (Nacalai Tesque 08714-04), 2% SDS (Biorad 1610301), and 2×Phosphatase

Inhibitor Cocktail (Nacalai Tesque 07574-61). Cell extracts were centrifuged at 4 °C and 15,000 × g for 20 min. Each supernatant was collected, and protein concentration was measured using a BCA protein assay kit (Thermo Fisher Scientific 23227). Protein concentrations were matched between samples, and 4xLDS sample buffer (Thermo Fisher Scientific NP0007) supplemented with 2-mercaptoethanol was added to each sample and incubated at 70 °C for 10 min. Samples were loaded on 8% polyacrylamide gel, electrophoresed in Tris-Glycine SDS buffer (Biorad1610732), and transferred to PVDF membrane (0.2 μm Merck) in Tris-Glycine buffer (Biorad 1610734) supplemented with 20% methanol. The membrane was blocked with 50% Blocking One-P (Nacalai Tesque 05999-84) diluted with TBS-T (Takarabio T9142) for 30 min and incubated with primary antibodies (Table S2) diluted with Can Get Signal Immunoreaction Enhancer Solution 1 (TOYOBO NKB-101) at 4 °C overnight. On the following day, the membrane was washed with TBS-T three times and incubated with secondary antibodies diluted with Can Get Signal Immunoreaction Enhancer Solution 2 (TOYOBO NKB-301) for 1 h at room temperature and washed with TBS-T three times. Bands were detected using Pierce ELC plus Western Blotting Substrate (Thermo Fisher Scientific 32132) and images were acquired using ChemiDoc Touch MP. Image Lab software 6.1 was used to quantify the bands.

Primary adult human alveolar epithelial cell culture

Adult human alveolar epithelial cells were purchased from ScienCell Research Laboratories (cat. # 3240). To sustain and expand cells, 2-3×10^4 cells were mixed with 50 μL of Growth Factor Reduced Matrigel and generated a Matrigel dome; 2-3 domes were generated per well of a 6-well plate. After incubation at 37 °C for > 30 min, 2 mL of PneumaCult™ Alveolar Organoid Expansion Medium (AvOE) containing Passage Supplement (STEMCELL Technologies ST-100-0847) was added per well. The medium was changed to AvOE without Passage Supplement from day 3, and the medium was changed every 2-3 days, until day 14. On day 14, Matrigel domes were collected in 15 mL tubes containing 0.1% Trypsin EDTA, and spheroids were dissociated into single cells via pipetting. After neutralizing trypsin by adding twice the volume of Dulbecco's Modified Eagle's medium supplemented with 50 U/mL P–S and 10% fetal bovine serum, the cells were collected via centrifugation at 4 °C, 900 rpm for 5 min. Dissociated cells were stained with 1 μL of anti-HT2-280 antibody per million cells for 20 min on ice, and subsequently stained with AlexaFluor647-conjugated anti-mouse IgM for 20 min on ice. HT2-280 positive cells were isolated using FACS and used for subsequent experiments or passages. When compounds were tested, 1×10^4 primary human AT2 cells were seeded for on-gel culture with Advanced DMEM (Thermo Fisher Scientific 12634010) supplemented with 2% B-27 supplement, 0.5% P–S, 10 mM HEPES (Nacalai Tesque 17557-94), 1×Glutamax (Gibco 35050-061), 1 mM N-acetyl-L-Cysteine (Sigma Aldrich SLCB5087), 50 ng/mL EGF (Thermo Fisher Scientific PHG0314), 5 ng/mL KGF , 100 ng/mL FGF-basic (Peprotech PEP-100-18B), 100 ng/mL FGF10, 100 ng/mL IGF1 (Biolegend 590906), 100 ng/mL Neuregulin1 (Peptotech PEP-100-03), 100 ng/mL Noggin, 10 nM [Leu15]Gastrin1 (Sigma Aldrich G914), 500 nM A83-01 (Wako 039-24111), 20 nM R-spondin-1 (Peprotech 120-38), 0.2% Afamin-Wnt-3A serum-free conditioned medium (MBL Life Science J2-001), and 10 μM Y-27632 described previously[5]. Y-27632 was withdrawn after day 3. From day 6, A83-01, R-spondin-1, and Afamin-Wnt-3A serum-free conditioned medium were withdrawn. On day 9, the compounds were added to the medium for 3 days, and subsequent experiments were performed.

Ethics

All iPSC cell lines (B2-3, B2-3-dual-42-7, and ChiPSC18), primary AT2 cells and fetal lung fibroblasts were exempt from ethical approval.

SUPPLEMENTAL REFERENCES

1. Travaglini, K.J., Nabhan, A.N., Penland, L., Sinha, R., Gillich, A., Sit, R.V., Chang, S., Conley, S.D., Mori, Y., Seita, J., et al. (2020). A molecular cell atlas of the human lung from single-cell RNA sequencing. Nature *587*, 619–625.

2. Yamamoto, Y., Gotoh, S., Korogi, Y., Seki, M., Konishi, S., Ikeo, S., Sone, N., Nagasaki, T., Matsumoto, H., Muro, S., et al. (2017). Long-term expansion of alveolar stem cells derived from human iPS cells in organoids. Nat. Methods *14*, 1097–1106.

3. Korogi, Y., Gotoh, S., Ikeo, S., Yamamoto, Y., Sone, N., Tamai, K., Konishi, S., Nagasaki, T., Matsumoto, H., Ito, I., et al. (2019). In Vitro Disease Modeling of Hermansky-Pudlak Syndrome Type 2 Us- ing Human Induced Pluripotent Stem Cell-Derived Alveolar Organoids. Stem Cell Reports.

4. Suezawa, T., Kanagaki, S., Moriguchi, K., Masui, A., Nakao, K., Toyomoto, M., Tamai, K., Mikawa, R., Hirai, T., Murakami, K., et al. (2021). Disease modeling of pulmonary fibrosis using human pluripotent stem cell-derived alveolar organoids. Stem Cell Reports *16*, 2973–2987.

5. Ebisudani, T., Sugimoto, S., Haga, K., Mitsuishi, A., Takai-Todaka, R., Fujii, M., Toshimitsu, K., Hamamoto, J., Sugihara, K., Hishida, T., et al. (2021). Direct derivation of human alveolospheres for SARS-CoV-2 infection modeling and drug screening. Cell Rep. *35*, 109218.
